# External validation of 87 clinical prediction models supporting clinical decisions for breast cancer patients

**DOI:** 10.1016/j.breast.2023.04.003

**Published:** 2023-04-17

**Authors:** Tom A. Hueting, Marissa C. van Maaren, Mathijs P. Hendriks, Hendrik Koffijberg, Sabine Siesling

**Affiliations:** a Department of Health Technology & Services Research, Technical Medical Centre, University of Twente, Enschede, Netherlands; b Evidencio, Medical Decision Support, Haaksbergen, Netherlands; c Department of Research and Development, Netherlands Comprehensive Cancer Organisation (IKNL), Utrecht, Netherlands; d Department of Medical Oncology, Northwest Clinics, Alkmaar, Netherlands

**Keywords:** Breast cancer, Prediction models, External validation, Prognostic model, Nomogram

## Abstract

**Introduction:**

Numerous prediction models have been developed to support treatment-related decisions for breast cancer patients. External validation, a prerequisite for implementation in clinical practice, has been performed for only a few models. This study aims to externally validate published clinical prediction models using population-based Dutch data.

**Methods:**

Patient-, tumor- and treatment-related data were derived from the Netherlands Cancer Registry (NCR). Model performance was assessed using the area under the receiver operating characteristic curve (AUC), scaled Brier score, and model calibration. Net benefit across applicable risk thresholds was evaluated with decision curve analysis.

**Results:**

After assessing 922 models, 87 (9%) were included for validation. Models were excluded due to an incomplete model description (n = 262 (28%)), lack of required data (n = 521 (57%)), previously validated or developed with NCR data (n = 45 (5%)), or the associated NCR sample size was insufficient (n = 7 (1%)). The included models predicted survival (33 (38%) overall, 27 (31%) breast cancer-specific, and 3 (3%) other cause-specific), locoregional recurrence (n = 7 (8%)), disease free survival (n = 7 (8%)), metastases (n = 5 (6%)), lymph node involvement (n = 3 (3%)), pathologic complete response (n = 1 (1%)), and surgical margins (n = 1 (1%)). Seven models (8%) showed poor (AUC<0.6), 39 (45%) moderate (AUC:0.6–0.7), 38 (46%) good (AUC:0.7–0.9), and 3 (3%) excellent (AUC≥0.9) discrimination. Using the scaled Brier score, worse performance than an uninformative model was found in 34 (39%) models.

**Conclusion:**

Comprehensive registry data supports broad validation of published prediction models. Model performance varies considerably in new patient populations, affirming the importance of external validation studies before applying models in clinical practice. Well performing models could be clinically useful in a Dutch setting after careful impact evaluation.

## Highlights

• A total of 87 prediction models for breast cancer patients were externally validated using data of 271,040 Dutch patients.
• Assessing model performance on discrimination and calibration, 34 models performed well, 26 moderately, and 27 poorly.
• Validating models is crucial before clinical use. A high-performing model is ideal, but assessing actual benefit is preferable.

## 1 Introduction

Worldwide, over 2.2 million new cases of breast cancer were diagnosed in 2020 [1]. In the Netherlands, over 17,000 women and 100 men are diagnosed with breast cancer annually, making this the most commonly diagnosed cancer in women [2]. Even though breast cancer survival has improved throughout the past decades, the prognosis of an individual breast cancer patient strongly depends on patient- and tumor-related characteristics, and available treatment options [3].

To support (shared) decision-making by patients and clinicians regarding breast cancer treatment, prediction models have been developed that estimate the probability of certain outcomes using available patient- and tumor-related characteristics. An example of such a model is PREDICT [4], which is frequently used to support clinical decision-making on adjuvant systemic therapy.

Previously, a systematic literature review was performed to identify available prediction models that may provide valuable information to support treatment decision-making [5]. A total of 922 available prediction models were identified, which were developed to predict clinical outcomes such as treatment response, lymph node involvement, adverse events, recurrence, and (breast cancer-specific) survival. However, the majority of the identified models were found to be at high risk of bias according to the Prediction Model Risk Of Bias Assessment Tool (PROBAST) [6]. The clinical utility of most of these models remained unclear as a substantial number of models were not reported according to established reporting guidelines or showed methodological flaws during the development and/or the internal validation of the model.

Prior to the use of prognostic models in a clinical setting, they should be validated both internally and externally on the target population [7]. Moreover, the clinical impact of the models on clinical practice should subsequently be assessed [8]. Still, for meaningful applications of prediction models, new models are more often developed than existing models are externally validated, and impact studies are performed even less, which means that potentially valuable information on the performance of a model is lacking [9]. This refrains existing models from being implemented in daily practice to support clinical decision-making in a certain population. However, when already available prediction models perform well on external data sets, the creation of new models will become less relevant than actually implementing valuable and validated models, and keeping these up to date [10]. Therefore, this study aims to evaluate the performance of previously identified prediction models using readily available data obtained from the Netherlands Cancer Registry (NCR).

## 2 Methods

### 2.1 Study population

The performance of identified clinical prediction models was evaluated using data obtained from the NCR. The NCR is a nationwide database comprising all newly diagnosed malignant tumors in the Netherlands. The data cohort consisted of patients diagnosed with breast cancer between 2003 and 2019. Invasive and non-invasive cancers were included, as well as female and male breast cancer patients. Patients were excluded if they were younger than 18 years old, or when the cancer was diagnosed during an autopsy.

Based on the patient group targeted by a prediction model, specific subgroups of patients were extracted from the full dataset to perform the model validation. To validate the different models, the definition of included variables, and the inclusion and exclusion criteria were applied as described in the original paper as much as possible.

### 2.2 Model selection

The previously identified 922 clinical prediction models, described in 534 papers were considered to be potential candidates for external validation and were selected based on four criteria.

First, models were selected in case sufficient details were reported to recover the underlying equation allowing the calculation of risks of the outcome for individual patients. For this, the underlying variable coefficients required to calculate the result of a model had to be available (or could be recovered from a nomogram), and all required covariates (input variables and outcome) should have been clearly defined.

Second, the required data, including both the input and outcome variables, for adequate validation of the model had to be available in the NCR.

Third, models were excluded when they were either developed by or previously validated on NCR data.

Fourth, models were excluded in case the available sample size within the NCR to validate the model was too low. For sample size considerations, the 100 events and non-events rule-of-thumb reported by Vergouwe et al. was initially used [11]. When the sample size was lower than 100 events and non-events (e.g. indicating a minimal requirement of 200 patients when the outcome occurs in 50% of the patients), additional calculations were performed according to the study by Riley et al. to determine if available data allowed validation [12].

Several assumptions were made in the data to allow more models to be validated. As the cause of death is not recorded in the NCR, patients who died with known metastatic breast cancer were assumed to have died due to breast cancer. The breast cancer subtype definition varies in different models. When no clear definition was provided in the paper describing the development of the model, the following definition was applied for breast cancer subtype; Luminal A (HR+ & HER2-), Luminal B (HR+ & HER2-), HER2-enriched (HR- & HER2+), and triple negative (HR- & HER2-). For models predicting a time-to-event outcome that may occur more than once (e.g. metastasis or locoregional recurrence), only the first event that occurred was taken into account.

### 2.3 Statistical analysis

All models were assessed on their performance in terms of discrimination, calibration, and net benefit. Discrimination concerns the ability of a model to stratify between high and low risk of the predicted outcome, and was quantified with the area under the receiving operating characteristic curve (AUC), and visualized using classification plots as proposed by Verbakel et al. [13] Discriminatory performance was considered poor (AUC<0.6), moderate (AUC:0.6–0.7), good (AUC:0.7–0.9), and excellent (AUC≥0.9). Calibration concerns the level of agreement between predicted and observed event rates and is visualized using calibration plots. Also, the Brier score and the scaled Brier score were estimated for each model. The Brier score concerns the squared differences between predicted and observed outcomes [14]. Brier scores range between 0 and 1, and a lower Brier score indicates better performance. The scaled Brier score compares the Brier score to the Brier score of an uninformative model (i.e. assuming the observed event rate is the predicted risk for all patients). A scaled Brier score <0 indicates that the model performs worse than an uninformative model. A higher scaled Brier score indicates better performance. A combination of the AUC and the scaled Brier score was used to categorize the overall performance of the models into poor (AUC<0.7 and scaled Brier≤0), moderate (either an AUC≥0.7 or a scaled Brier>0), and good (AUC≥0.7 and scaled Brier>0). Clinical usefulness was assessed by comparing the net benefit of applying the model over all feasible thresholds, and is visualized using decision curve analysis in which the added value of the model is compared to default strategies of treating all or no patients [15].

A separate dataset was created based on the original in- and exclusion criteria reported for each of the validated models. Missing data were assessed for each separate dataset and where appropriate, missing data were handled using multiple imputation by chained equations (MICE) [16]. Missing data were imputed on the complete dataset to ensure accurate estimations. The process of data imputation and model performance evaluation was repeated using 200 bootstrap samples.

## 3 Results

### 3.1 Patient data

Data on 288,784 tumors diagnosed in 271,040 patients were obtained from the NCR. Patient characteristics from the data obtained from the NCR are displayed in Table 1. The majority of the patients were female (n = 287,000 (99.4%)). On average, patients were 61 (SD 13.7) years old when diagnosed. The number of tumors increased over the years ranging from 121,884 (42%) in 2003–2010 to 166,900 (58%) in 2011–2019. From the dataset of 288,784 breast tumors, smaller cohorts were selected according to the in- and exclusion criteria of the model being validated. For each of the validated models, detailed descriptions of the outcome, input variables, inclusion criteria, exclusion criteria, original validation, and baseline characteristics of the dataset used to validate each of the included models were summarized in the supplementary data. The sample size used to validate a model ranged between 432 and 243,930 with a median sample size of 10,368 (IQR 5808–47,875).

**Table 1:** Patient characteristics of all breast cancer patients derived from the NCR (n = 288,784).

Characteristic	Value	N	(%)
Total		**288,784**	**100%**
Gender	Male	1784	0.6**%**
	Female	287,000	99.4**%**
Age	Years (Mean (SD))	61 (13.7)	13.7
Year of diagnosis	2003–2006	57,539	19.9**%**
	2007–2010	64,345	22.3**%**
	2011–2014	72,526	25.1**%**
	2015–2019	94,374	32.7**%**
Malignancy	Invasive carcinoma	254,395	88.1**%**
	Carcinoma in situ	34,389	11.9**%**
Stage^a^	0	34,389	11.9**%**
	I	113,420	39.3**%**
	II	95,496	33.1**%**
	III	30,825	10.7**%**
	IV	13,420	4.6**%**
	Missing	1234	0.4**%**
Differentiation grade	1	56,999	19.7**%**
	2	113,530	39.3**%**
	3	76,891	26.7**%**
	Missing	41,364	14.3**%**
ER status	Negative	40,349	14.0**%**
	Positive	203,545	70.5**%**
	Missing	44,890	15.5**%**
PR status	Negative	77,977	27.0**%**
	Positive	161,881	56.1**%**
	Missing	48,926	16.9**%**
HER2 status	Negative	186,141	64.5**%**
	Positive	29,917	10.4**%**
	Unclear	22,039	7.6**%**
	Missing	50,687	17.5**%**
Follow-up data regarding recurrences completely available over^b^:	5-year	62,116	21.5**%**
	10-year	20,858	7.2**%**

a Stage was defined as the pathologic tumor stage, supplemented by clinical tumor stage (when pathologic stage was unknown or when the patient received neoadjuvant treatment).

b The follow-up data was actively searched for certain cohorts only in the NCR and therefore does not reflect the lost to follow-up rate.

### 3.2 Model selection

**Fig. 1:**
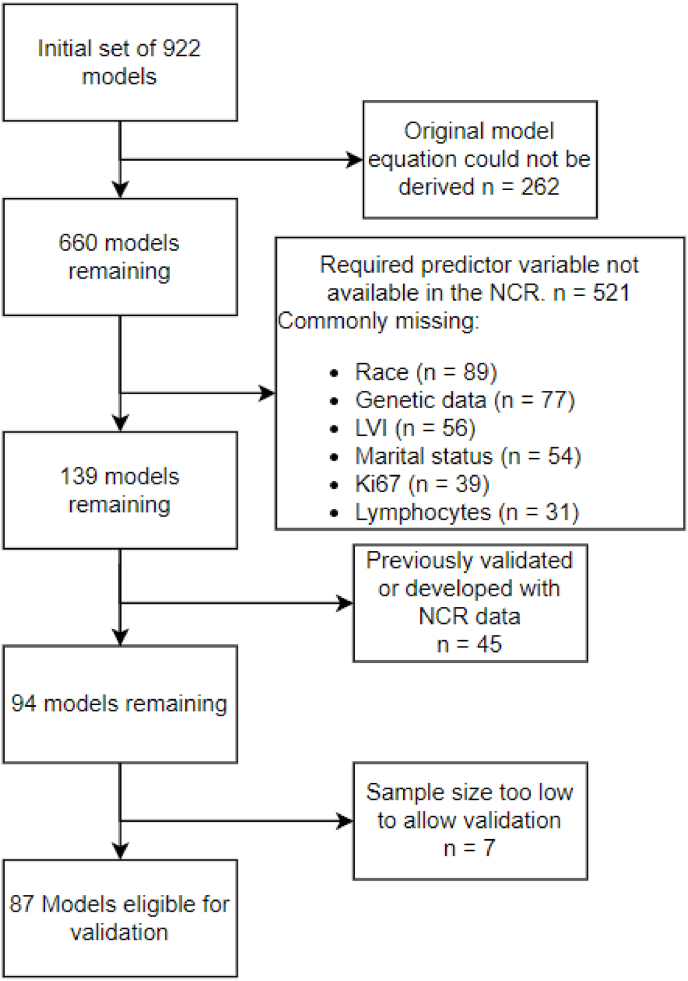
Flowchart of inclusion and exclusion criteria. Abbreviations: LVI = Lymphovascular invasion, NCR = Netherlands Cancer Registry.

All 922 models were initially considered for inclusion in our study. A total of 262 (28%) models were not described with sufficient details to calculate a risk for new patients (e.g. the original model equation could not be derived due to lack of reported model coefficients) and could not be validated. Another 521 (57%) models were excluded due to the unavailability of required input or outcome data in the NCR. Data most commonly resulting in the exclusion of a model were, race (n = 89), genetic data (n = 77), lymphovascular invasion (LVI) (n = 56), marital status (n = 54), Ki67 (n = 39), and lymphocytes (including tumor infiltrating lymphocytes and indices such as monocyte-to-lymphocyte ratio) (n = 31). Models developed or previously validated with NCR data (n = 45 (5%)) were also excluded, and lastly, 7 (1%) models were excluded as the available sample size was too low to validate these models. Finally, a total of 38 papers reporting on a total of 87 (9%) models were included in our external validation study. The process of in- and excluding the models is visualized in the flowchart in Fig. 1.

An overview of the included models is provided in Table 2. A total of 33 (38%) models were developed to predict overall survival (OS), 27 (31%) models predicted breast cancer-specific survival (BCSS), 3 (3%) models other cause specific survival (OCSS), 7 (8%) models disease free survival (DFS), 7 (8%) locoregional recurrence (LRR), 5 (6%) predicted metastasis, 3 (3%) models lymph node involvement (LNI), 1 (1%) model pathologic complete response (PCR), and 1 (1%) model predicted surgical margin status. Several models were developed for a specific subset of patients. For instance, the models developed by Chen et al. (models 19a & 19b), were specifically aimed to provide BCSS predictions for male breast cancer patients. A short description of the specific patient subgroups per model is displayed in Table 2 and more detailed descriptions can be found in the supplementary tables.

**Table 2:** Overview of the validated models, predictors, events, and population, grouped by outcome.

Author	Model ID	Specific patient sub-group	Input variables	Outcome	Original AUC^a^	AUC	Scaled Brier score	Sample size	Event rate
Overall survival									
Xiong	1a	M1	Age, MFI, M, HR	1-year	0.670	0.668 (0.656–0.678)	−0.033 (−0.043–−0.024)	11,633	71.7%
Xiong	1b	M1	Age, MFI, M, HR	3-year	0.670	0.652 (0.642–0.661)	0.010 (−0.003 – 0.025)	10,964	37.8%
Regierer	2	M1	MFI, HR, M	5-year	0.686	0.622 (0.614–0.631)	−0.065 (−0.079–−0.050)	17,608	23.9%
Fan	3a	Mast	Age, T, N, M, ER	2-year	0.800	0.665 (0.658–0.673)	−0.123 (−0.137–−0.108)	86,418	93.5%
Fan	3b	Mast	Age, T, N, M, ER	5-year	0.800	0.683 (0.678–0.687)	−0.013 (−0.024–−0.004)	73,465	79.1%
Luo	4a	M0 & HER2+	Age, ER, T, N, Tras	3-year	0.780 & 0.740	0.619 (0.598–0.636)	−0.005 (−0.013 – 0.005)	15,107	93.5%
Luo	4b	M0 & HER2+	Age, ER, T, N, Tras	5-year	0.780 & 0.740	0.597 (0.583–0.610)	−0.029 (−0.038–−0.018)	13,599	87.6%
Zhang	5a	Adj Rad	Age, Gr, T, N, ER, PR	5-year	0.687 & 0.672	0.726 (0.703–0.747)	0.078 (0.059–0.096)	3208	84.1%
Zhang	5b	Adj Rad	Age, Gr, T, N, ER, PR	10-year	0.687 & 0.672	0.672 (0.650–0.699)	0.008 (−0.023 – 0.043)	2072	60.0%
Zhang	5c	No Rad	Age, Gr, T, N ER, PR	5-year	0.700 & 0.696	0.715 (0.702–0.731)	0.067 (0.054–0.080)	10,423	87.4%
Zhang	5d	No Rad	Age, Gr, T, N ER, PR	10-year	0.700 & 0.696	0.711 (0.700–0.723)	0.106 (0.092–0.118)	9254	61.5%
Chen	6a	M0	Age, Gr, T, N, HR	5-year	0.822 & 0.780	0.696 (0.692–0.700)	0.047 (0.041–0.052)	170,643	85.9%
Chen	6b	M0	Age, Gr, T, N, HR	5-year	0.792 & 0.800	0.622 (0.618–0.626)	0.007 (0.003–0.010)	170,643	85.9%
Zhao	7a	Advanced	TNM, MS, DFS, TB, BM	1-year	0.770 & 0.710	0.731 (0.720–0.741)	−0.437 (−0.463–−0.415)	8745	63.7%
Zhao	7b	Advanced	TNM, MS, DFS, TB, BM	2-year	0.770 & 0.710	0.750 (0.740–0.760)	−0.541 (−0.574–−0.503)	8743	45.6%
Zhao	7c	Advanced	TNM, MS, DFS, TB, BM	3-year	0.770 & 0.710	0.776 (0.765–0.787)	−0.593 (−0.635–−0.547)	8740	33.7%
Tang	8a	T1-2N1M0	Age, Topo, T, N ER, PR, HER2, Tras	5-year	0.700	0.650 (0.638–0.663)	−0.129 (−0.138–−0.116)	8774	82.2%
Tang	8b	T1-2N1M0	Age, Topo, T, N ER, PR, HER2, Tras	10-year	0.700	0.604 (0.591–0.618)	−0.369 (−0.396–−0.346)	7238	62.1%
Xu	9a	Stage I-II	Age, Gr, T, MS, SRG	3-year	0.802	0.775 (0.770–0.779)	0.060 (0.057–0.063)	175,927	94.0%
Xu	9b	Stage I-II	Age, Gr, T, MS, SRG	4-year	0.795	0.769 (0.766–0.774)	0.067 (0.064–0.071)	161,550	90.8%
Xu	9c	Stage I-II	Age, Gr, T, MS, SRG	5-year	0.787	0.763 (0.760–0.767)	0.067 (0.063–0.070)	147,892	87.2%
Wang	10a	Bone M1	Gr, Morf, T, SRG, Chem, M, MS	3-year	0.705 & 0.678	0.665 (0.650–0.677)	0.070 (0.049–0.086)	5834	46.0%
Wang	10b	Bone M1	Gr, Morf, T, SRG, Chem, M, MS	5-year	0.705 & 0.678	0.663 (0.646–0.682)	0.044 (0.025–0.071)	5375	23.3%
Zheng	11a	M1 pre-op	Age, Gr, T, M, ER, PR, HER2, Rad, Chem	1-year	0.721	0.701 (0.689–0.714)	0.084 (0.074–0.095)	8409	75.3%
Zheng	11b	M1 pre-op	Age, Gr, T, M, ER, PR, HER2, Rad, Chem	3-year	0.721	0.703 (0.694–0.714)	0.081 (0.066–0.099)	7577	40.0%
Zheng	11c	M1 SRG	Age, Gr, T, M, ER, PR, HER2, Rad, Chem	1-year	0.713	0.786 (0.757–0.818)	0.011 (−0.062 – 0.074)	1994	90.5%
Zheng	11d	M1 SRG	Age, Gr, T, M, ER, PR, HER2, Rad, Chem	3-year	0.713	0.735 (0.714–0.759)	0.143 (0.110–0.181)	1769	59.2%
Zheng	11e	M1 no-SRG	Age, Gr, T, M, ER, PR, HER2, Rad, Chem	1-year	0.664	0.691 (0.675–0.704)	0.087 (0.067–0.104)	6415	70.5%
Zheng	11f	M1 no-SRG	Age, Gr, T, M, ER, PR, HER2, Rad, Chem	3-year	0.664	0.678 (0.661–0.691)	0.076 (0.056–0.091)	5808	34.1%
Janssen	12a	Bone M1	ECOG, M	1-year	NA	0.630 (0.573–0.678)	−0.095 (−0.224 – 0.008)	520	81.5%
Janssen	12b	Bone M1	ECOG, M	2-year	NA	0.657 (0.611–0.706)	0.058 (−0.019 – 0.159)	432	37.3%
Wang	13a	M0, mast, no neo-adj	Age, T, N, Gr, ER, PR	3-year	0.740	0.750 (0.745–0.756)	0.024 (0.021–0.027)	71,758	90.0%
Wang	13b	M0, mast, no neo-adj	Age, T, N, Gr, ER, PR	5-year	0.720	0.737 (0.731–0.742)	0.043 (0.038–0.048)	65,171	80.9%
**Breast cancer-specific survival**									
Abdel Rahman	14	M1 BC	M, ER, PR, HER2, Gr	4-year	0.665	0.666 (0.657–0.675)	−0.174 (−0.199–−0.149)	10,651	27.8%
Elwood	15	NA	HER2, Morf, Age, Etn, M, T, HR, Gr, N	10-year	0.840	0.740 (0.733–0.745)	0.116 (0.105–0.125)	48,661	13.7%
Paredes Aracil	16a	NA	Age, TNM, Gr, PBC, MF	5-year	0.830	0.911 (0.908–0.913)	0.002 (−0.011 – 0.017)	195,349	6.5%
Paredes Aracil	16b	NA	Age, TNM, Gr, PBC, MF	10-year	0.830	0.877 (0.874–0.881)	0.019 (0.004–0.036)	113,615	13.8%
Wen	17a	M0, IDC or ILC	Men, T, N, ER, HER2	5-year	0.747 & 0.789	0.641 (0.628–0.653)	−0.591 (−0.634–−0.548)	45,517	95.1%
Wen	17b	M0, IDC or ILC	Men, T, N, ER, HER2	10-year	0.747 & 0.789	0.650 (0.639–0.660)	−0.465 (−0.501–−0.433)	35,270	90.6%
Wen	18a	M0, IDC or ILC	ER, HER2, T, N, Men	5-year	0.745 & 0.796	0.642 (0.623–0.656)	−0.239 (−0.267–−0.209)	41,122	95.8%
Wen	18b	M0, IDC or ILC	ER, HER2, T, N, Men	10-year	0.745 & 0.796	0.647 (0.634–0.658)	−0.157 (−0.182–−0.134)	26,164	90.4%
Zhang	5e	Adj radio	Age, Gr, T, N ER, PR	5-year	0.699 & 0.656	0.758 (0.716–0.799)	−0.039 (−0.102 – 0.005)	2822	95.6%
Zhang	5f	Adj radio	Age, Gr, T, N ER, PR	10-year	0.699 & 0.656	0.702 (0.667–0.735)	0.033 (−0.011 – 0.069)	1433	86.8%
Zhang	5g	Adj radio	Age, Gr, T, N ER, PR	5-year	0.716 & 0.671	0.801 (0.780–0.820)	0.006 (−0.027 – 0.032)	9483	96.0%
Zhang	5h	Adj radio	Age, Gr, T, N ER, PR	10-year	0.716 & 0.671	0.751 (0.731–0.772)	0.023 (−0.007 – 0.051)	7258	78.5%
Chen	19a	Male	Age, T, ER, PR, SRG	3-year	0.788	0.827 (0.782–0.867)	0.078 (0.010–0.150)	1330	94.3%
Chen	19b	Male	Age, T, ER, PR, SRG	5-year	0.825	0.789 (0.752–0.832)	0.112 (0.055–0.182)	991	89.6%
Fu	20a	ILC, stage II-IV	Age, Topo, Gr, TNM, SRG, Chem, MS	3-year	0.793 & 0.830	0.926 (0.911–0.936)	0.389 (0.358–0.419)	12,246	94.1%
Fu	20b	ILC, stage II-IV	Age, Topo, Gr, TNM, SRG, Chem, MS	5-year	0.772 & 0.824	0.900 (0.889–0.912)	0.491 (0.466–0.518)	9849	89.0%
Xu	9d	Stage I-II	Age, Gr, T, MS, SRG	3-year	0.830	0.818 (0.808–0.828)	−0.092 (−0.105–−0.079)	168,847	99.1%
Xu	9e	Stage I-II	Age, Gr, T, MS, SRG	4-year	0.817	0.796 (0.788–0.803)	−0.086 (−0.099–−0.077)	151,702	98.5%
Xu	9f	Stage I-II	Age, Gr, T, MS, SRG	5-year	0.803	0.774 (0.766–0.781)	0.014 (0.011–0.018)	135,451	97.8%
Wang	10c	Bone M1	Gr, Morf, T, SRG, Chem, M, MS	3-year	0.710 & 0.684	0.663 (0.652–0.677)	0.066 (0.050–0.085)	5834	46.0%
Wang	10d	Bone M1	Gr, Morf, T, SRG, Chem, M, MS	5-year	0.710 & 0.684	0.661 (0.642–0.677)	0.036 (0.009–0.059)	5375	23.3%
Zheng	11g	M1 pre-op	Age, Gr, T, M, ER, PR, HER2, Rad, Chem	1-year	0.722	0.708 (0.695–0.723)	0.083 (0.070–0.098)	8409	75.3%
Zheng	11h	M1 pre-op	Age, Gr, T, M, ER, PR, HER2, Rad, Chem	3-year	0.722	0.711 (0.697–0.722)	0.097 (0.076–0.114)	7577	40.0%
Zheng	11i	M1 SRG	Age, Gr, T, M, ER, PR, HER2, Rad, Chem	1-year	0.715	0.791 (0.758–0.822)	0.083 (0.038–0.132)	1994	90.5%
Zheng	11j	M1 SRG	Age, Gr, T, M, ER, PR, HER2, Rad, Chem	3-year	0.715	0.742 (0.718–0.764)	0.169 (0.131–0.203)	1769	59.2%
Zheng	11k	M1 no-SRG	Age, Gr, T, M, ER, PR, HER2, Rad, Chem	1-year	0.666	0.694 (0.680–0.707)	0.098 (0.084–0.112)	6415	70.5%
Zheng	11l	M1 no-SRG	Age, Gr, T, M, ER, PR, HER2, Rad, Chem	3-year	0.666	0.680 (0.664–0.693)	0.084 (0.067–0.101)	5808	34.1%
**Other cause-specific survival**									
Xu	9g	Stage I-II	Age, Gr, T, MS, SRG	3-year	0.813	0.749 (0.744–0.754)	0.049 (0.046–0.052)	168,847	98.8%
Xu	9h	Stage I-II	Age, Gr, T, MS, SRG	4-year	0.808	0.747 (0.743–0.752)	0.059 (0.056–0.062)	151,702	98.2%
Xu	9i	Stage I-II	Age, Gr, T, MS, SRG	5-year	0.817	0.747 (0.743–0.751)	0.067 (0.063–0.069)	135,451	97.4%
**Locoregional recurrence**									
Herrero-Vicent	21	Neo-adj chem	HER2, DCIS, PCR	6-year	NA	0.583 (0.544–0.617)	−0.124 (−0.170–−0.088)	739	23.4%
Wobb	22	BCS, adj rad	Age, Men, Mar, ER, Gr	5-year	0.641	0.478 (0.448–0.565)	−1.996 (−2.235–−1.767)	11,822	2.3%
Sanghani	23	BCS	Age, LVI, Mar, T, Gr, Chem, Horm, Rad	10-year	0.660	0.592 (0.566–0.617)	0.006 (−0.003 – 0.016)	7343	6.8%
Li	24	T1-2N1-3M0	Age, Topo, N T, MS	5-year	0.735 & 0.703	0.619 (0.558–0.682)	−0.309 (−0.435–−0.216)	2886	3.0%
Corso	25a	Mast, no neo-adj	Age, Morf, T, N, MS, Horm, Chem, Rad	1-year (local)	0.700	0.765 (0.728–0.801)	0.016 (0.007–0.024)	22,882	0.7%
Corso	25b	Mast, no neo-adj	Age, Morf, T, N, MS, Horm, Chem, Rad	5-year (local)	0.700	0.689 (0.668–0.708)	0.037 (0.029–0.045)	18,498	4.0%
Corso	25c	Mast, no neo-adj	Age, Morf, T, N, MS, Horm, Chem, Rad	10-year (local)	0.700	0.679 (0.661–0.697)	0.038 (0.028–0.048)	15,173	5.6%
**Disease-free survival**									
Li	26	BCS	MS, Gr, N	5-year	0.700	0.610 (0.604–0.616)	−0.066 (−0.073–−0.060)	44,176	23.1%
Tokatli	27	M0	N, HER2, ER	5-year	0.700 & 0.715	0.633 (0.626–0.638)	−0.180 (−0.193–−0.166)	58,568	87.5%
Lin	28a	Age ≤40	N, MS	1-year	NA	0.692 (0.647–0.738)	−1.488 (−1.839–−1.218)	5127	97.6%
Lin	28b	Age ≤40	N, MS	2-year	NA	0.693 (0.665–0.722)	−1.406 (−1.633–−1.225)	4919	91.7%
Lin	28c	Age ≤40	N, MS	3-year	NA	0.684 (0.660–0.710)	−1.608 (−1.811–−1.442)	4759	87.5%
Paredes Aracil	29a	M0	Age, TNM, MF, Gr	5-year	0.750	0.718 (0.707–0.729)	0.073 (0.063–0.085)	21,653	12.0%
Paredes Aracil	29b	M0	Age, TNM, MF, Gr	10-year	0.750	0.692 (0.678–0.705)	0.018 (0.001–0.034)	7750	25.5%
**Metastatic disease**									
Dowsett	30	Postmenopausal, HR+	T, N Age, Gr	5–10 year	0.678	0.574 (0.540–0.604)	−0.107 (−0.143–−0.080)	5716	5.5%
Lin	31	M1 BC	Sex, Age, Morf, N, Gr, ER, PR, HER2	Liver metastasis	0.660 & 0.650	0.652 (0.641–0.663)	0.056 (0.048–0.066)	10,312	24.7%
Lim	32a	Adj rad	Age, MS, T, N	5-year	0.812	0.748 (0.738–0.759)	0.026 (0.019–0.035)	24,464	90.8%
Lim	32b	Adj rad	Age, MS, T, N	10-year	0.812	0.735 (0.722–0.746)	−0.273 (−0.291–−0.256)	8601	68.6%
Boutros	33	Invasive BC	T, N, ER, PR	M1	0.861 & 0.638	0.783 (0.780–0.788)	0.028 (0.025–0.032)	243,930	4.7%
**Axillary lymph node involvement**									
Zhang	34	T1-T3	Age, Top, N, T, Morf, MS	ALNI	0.716 & 0.701	0.696 (0.687–0.704)	−0.168 (−0.196–−0.147)	12,873	77.9%
Meretoja	35	Micro or ITC SLN	MF, T	ALNI	0.682	0.596 (0.581–0.614)	−0.052 (−0.062–−0.039)	5601	16.0%
Houvanaeghel	36	cN-	Age, T, Morf, Gr, MS	ALNI	0.682 & 0.686	0.622 (0.619–0.625)	−0.101 (−0.106–−0.095)	164,213	24.3%
**Pathologic complete response**									
Schipper	37	cN+	T, Morf, ER, PR, HER2, Tras, Chem	PCR	0.770	0.674 (0.662–0.684)	0.039 (0.023–0.056)	13,422	29.0%
**Positive surgical margin**									
Pan	38	BCS	HR, HER2, T, N, MF	Surgical margin	0.720 & 0.690	0.566 (0.562–0.570)	−0.064 (−0.068–−0.060)	113,499	17.5%

Abbreviations: Adj = Adjuvant, ALNI = Axillary Lymph Node Invasion, BM = Brain Metastasis, Chem = Chemotherapy, DFS = Disease Free Survival, ER = Estrogen Receptor status, Etn = Ethnicity, Gr = Grade, HER2 = HER2 status, Horm = hormonal therapy, HR = Hormone Receptor status, Mar = Surgical Margin, Mast = Mastectomy, Men = Menopausal status, MF = Multifocality, MFI = Metastasis Free Interval, Morf = Morfology, MS = Molecular Subtype, M = Metastasis, N = Nodal stage, PBC = Previous Breast cancer, PCR = Pathologic Complete Response, PR = Progesterone Receptor status, Rad = Radiotherapy, SRG = Surgery, T = Tumor size/stage, TB = Tumor burden, TNM = Stage, Top = Tumor Topography, Tras = Trastuzumab.

a Two values for the original AUC were displayed when the original model validation was assessed in multiple cohorts, using e.g. split sample or internal and external datasets.

### 3.3 Model performance evaluation

The performance of 87 models was evaluated. For each model, the AUC, and (scaled) Brier score were calculated, and a calibration plot, classification plot, and decision curve were visualized graphically (Supplementary data).

**Fig. 2:**
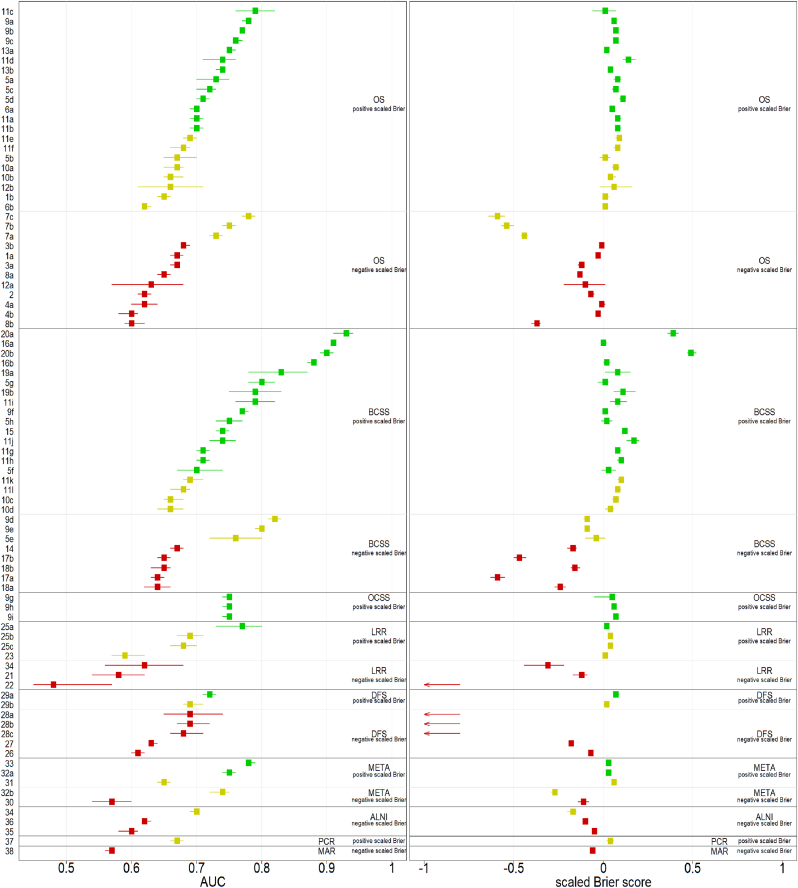
Visualization of the discrimination (AUC) and the scaled Brier score for each of the validated models. The green points represent models that were considered to perform good (AUC ≥0.7 and scaled Brier score >0), yellow corresponds with a moderate performance (AUC <0.7 or scaled Brier ≤0), and red is associated with a poor performance (AUC <0.7 and scaled Brier score ≤0). The model performance is presented per predicted outcome, and further divided by positive and negative scaled Brier. Abbreviations: ALNI = Axillary Lymph Node Involvement, AUC = Area Under the Curve, BCSS = Breast Cancer Specific Survival, DFS = Disease Free Survival, MAR = Positive Surgical Margin, META = Metastasis, LRR = Locoregional Recurrence, OCSS = Other Cause Specific Survival, OS = Overall Survival, PCR = Pathologic Complete Response. (For interpretation of the references to colour in this figure legend, the reader is referred to the Web version of this article.)

Summary measures including the AUC, scaled Brier score, sample size used, and the event rate for each model are additionally shown in Table 2. The AUC values ranged between 0.48 and 0.93. In terms of discrimination, 7 (8%) models had a poor (AUC<0.6), 39 (45%) models a moderate (AUC:0.6–0.7), 38 (44%) models a good (AUC:0.7–0.9), and 3 (3%) models an excellent (AUC≥0.9) performance on the AUC. The scaled Brier score ranged between −2.00 and 0.52 and showed an adequate performance (scaled Brier score >0) in 53 (61%) models, and a poor performance (scaled Brier score ≤0) in 34 (39%) models. Combining both measures resulted in 34 (39%) models showing a good performance (AUC ≥0.7 and scaled Brier score >0), 26 (30%) models showed a moderate performance (either an AUC<0.7 or scaled Brier score ≤0), and the remaining 27 (31%) models showed a poor performance (AUC<0.7 and scaled Brier score ≤0). The AUC and scaled Brier scores per model are described in Table 2 and visualized in Fig. 2.

**Fig. 3:**
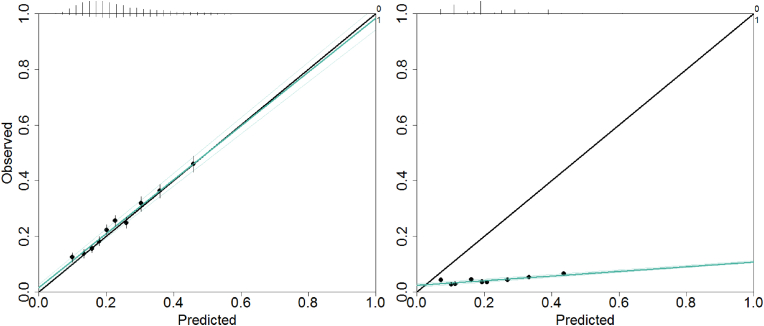
Examples of calibration plots to visualize the calibration. The black 45° line is the reference line and indicates perfect calibration. The green line is the fitted regression line. The small bars on top of the plot display a histogram of predicted risks. A taller bar represents more frequently predicted risks. The bars are stratified by 0 (non-events, displayed above the line) and 1 (events, displayed below the line). Depicted examples show good calibration (Left: model 31) and poor calibration (Right: model 22). (For interpretation of the references to colour in this figure legend, the reader is referred to the Web version of this article.)

**Fig. 4:**
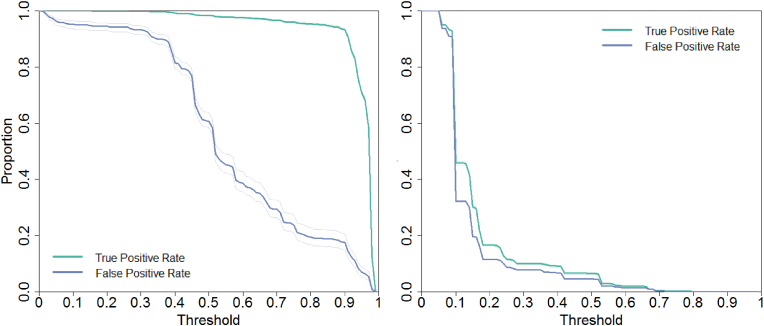
Examples of classification plots to visualize discrimination. The green line is the true positive rate (sensitivity) and the purple line represents the false positive rate (1- specificity). The left plot concerns a model with high discrimination (model 20a with AUC = 0.926) and the right is an example of a model with barely any discriminatory power (model 38 with AUC = 0.566). (For interpretation of the references to colour in this figure legend, the reader is referred to the Web version of this article.)

**Fig. 5:**
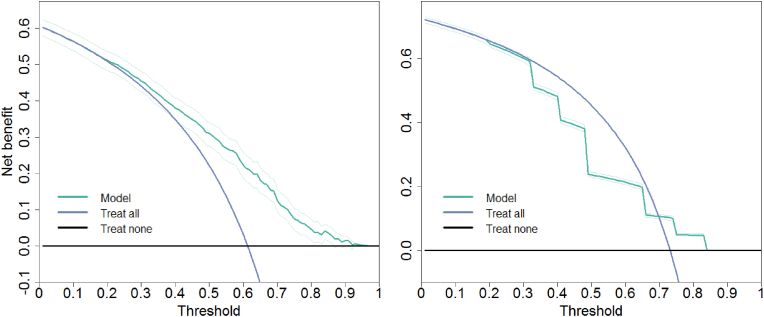
Examples of decision curves visualizing the net benefit. Green line = model, purple line = treat all, black line = treat nobody. The Left curve is an example of a model with mostly higher net benefit than default strategies (model 11k) and the figure on the right shows a model with barely any added net benefit compared to default strategies (model 14). (For interpretation of the references to colour in this figure legend, the reader is referred to the Web version of this article.)

A calibration plot, classification plot, and net benefit curve were constructed for each validated model and are displayed in the supplementary data. For illustrative purposes, examples of two calibration plots, classification plots and net benefit curves were displayed in Fig. 3, Fig. 4, Fig. 5, respectively. For each of the figures, a model with good performance, and a model with poor performance were displayed side-to-side (see Fig. 3, Fig. 4, Fig. 5).

## 4 Discussion

In this study, a total of 87 prediction models were externally validated using data from the nationwide NCR and 34 (39%) models showed a good discriminative performance and calibration. On AUC alone, 41 (47%) models showed good performance (AUC ≥0.7), and on the scaled Brier score, 53 (61%) models showed a better performance than an uninformative model. The net benefit of the validated models was assessed using decision curve analysis. It is difficult to provide summary measures of the net benefit for the validated models as the relevant threshold probabilities are necessary to interpret the curve and the thresholds differ between models. Additionally, the threshold probabilities should not be selected based upon the results only displayed in a decision curve, but should be selected based on a clinically reasonable range, combined with the decision curve results [17]. Assessing these ranges was not the aim of the current study, but the provided decision curves can be used as input for future studies elaborating more on the clinical usefulness and impact of implementing one or more of the included models in clinical practice.

To validate the included models, several assumptions had to be made due to the lack of a complete and transparent description of the model in the underlying paper. For instance, the models 18a & 18b developed by Wen et al. predict 5- and 10-year BCSS, respectively, using the log odds of positive lymph nodes as a predictor [18]. The paper provided a definition of this predictor, but did not provide a base value for the logarithmic transformation. Also, Wen et al. [18] presented their model in a nomogram in which the log odds has to be entered as a value between 1 and 4, but no transformation of the predictor was provided. The poor performance of the model may be caused by this lack of transparency and a potentially useful model is not advised to be applied in clinical practice yet. Similar difficulties were identified for the validation of the models 7a-7c provided by Zhao et al. [19] where there were some ambiguous definitions regarding both the predictors and the outcome. For instance, both OS and BCSS were used interchangeably as the outcome, and no proper definitions were provided for variables for which different definitions exist, including oligo-metastasis, breast cancer subtype, or advanced breast. As the cause of death is not available in the NCR, disease-specific mortality was assumed to occur when the patient died while being diagnosed with metastatic disease. The adequate performance found in multiple models predicting BCSS indicates that this assumption was appropriate. Several papers described multiple models that predicted OS and BCSS for metastatic breast cancer patients, such as the models 10a – 10d and 11a – 11l. Due to our definition of BCSS, the dataset used to validate these models was exactly the same (including the OS and BCSS outcomes). Still, differences found in model performance were small and insignificant so we do not expect that this assumption has negatively impacted our results.

The design of the validated models affected the performance measures. For instance, model 23 incorporated LVI as a predictor, where missingness of the predictor was dealt with by modelling “unknown” as a possible input option. However, the coefficient for “unknown” was lower than the other possible input options for the predictor (i.e. LVI or no LVI). As a result, predicted probabilities were lower for all patients compared to a situation in which the predictor values would not be missing, due to the fact that LVI was missing entirely in the NCR. Also, the predictor had no discriminative value this way, as it was equivalent in all patients. Another remarkable finding concerns the models 9d – 9f predicting BCSS over 3, 4, and, 5-year, respectively, where the predicted probability can be higher after 5-years than after 3 or 4 years. It becomes difficult to explain and interpret these results well when applying these models for patient care, regardless of their performance.

The inclusion and exclusion criteria of the original models were applied as much as possible, but some discrepancies were found between the described criteria in the papers describing the development of the models and the group of patients for which the models could be applied. For instance the models 20a and 20b described by Fu et al. [20] include the location of the tumor in the breast as a predictor (e.g. axillary tail, central, lower inner, lower outer, upper inner, or upper outer), but the data in the NCR also include patients with a tumor in an overlapping region. As it was unclear how Fu et al. dealt with these patients, these patients were excluded from the subgroup used for validation of this model, making the results only valid for a smaller group of patients [20].

Although models often predicted the same outcome, these models could barely be compared to each other as their target patient population varied. For instance, model 19 was intended for male breast cancer patients, while others were developed for more general populations. This discrepancy in patient selection criteria may partly account for the variations in model performance. However, poor model performance can also be due to the methodology used to develop and (internally) validate the models. As we previously reported in our systematic review, many prediction models for breast cancer were considered to be at high risk of bias of which Venema et al. [21] demonstrated they perform worse on external validation compared to models with a low risk of bias.

A strength of the current study concerns the large data set used to validate the models. In addition, due to the inclusion of as many identified prognostic models as possible, a total of 87 models could be validated. Given that a total of 922 models were initially considered for external validation, the number of 87 models seems to be low. The majority of the models could not be validated with NCR data due to the unavailability of several required variables such as race, genetic data, LVI, marital status, Ki67, and lymphocytes (including tumor infiltrating lymphocytes and indices such as monocyte-to-lymphocyte ratio). As these data were incorporated in many different models, it is likely to assume that they provide relevant prognostic information and may become valuable additions for future data collection in the NCR or other registries. On the other hand, successful adoption of clinical prediction models relies on both performance and applicability. A model that performs very well, but requires input data that is not routinely collected may be less likely to be widely adopted in clinical practice. The NCR provided a large database with many relevant data items, but some of the commonly missing variables were missing for various reasons. For instance, due to a lack of consistency in definitions of cutoffs and methods to estimate Ki67 [22], the variable is not routinely collected. However, inclusion of predictors such as marital status and race can be considered controversial, and may lead to undesirable effects in addressing disparities [23]. Alternative modelling methods may be applied to improve the applicability of prediction models without losing too much of its predictive performance by e.g. creating submodels in which the users of the models are enabled to still use the model when one or more of the predictors are not available, although estimates will become a little less accurate (reflected in larger confidence intervals) [24].

Multiple models showed a good performance in Dutch breast cancer patients. However, before these models can be used in clinical practice, additional analyses are advised. A potentially useful next step concerns the update and re-calibration of likely valuable models. Subsequent impact studies could further define the value of incorporating some of the validated models in clinical practice. Cost-effectiveness analyses are often omitted, but are perfectly capable of estimating the actual benefits to patients and to the healthcare system when models are used in practice [9]. As highlighted by Vickers et al. a model with good performance does not necessarily indicate a valuable model [17]. In order to assess the value of models, a description of the intended use of the model is required, which should clearly indicate which decision can be supported with the model. For example, a model with a moderate performance may prove valuable if there are no alternatives available, but if there are multiple models with the same intended use, the best performing model on validation should be considered for implementation. Additionally, in the European Union, the use of web-apps to calculate patient-tailored predictions to inform clinical management requires the certification of the software incorporating the model under the medical devices regulation [25]. Developers should take into account the different steps needed to get valuable decision support into clinical practice even before models are developed to improve the efficiency and impact of prediction model development.

## 5 Conclusion

The external validity of 87 prediction models to support treatment decisions of breast cancer patients was assessed. On a large Dutch registry dataset, 34 (39%) models showed a good performance, 26 (30%) models showed a moderate performance, and 27 (31%) models showed a poor performance, according to our predefined definitions. From the models showing good performance, 14 (41%) predicted BCSS, 13 (38%) predicted OS, 3 (9%) predicted OCSS, 2 (6%) predicted metastasis, 1 (3%) predicted DFS, and 1 (1%) predicted LRR. These results allow the next step towards clinical use. After careful evaluation to assess the impact of incorporating the models with a clear intended use in a useable tool, clinical adoption in the Dutch health care setting can be justified.

## Funding source

The study was performed without study sponsors.

## Ethical approval

Ethical approval was not required for this study.

## Data availability

This study used the data from the Netherlands Cancer Registry. Data are available upon request at the Netherlands Comprehensive Cancer Organisation (IKNL) via https://iknl.nl/en/ncr/apply-for-data.
