# Supplementary material for: External validation of 87 clinical prediction models supporting clinical decisions for breast cancer patients
Source: Breast. 2023 Apr 17;69:382–91. doi: 10.1016/j.breast.2023.04.003 (PMC10149388; doi:10.1016/j.breast.2023.04.003)
Supplement: Multimedia component 1 [file mmc1.pdf]

Supplementary materials

Manuscript title: External validation of 87 clinical prediction models supporting clinical treatment decisions for breast cancer patients.

Authors: Tom A. Hueting, Marissa C. van Maaren, Mathijs P. Hendriks, Hendrik Koffijberg, Sabine Siesling.

Table of contents

Model no.	1st Author	Outcome	Page
**1a**	Xiong[1]	Overall survival	3 – 4
**1b**	Xiong[1]	Overall survival	3 – 4
**2**	Regierer[2]	Overall survival	5
**3a**	Fan[3]	Overall survival	6 – 7
**3b**	Fan[3]	Overall survival	6 – 7
**4a**	Luo[4]	Overall survival	8 – 9
**4b**	Luo[4]	Overall survival	8 – 9
**5a**	Zhang[5]	Overall survival	10 – 11
**5b**	Zhang[5]	Overall survival	10 – 11
**5c**	Zhang[5]	Overall survival	10 – 11
**5d**	Zhang[5]	Overall survival	10 – 11
**5e**	Zhang[5]	Breast cancer specific survival	10 – 12
**5f**	Zhang[5]	Breast cancer specific survival	10 – 12
**5g**	Zhang[5]	Breast cancer specific survival	10 – 12
**5h**	Zhang[5]	Breast cancer specific survival	10 – 12
**6a**	Chen[6]	Overall survival	13 – 14
**6b**	Chen[6]	Overall survival	13 – 14
**7a**	Zhao[7]	Overall survival	15 – 16
**7b**	Zhao[7]	Overall survival	15 – 16
**7c**	Zhao[7]	Overall survival	15 – 16
**8a**	Tang[8]	Overall survival	17 - 18
**8b**	Tang[8]	Overall survival	17 - 18
**9a**	Xu[9]	Overall survival	19 – 20
**9b**	Xu[9]	Overall survival	19 – 20
**9c**	Xu[9]	Overall survival	19 – 20
**9d**	Xu[9]	Breast cancer specific survival	19 – 21
**9e**	Xu[9]	Breast cancer specific survival	19 – 21
**9f**	Xu[9]	Breast cancer specific survival	19 – 21
**9g**	Xu[9]	Other cause specific survival	19 – 22
**9h**	Xu[9]	Other cause specific survival	19 – 22
**9i**	Xu[9]	Other cause specific survival	19 – 22
**10a**	Wang[10]	Overall survival	23 – 24
**10b**	Wang[10]	Overall survival	23 – 24
**10c**	Wang[10]	Breast cancer specific survival	23 – 25
**10d**	Wang[10]	Breast cancer specific survival	23 – 25
**11a**	Zheng[11]	Overall survival	26 – 28
**11b**	Zheng[11]	Overall survival	26 – 28
**11c**	Zheng[11]	Overall survival	26 – 28
**11d**	Zheng[11]	Overall survival	26 – 29
**11e**	Zheng[11]	Overall survival	26 – 29
**11f**	Zheng[11]	Overall survival	26 – 29
**11g**	Zheng[11]	Breast cancer specific survival	26 – 30
**11h**	Zheng[11]	Breast cancer specific survival	26 – 30
**11i**	Zheng[11]	Breast cancer specific survival	26 – 30
**11j**	Zheng[11]	Breast cancer specific survival	26 – 31
**11k**	Zheng[11]	Breast cancer specific survival	26 – 31

**11l**	Zheng[11]	Breast cancer specific survival	26 – 31
**12a**	Janssen[12]	Overall survival	32 – 33
**12b**	Janssen[12]	Overall survival	32 – 33
**13a**	Wang[13]	Overall survival	34 – 35
**13b**	Wang[13]	Overall survival	34 – 35
**14**	Abdel-Rahman[14]	Breast cancer specific survival	36
**15**	Elwood[15]	Breast cancer specific survival	37 – 38
**16a**	Paredes Aracil[16]	Breast cancer specific survival	39 – 40
**16b**	Paredes Aracil[16]	Breast cancer specific survival	39 – 40
**17a**	Wen[17]	Breast cancer specific survival	41 – 42
**17b**	Wen[17]	Breast cancer specific survival	41 – 42
**18a**	Wen[18]	Breast cancer specific survival	43 – 44
**18b**	Wen[18]	Breast cancer specific survival	43 – 44
**19a**	Chen[19]	Breast cancer specific survival	45 – 46
**19b**	Chen[19]	Breast cancer specific survival	45 – 46
**20a**	Fu[20]	Breast cancer specific survival	47 – 48
**20b**	Fu[20]	Breast cancer specific survival	47 – 48
**21**	Herrero Vicent[21]	Locoregional recurrence	49
**22**	Wobb[22]	Locoregional recurrence	50
**23**	Sanghani[23]	Local recurrence	51
**24**	Li[24]	Locoregional recurrence	52
**25a**	Corso[25]	Local recurrence	53 – 54
**25b**	Corso[25]	Local recurrence	53 – 54
**25c**	Corso[25]	Local recurrence	53 – 54
**26**	Li[26]	Recurrence	55
**27**	Tokatli[27]	Disease free survival	56
**28a**	Lin[28]	Disease free survival	57 – 58
**28b**	Lin[28]	Disease free survival	57 – 58
**28c**	Lin[28]	Disease free survival	57 – 58
**29a**	Paredes Aracil[29]	Recurrence	59 – 60
**29b**	Paredes Aracil[29]	Recurrence	59 – 60
**30**	Dowsett[30]	Metastasis	61
**31**	Lin[31]	Metastasis	62
**32a**	Lim[32]	Metastasis	63 – 64
**32b**	Lim[32]	Metastasis	63 – 64
**33**	Boutros[33]	Metastasis	65
**34**	Zhang[34]	Lymph node involvement	66
**35**	Meretoja[35]	Lymph node involvement	67
**36**	Houvanaeghel[36]	Lymph node involvement	68
**37**	Schipper[37]	Pathologic complete response	69
**38**	Pan[38]	Positive surgical margin	70

Figure captions

To save space and avoid unnecessary repetition of the same text at each of the figures, the figure captions have been left out of the supplementary data. The figures (calibration plot, classification plot, and decision curve) are explained in the main article (figures 3 – 5)

1. Xiong et al. (Models 1a & 1b)

Reference: *Xiong, Z., Deng, G., Huang, X., Li, X., Xie, X., Wang, J., ... & Wang, X. (2018). Score for the survival probability in metastasis breast cancer: a nomogram-based risk assessment model. Cancer research and treatment: official journal of Korean Cancer Association, 50(4), 1260.*

Number of models presented in the paper: 2

Outcome:	1-year overall survival<br>3-year overall survival
Input variables:	Age (<35, 35-50, >50)<br>MFI (Primary stage IV, <6 months, 6 months - <2 year, 2 – 5 year, >5 years)<br>Metastasis site (bone/soft tissue, visceral/CNS, multiple)<br>Hormonal receptor status (Negative, positive)
Inclusion criteria:	Pathologic diagnosis of breast cancer, Diagnosis of metastatic breast cancer, age <70
Exclusion criteria:	Unclear date of MBC diagnosis, no follow-up, previous cancer.
Original validation:	0.67 (95% CI: 0.63 – 0.71) on external cohort

Variable name	Input	1-year OS<br>N (%) total = 11633	3-year OS<br>N (%) total = 10964
Age	<35	424 (3.6%)	390 (3.6%)
	35-50	4359 (37.5%)	4105 (37.4%)
	>50	6850 (58.9%)	6469 (59%)
Metastatic free interval	Primary stage	6686 (57.5%)	6019 (54.9%)
	<6 months	130 (1.1%)	130 (1.2%)
	6 months - <2 years	1678 (14.4%)	1676 (15.3%)
	2-5 years	2444 (21%)	2444 (22.3%)
	>5 years	694 (6%)	694 (6.3%)
Metastatic site	Bone/soft tissue	3469 (29.8%)	3153 (28.8%)
	Visceral/CNS	1836 (15.8%)	1741 (15.9%)
	Multiple	6328 (54.4%)	6070 (55.4%)
Hormone receptor status	Negative	2909 (25%)	2779 (25.3%)
	Positive	8060 (69.3%)	7522 (68.6%)
	Missing	664 (5.7%)	663 (6%)
Overall survival status	Deceased	3294 (28.3%)	6820 (62.2%)
	Survived	8339 (71.7%)	4144 (37.8%)

Overall survival	1-year	3-year
AUC	0.668 (0.656 – 0.678)	0.652 (0.642 – 0.661)
Brier score	0.210 (0.204 – 0.216)	0.233 (0.231 – 0.234)
Scaled brier score	-0.033 (-0.043 – -0.024)	0.010 (-0.003 – 0.025)

1-year overall survival (model 1a)

3-year overall survival (model 1b)

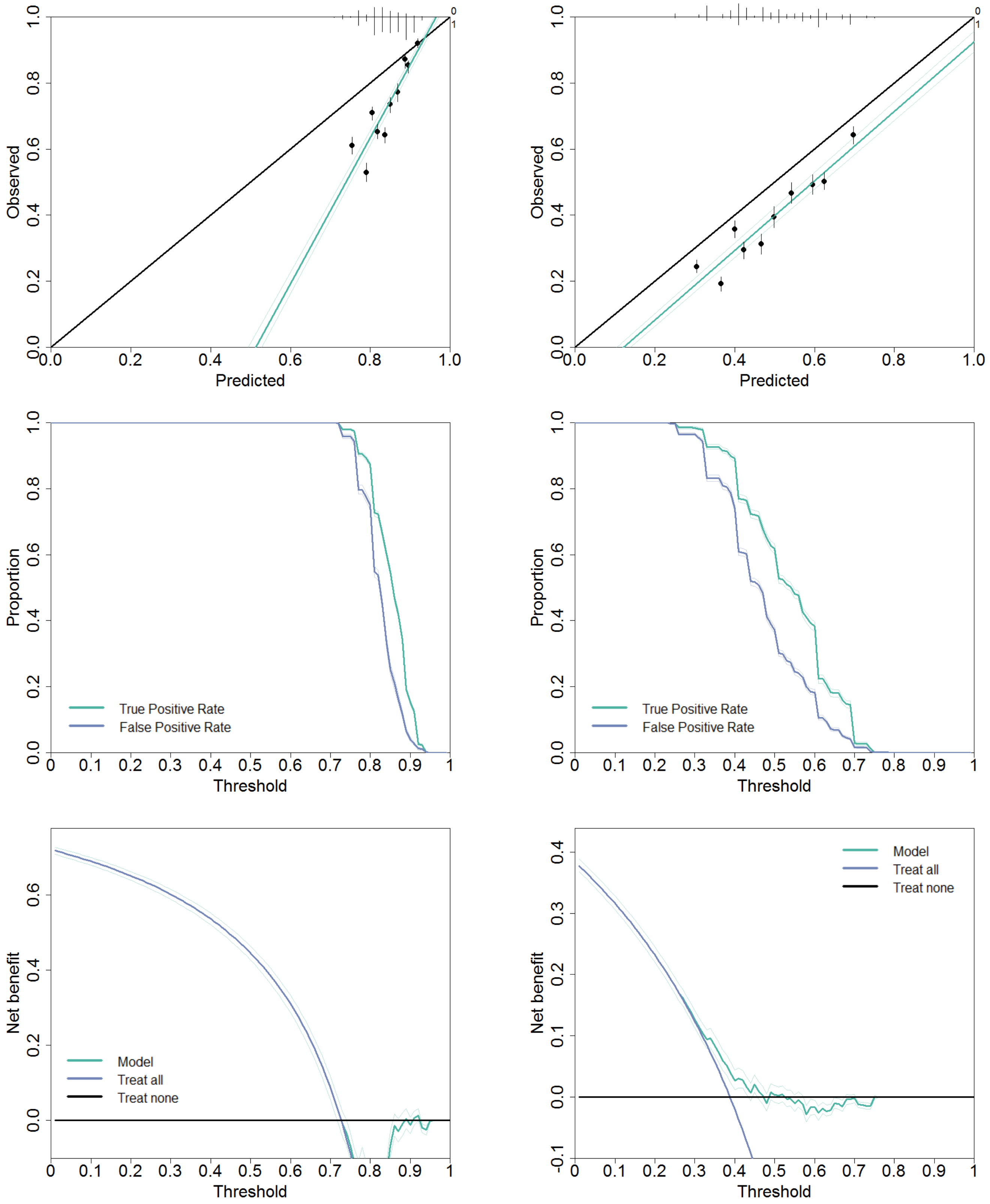

2. Regierer et al. (Model 2)

Reference: Regierer, A. C., Wolters, R., Ufen, M. P., Weigel, A., Novopashenny, I., Köhne, C. H., ... & Wischnewsky, M. B. (2014). An internally and externally validated prognostic score for metastatic breast cancer: analysis of 2269 patients. *Annals of oncology*, *25*(3), 633-638.

Number of models presented in the paper: 1

Outcome:	5-year overall survival
Input variables:	Metastasis free survival (≤2 years, >2 years)<br>Hormone receptor status (Negative, Positive)<br>Metastatic site (Liver, Effusion, Brain, Bone, Bone marrow, Soft tissue, Lung, Other)
Inclusion criteria:	Metastatic breast cancer
Exclusion criteria:	NA
Original validation:	0.686

Variable name	Input	N (%) total = 17608
Metastasis free interval	≤2 years	16670 (94.7%)
	>2 years	938 (5.3%)
Hormone receptor status	Negative	12044 (68.4%)
	Positive	4186 (23.8%)
	Missing	1378 (7.8%)
Metastatic site	Liver	5042 (28.6%)
	Effusion	1492 (8.5%)
	Brain	881 (5%)
	Bone	10297 (58.5%)
	Bone marrow	183 (1%)
	Soft tissue	283 (1.6%)
	Lung	3670 (20.8%)
	Other	2075 (11.8%)
5-year overall survival	No	13403 (76.1%)
	Yes	4205 (23.9%)

AUC	0.622 (0.614 – 0.631)
Brier score	0.194 (0.191 – 0.196)
Scaled Brier score	-0.065 (-0.079 – -0.050)

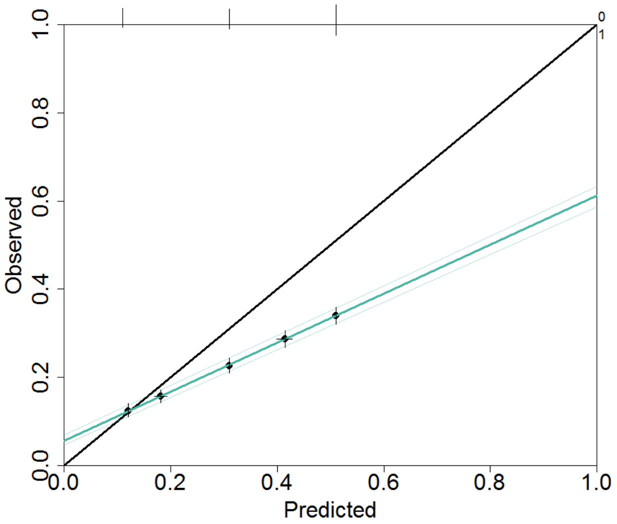

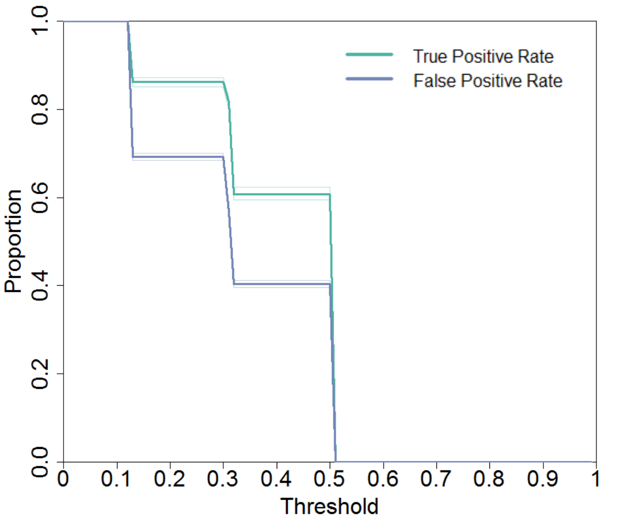

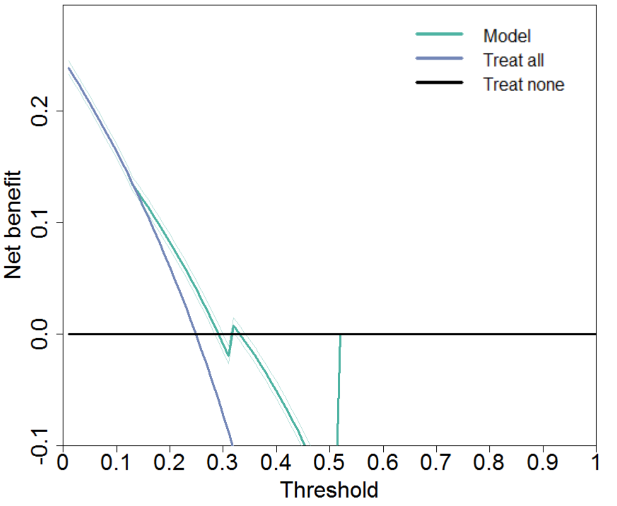

3. Fan et al. (Models 3a & 3b)

Reference: FAN, Y. P., LIU, C. L., CHIANG, I. J., & LIN, C. Y. (2011). Development of a prognostic nomogram for identifying those factors which influence the 2-and 5-year survival chances of Taiwanese women diagnosed with breast cancer. *European journal of cancer care*, *20*(5), 620-626.

Number of models presented in the paper:

Outcome:	2-year overall survival<br>5-year overall survival
Input variables:	Age (continuous)<br>Tumor stage (T1, T2, T3, T4)<br>Nodal stage (N0, N1, N2, N3)<br>Metastatic stage (M0, M1)<br>ER status (Negative, Positive)
Inclusion criteria:	Women with invasive breast cancer who underwent mastectomy
Exclusion criteria:	NA
Original validation:	0.80

Variable name	Input	2-year OS<br>N (%) total = 86418	5-year OS<br>N (%) total = 73465
Age	Years (mean (sd))	61 (15)	61 (15)
Tumor stage	T0	2620 (3%)	1648 (2.2%)
	T1	39746 (46%)	33357 (45.4%)
	T2	35928 (41.6%)	31513 (42.9%)
	T3	6446 (7.5%)	5428 (7.4%)
	T4	1678 (1.9%)	1519 (2.1%)
Nodal stage	N0	45548 (52.7%)	37779 (51.4%)
	N1	26811 (31%)	22654 (30.8%)
	N2	8556 (9.9%)	7852 (10.7%)
	N3	5503 (6.4%)	5180 (7.1%)
Metastatic stage	M0	84649 (98%)	71919 (97.9%)
	M1	1769 (2%)	1546 (2.1%)
ER-status	Negative	16365 (18.9%)	14332 (19.5%)
	Positive	67360 (77.9%)	56541 (77%)
	Missing	2693 (3.1%)	2592 (3.5%)
Overall survival	No	5609 (6.5%)	15321 (20.9%)
	Yes	80809 (93.5%)	58144 (79.1%)

	2-year OS (3a)	5-year OS (3b)
AUC	0.665 (0.658 – 0.673)	0.683 (0.678 – 0.687)
Brier score	0.068 (0.067 – 0.070)	0.167 (0.165 – 0.169)
Scaled Brier score	-0.123 (-0.137 – -0.108)	-0.013 (-0.024 – -0.004)

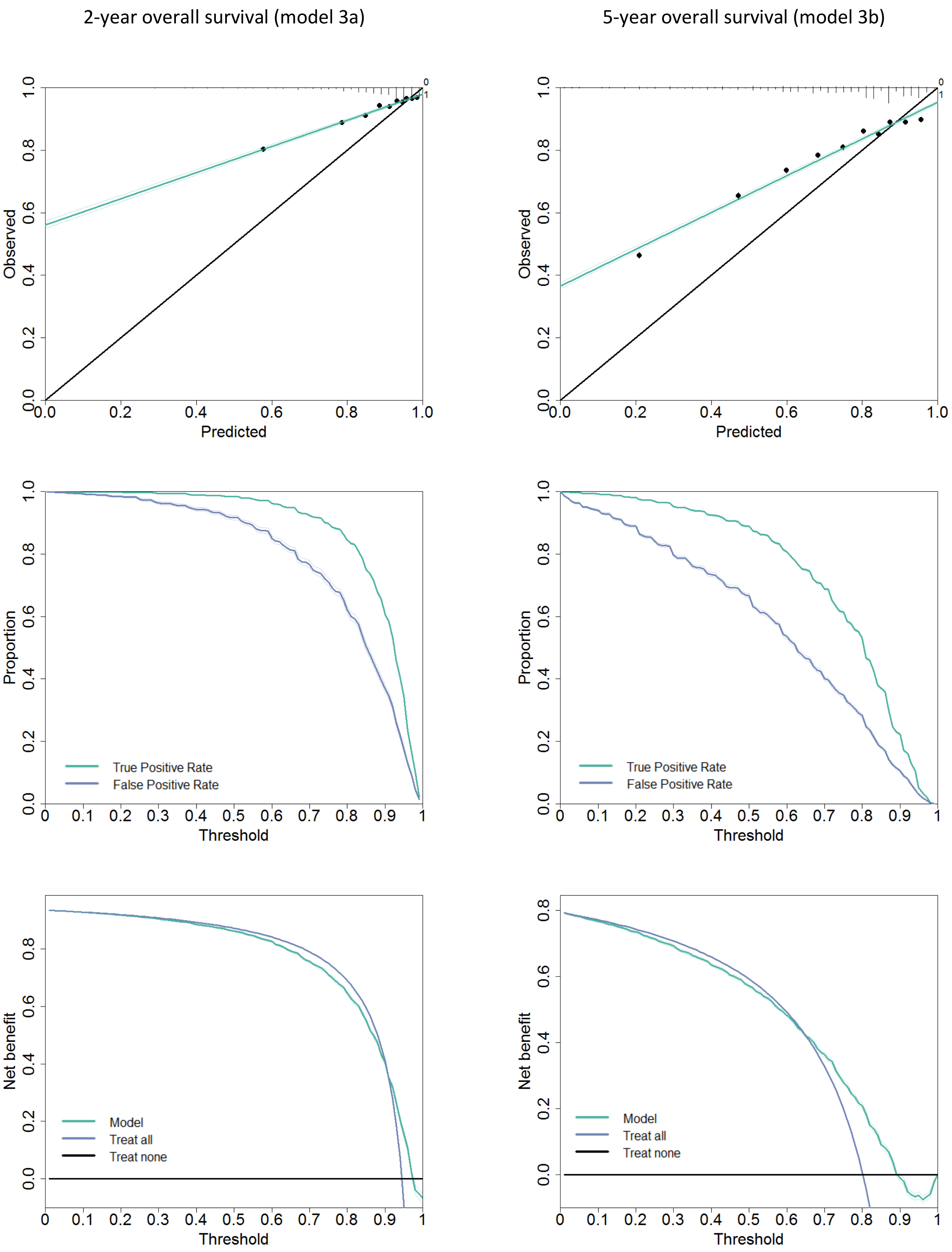
2-year overall survival (model 3a)
5-year overall survival (model 3b)
Observed
Predicted
0.0
0.2
0.4
0.6
0.8
1.0
Proportion
True Positive Rate
False Positive Rate
Threshold
0
0.1
0.2
0.3
0.4
0.5
0.6
0.7
0.8
0.9
1
Net benefit
Model
Treat all
Treat none

4. Luo et al. (Models 4a & 4b)

Reference: Luo, C., Zhong, X., Wang, Z., Wang, Y., Wang, Y., He, P., ... & Zheng, H. (2019). Prognostic nomogram for patients with non-metastatic HER2 positive breast cancer in a prospective cohort. *The International journal of biological markers*, *34*(1), 41-46.

Number of models presented in the paper: 2

Outcome:	3-year overall survival<br>5-year overall survival
Input variables:	Age (continuous)<br>ER status (Positive, Negative)<br>T stage (T1, T2, T3, T4)<br>N stage (N0, N1, N2, N3)<br>Trastuzumab (No, Yes)
Inclusion criteria:	Non-metastatic, HER2+, female, underwent mastectomy or lumpectomy
Exclusion criteria:	Neoadjuvant therapy, previous history of malignancies
Original validation:	0.78 (95% CI: 0.72 – 0.83) & 0.74 (95% CI: 0.72 – 0.76) in training and validation cohorts, respectively.

Variable name	Input	3-year OS<br>N (%) total = 15107	5-year OS<br>N (%) total = 13599
Age	Years (mean (sd))	57 (13)	57 (13)
ER status	Negative	5231 (34.6%)	4788 (35.2%)
	Positive	9783 (64.8%)	8720 (64.1%)
	Missing	93 (0.6%)	91 (0.7%)
T-stage	T1	8876 (58.8%)	7814 (57.5%)
	T2	5662 (37.5%)	5254 (38.6%)
	T3	435 (2.9%)	409 (3%)
	T4	134 (0.9%)	122 (0.9%)
N-stage	N0	8865 (58.7%)	7771 (57.1%)
	N1	4153 (27.5%)	3817 (28.1%)
	N2	1281 (8.5%)	1230 (9%)
	N3	808 (5.3%)	781 (5.7%)
Trastuzumab	No	5548 (36.7%)	5115 (37.6%)
	Yes	9559 (63.3%)	8484 (62.4%)
Overall survival	No	981 (6.5%)	1688 (12.4%)
	Yes	14126 (93.5%)	11911 (87.6%)

	3-year OS (4a)	5-year OS (4b)
AUC	0.619 (0.598 – 0.636)	0.597 (0.583 – 0.610)
Brier score	0.062 (0.059 – 0.067)	0.114 (0.110 – 0.119)
Scaled Brier score	-0.005 (-0.013 – 0.005)	-0.029 (-0.038 – -0.018)

3-year Overall survival (model 4a)

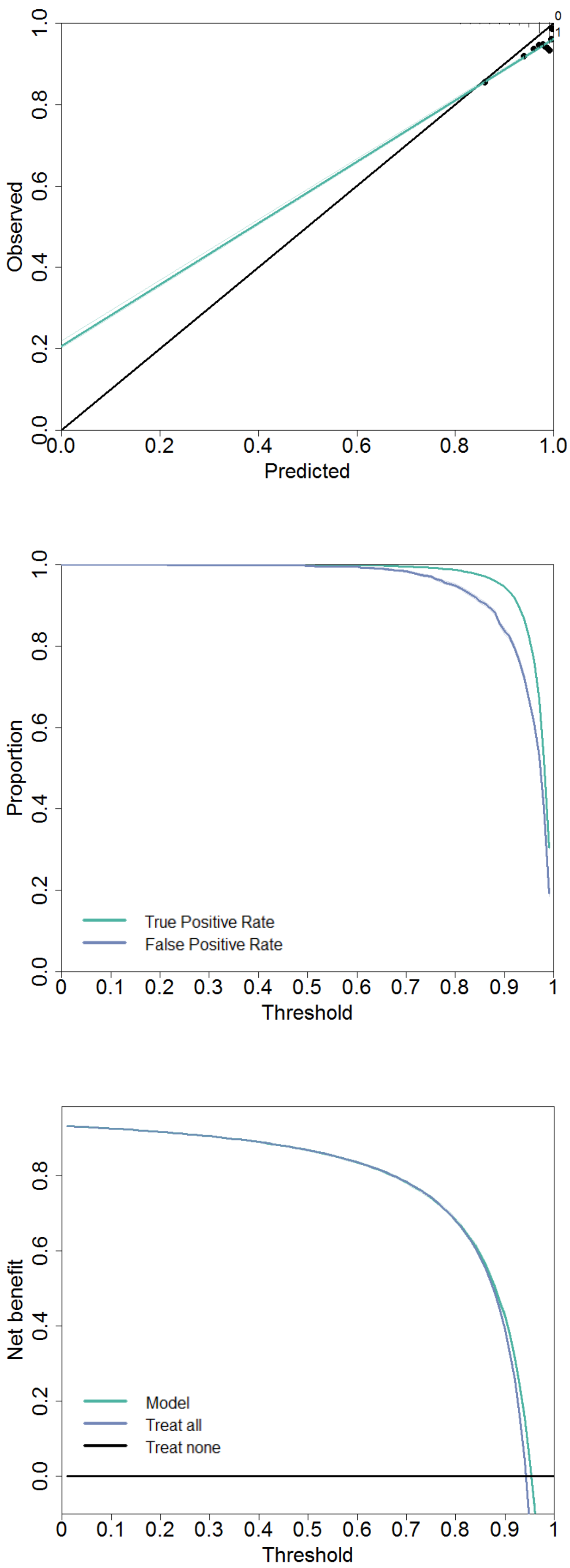

5-year Overall survival (model 4b)

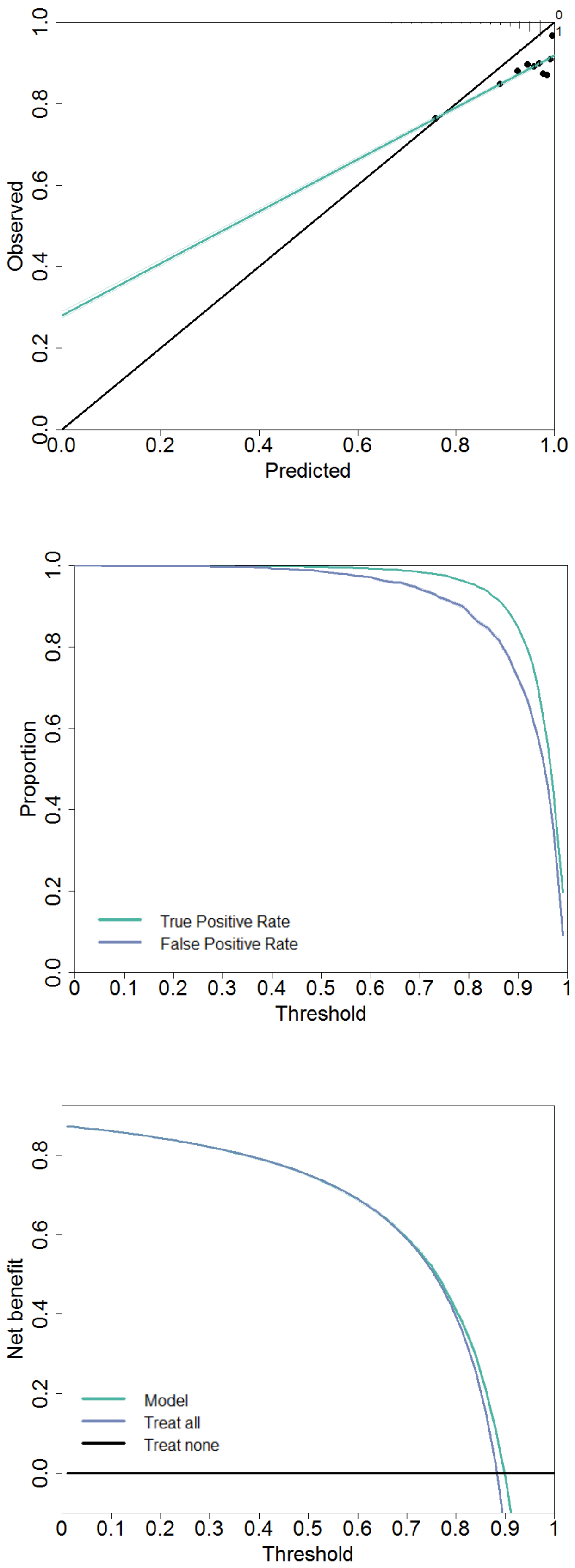

5. Zhang et al. (Models 5a – 5h)

Reference: Zhang, N., Zhang, J., Zhang, H., Liu, Y., Zhao, W., Wang, L., ... & Yang, Q. (2019). Individualized prediction of survival benefit from postmastectomy radiotherapy for patients with breast cancer with one to three positive axillary lymph nodes. *The oncologist*, *24*(12), e1286.

Number of models presented in the paper: 8 models/outcomes in total

Outcome:	5 & 10 year disease specific survival (with & without PMRT)<br>5 & 10 year overall survival (with & without PMRT)
Input variables:	Age (18 – 40, 40 – 50, 50 – 60 , 60 – 70, 70 – 80)<br>Tumor grade (I, II, III)<br>T stage (1, 2, 3)<br>Involved LN (1, 2, 3)<br>ER status (Negative, Positive)<br>PR status (Negative, Positive)
Inclusion criteria:	Female patients, 18 – 80 years, invasive breast cancer
Exclusion criteria:	Histopathology subtypes other than invasive lobular or invasive ductal carcinoma. Lymph node negative patients or >3 positive lymph nodes. < 8 lymph nodes removed. Bilateral breast cancer, metastasis. No axillary dissection, T4 tumors or unknown tumor size, Diagnosed from autopsy or death certificate.
Original validation:	OS & PMRT: 0.687 and 0.672 on internal and external, respectively.<br>OS & no PMRT: 0.700 and 0.696 on internal and external, respectively.<br>DSS & PMRT: 0.699 and 0.656 on internal and external, respectively.<br>DSS & no PMRT: 0.716 and 0.671 on internal and external, respectively.

Variable name	Input	PMRT 5-year N = 3208	PMRT 10-year N = 2072	No PMRT 5-year N = 10423	No PMRT 10-year N = 8254
Age	18 – 40	408 (12.7%)	270 (13%)	759 (7.3%)	631 (7.6%)
	40 – 50	1044 (32.5%)	638 (30.8%)	2503 (24%)	1950 (23.6%)
	50 – 60	805 (25.1%)	523 (25.2%)	2896 (27.8%)	2272 (27.5%)
	60 – 70	576 (18%)	350 (16.9%)	2403 (23.1%)	1865 (22.6%)
	70 – 80	375 (11.7%)	291 (14%)	1862 (17.9%)	1536 (18.6%)
Tumor grade	I	251 (7.8%)	158 (7.6%)	1568 (15%)	1174 (14.2%)
	II	1142 (35.6%)	713 (34.4%)	4745 (45.5%)	3657 (44.3%)
	III	1177 (36.7%)	804 (38.8%)	3420 (32.8%)	2876 (34.8%)
	Missing	638 (19.9%)	394 (19%)	690 (6.6%)	547 (6.6%)
T stage	T1	990 (30.9%)	623 (30.1%)	4607 (44.2%)	3565 (43.2%)
	T2	1589 (49.5%)	1057 (51%)	5459 (52.4%)	4389 (53.2%)
	T3	629 (19.6%)	392 (18.9%)	357 (3.4%)	300 (3.6%)
Lymph nodes	1	1335 (41.6%)	857 (41.4%)	6033 (57.9%)	4734 (57.4%)
	2	977 (30.5%)	622 (30%)	2914 (28%)	2317 (28.1%)
	3	896 (27.9%)	593 (28.6%)	1476 (14.2%)	1203 (14.6%)
ER status	Negative	662 (20.6%)	471 (22.7%)	1638 (15.7%)	1385 (16.8%)
	Positive	2521 (78.6%)	1582 (76.4%)	8456 (81.1%)	6552 (79.4%)
	Missing	25 (0.8%)	19 (0.9%)	329 (3.2%)	317 (3.8%)
PR status	Negative	1109 (34.6%)	764 (36.9%)	3139 (30.1%)	2588 (31.4%)
	Positive	2031 (63.3%)	1248 (60.2%)	6694 (64.2%)	5093 (61.7%)
	Missing	68 (2.1%)	60 (2.9%)	590 (5.7%)	573 (6.9%)
Overall survival	No	509 (15.9%)	828 (40%)	1317 (12.6%)	2560 (31%)
	Yes	2699 (84.1%)	1244 (60%)	9106 (87.4%)	5694 (69%)
Breast cancer specific survival	No	123 (3.8%)	189 (9.1%)	377 (3.6%)	564 (6.8%)
	Yes	2699 (84.1%)	1244 (60%)	9106 (87.4%)	5694 (69%)
	Cause unclear	386 (12%)	639 (30.8%)	940 (9%)	1996 (24.2%)

Overall survival

5-year Radio (5a) 10-year Radio (5b) 5-year No radio (5c) 10-year no radio (5d)

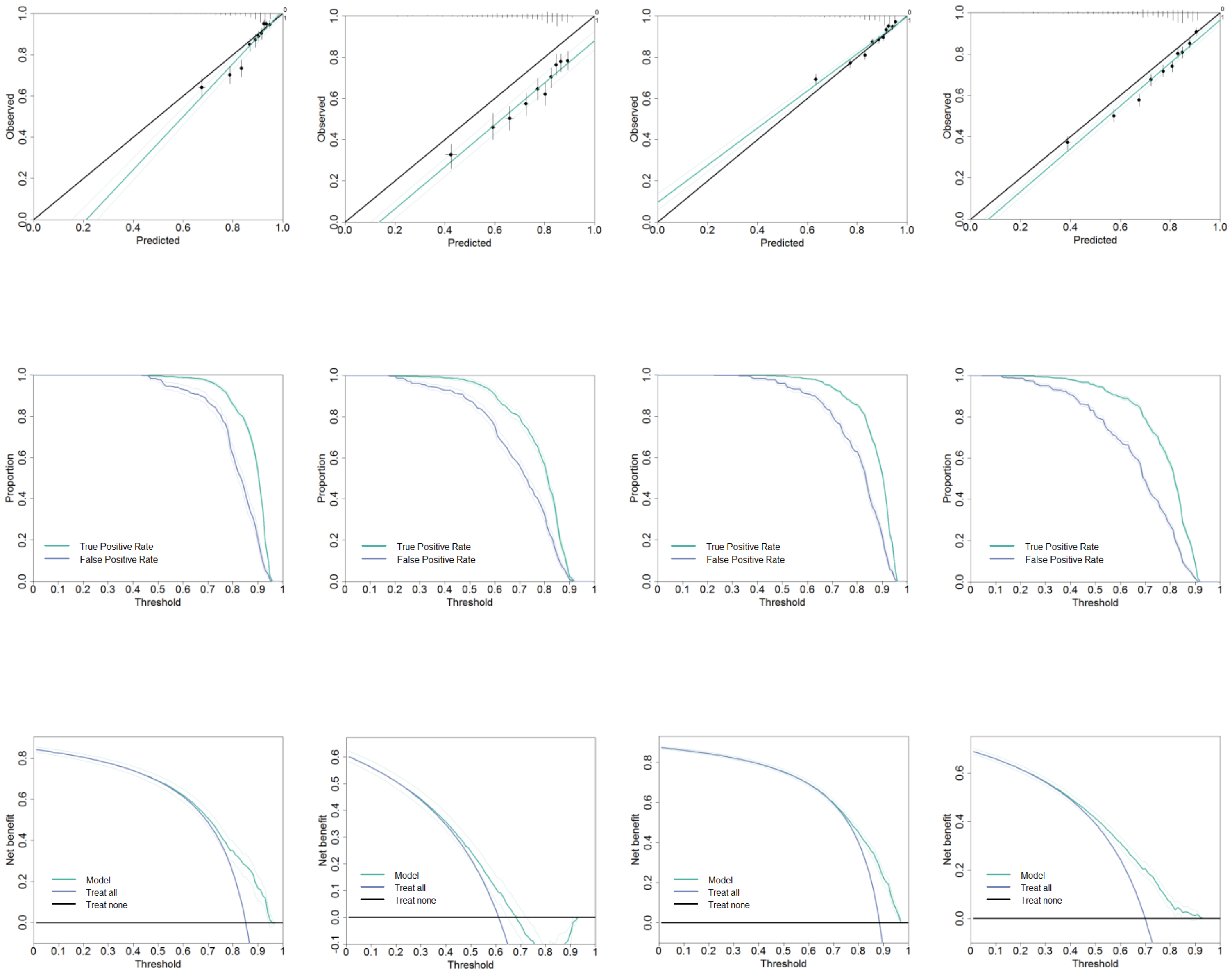

	5-year Radio (5a)	10-year Radio (5b)	5-year no radio (5c)	10-year no radio (5d)
AUC	0.726 (0.703 – 0.747)	0.672 (0.650 – 0.699)	0.715 (0.702 – 0.731)	0.711 (0.700 – 0.723)
Brier score	0.123 (0.115 – 0.133)	0.238 (0.226 – 0.248)	0.103 (0.099 – 0.107)	0.192 (0.186 – 0.196)
Scaled Brier score	0.078 (0.059 – 0.096)	0.008 (-0.023 – 0.043)	0.067 (0.054 – 0.080)	0.106 (0.092 – 0.118)

Breast cancer specific survival

5-year Radio (5e)	10-year Radio (5f)	5-year No radio (5g)	10-year no radio (5h)

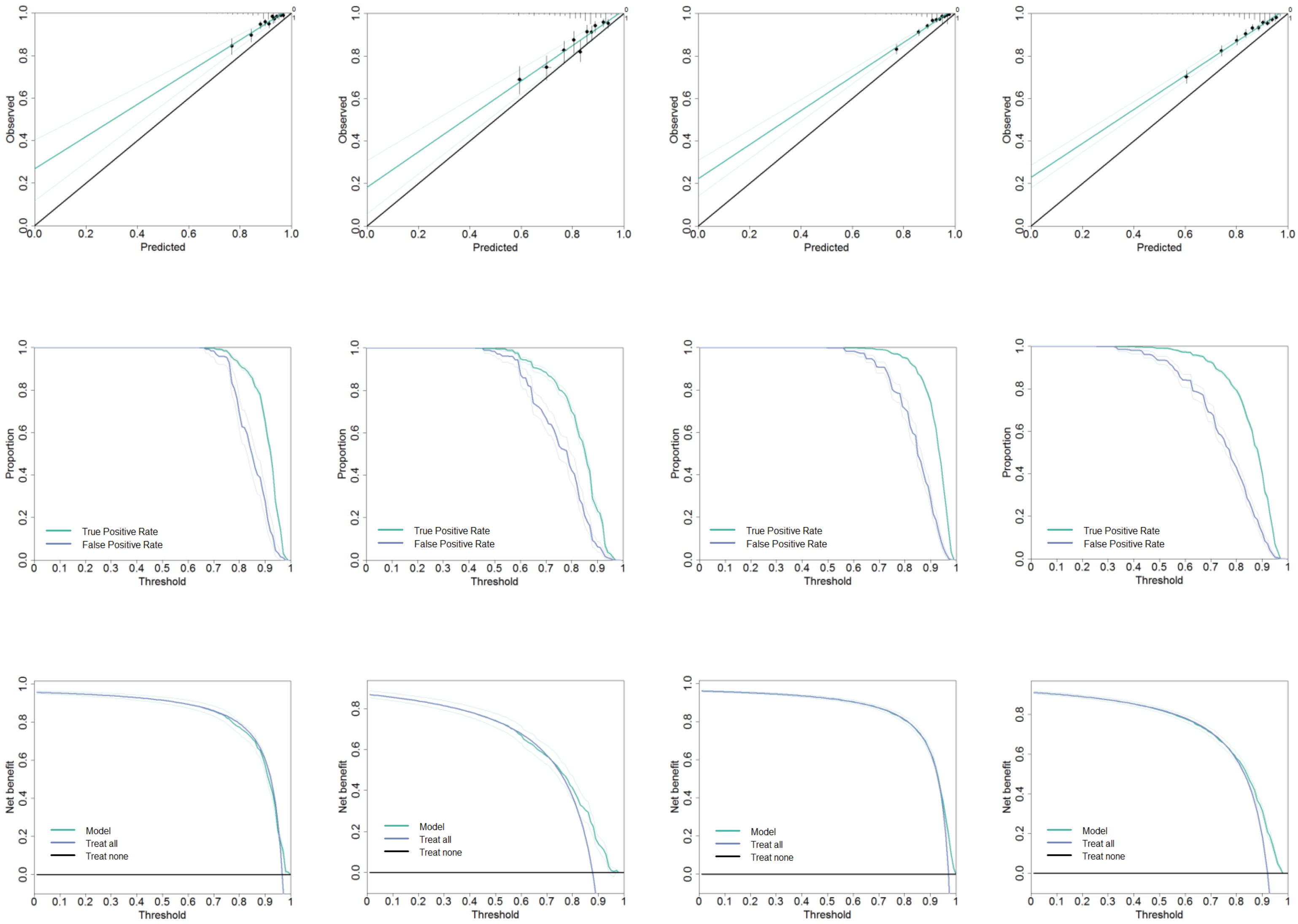

	5-year Radio (5e)	10-year Radio (5f)	5-year no radio (5g)	10-year no radio (5h)
AUC	0.758 (0.716 – 0.799)	0.702 (0.667 – 0.735)	0.801 (0.780 – 0.820)	0.751 (0.731 – 0.772)
Brier score	0.043 (0.037 – 0.049)	0.111 (0.100 – 0.120)	0.038 (0.035 – 0.041)	0.080 (0.075 – 0.084)
Scaled Brier score	-0.039 (-0.102 – 0.005)	0.033 (-0.011 – 0.069)	0.006 (-0.027 – 0.032)	0.023 (-0.007 – 0.051)

6. Chen et al. (Models 6a & 6b)

Reference: Chen, Y. C., Lai, H. W., Wang, W. C., & Kuo, Y. L. (2016). Validation of breast Cancer survival prediction model with SEER database. *J Integr Oncol*, *5*(3), 174.

Number of models presented in the paper: 2

Outcome:	5-year overall survival
Input variables:	Age (continuous)<br>Grade (I, II, III, IV)<br>T stage (T0-T1, T2, T3-T4)<br>N stage (N0, N1, N2, N3)<br>Hormone receptor status (Negative, Positive)
Inclusion criteria:	Breast cancer diagnosis
Exclusion criteria:	Follow-up < 1 year, Metastatic disease, ductal carcinoma in situ
Original validation:	Model 1 (SEER): 0.822 and 0.78 in SEER and NCKUH data, respectively<br>Model 2 (NCKUH): 0.792 and 0.80 in SEER and NCKUH data, respectively

Variable name	Input	N (%) total = 170643
Age	Years (mean (sd))	60 (13)
Grade	I	37035 (21.7%)
	II	70999 (41.6%)
	III	46945 (27.5%)
	IV	22 (0%)
	Missing	15642 (9.2%)
T stage	T0	5581 (3.3%)
	T1	105136 (61.6%)
	T2	52219 (30.6%)
	T3	5537 (3.2%)
	T4	1685 (1%)
N stage	N0	110398 (64.7%)
	N1	43456 (25.5%)
	N2	10494 (6.1%)
	N3	6105 (3.6%)
Hormone receptor status	Negative	27099 (15.9%)
	Positive	137288 (80.5%)
	Missing	6256 (3.7%)
5-year overall survival	No	24021 (14.1%)
	Yes	146622 (85.9%)

	SEER-model (6a)	NCKUH-model (6b)
AUC	0.696 (0.692 – 0.700)	0.622 (0.618 – 0.626)
Brier score	0.115 (0.114 – 0.116)	0.120 (0.119 – 0.121)
Scaled Brier score	0.047 (0.041 – 0.052)	0.007 (0.003 – 0.010)

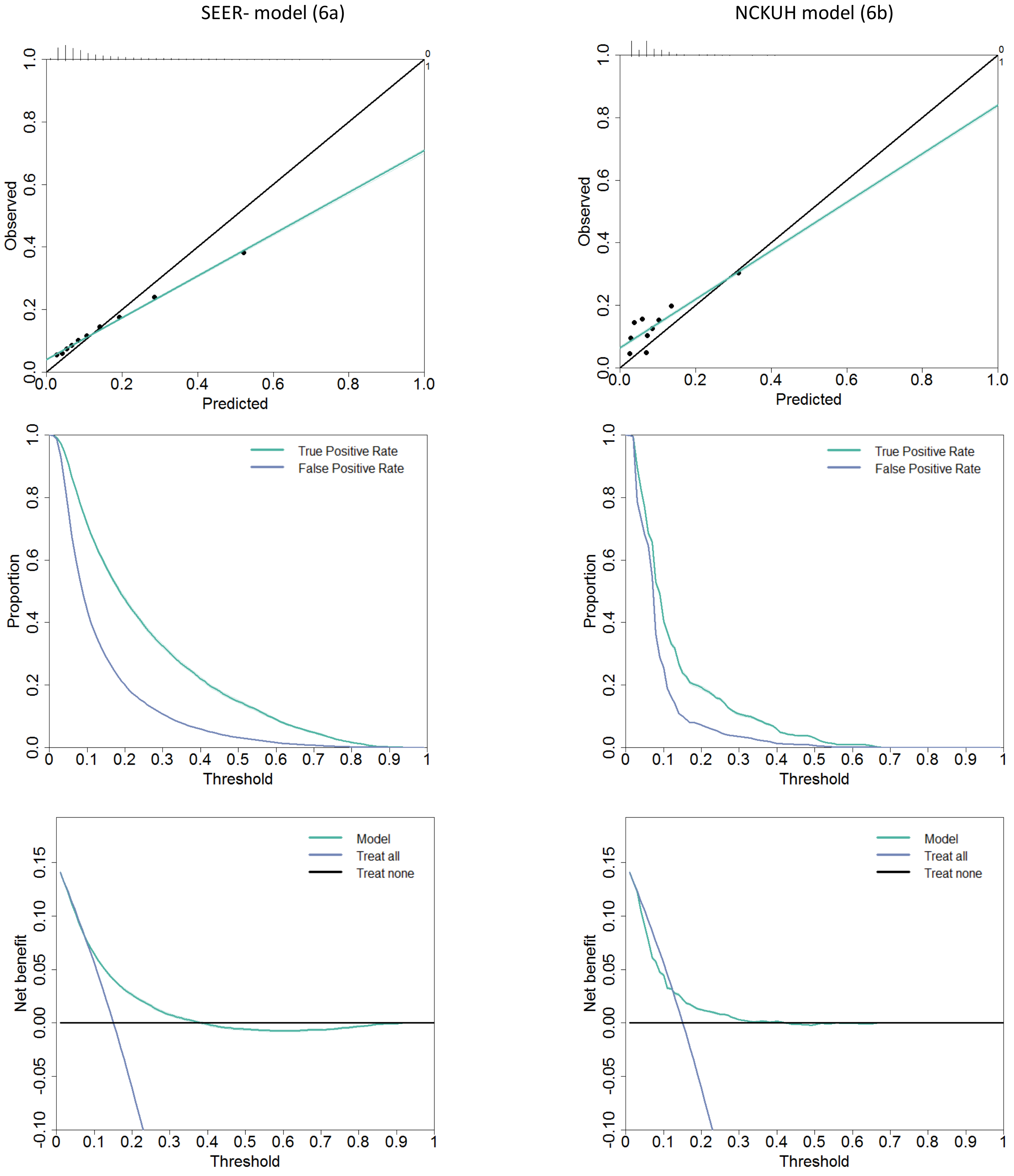

SEER- model (6a)
NCKUH model (6b)
Observed
Predicted
Proportion
Threshold
True Positive Rate
False Positive Rate
Net benefit
Model
Treat all
Treat none

7. Zhao et al. (Models 7a, 7b, 7c)

Reference: Zhao, J., Yang, Y., Pang, D., Yu, Y., Lin, X., Chen, K., ... & Wang, Y. (2020). Development and validation of a nomogram in survival prediction among advanced breast cancer patients. *Annals of Translational Medicine*, *8*(21).

Number of models presented in the paper: 3

Outcome:	1-year overall survival<br>2-year overall survival<br>3-year overall survival
Input variables:	Tumor stage (I/II, III/IV)<br>Molecular subtype (HR+/HER2-, HER2+, TNBC)<br>Disease free survival (<24m, 24 – 48m, >48m)<br>Tumor burden (Recurrence, Oligo-metastasis, Multi-metastasis)<br>Brain metastasis (No, Yes)
Inclusion criteria:	Female patients with advanced breast cancer, 18 years and older, follow-up time at least 24 months.
Exclusion criteria:	Prior malignancy
Original validation:	0.77 (95% CI: 0.71 – 0.84) and 0.71 (95% CI: 0.60 – 0.82) for derivation and validation cohorts, respectively.

Variable name	Input	Total N = 8745
Tumor stage	I	2027 (23.2%)
	II	3570 (40.8%)
	III	3145 (36%)
	IV	3 (0%)
Molecular subtype	HR+/HER2-	3155 (36.1%)
	HER2+	1230 (14.1%)
	TNBC	1273 (14.6%)
	Missing	3087 (35.3%)
Disease free survival	<24 months	3300 (37.7%)
	24 – 48 months	3158 (36.1%)
	>48 months	2287 (26.2%)
Tumor burden	Recurrence	1964 (22.5%)
	Oligo-metastasis*	6710 (76.7%)
	Multi-metastasis	71 (0.8%)
Brain metastasis	No	8547 (97.7%)
	Yes	198 (2.3%)
Survival status 1-year	Deceased	3174 (36.3%)
	Survived	5571 (63.7%)
Survival status 2-year	Deceased	4760 (54.4%)
	Survived	3983 (45.5%)
	Lost to follow-up	2 (0%)
Survival status 3-year	Deceased	5796 (66.3%)
	Survived	2944 (33.7%)
	Lost to follow-up	5 (0.1%)

* Oligo-metastasis was defined as having a maximum of 5 metastatic sites.

	1-year	2-year	3-year
AUC	0.731 (0.720 – 0.741)	0.75 (0.74 – 0.76)	0.776 (0.765 – 0.787)
Brier score	0.332 (0.323 – 0.343)	0.382 (0.374 – 0.389)	0.356 (0.350 – 0.361)
Scaled Brier score	-0.437 (-0.463 – -0.415)	-0.541 (-0.574 – -0.503)	-0.593 (-0.635 – -0.547)

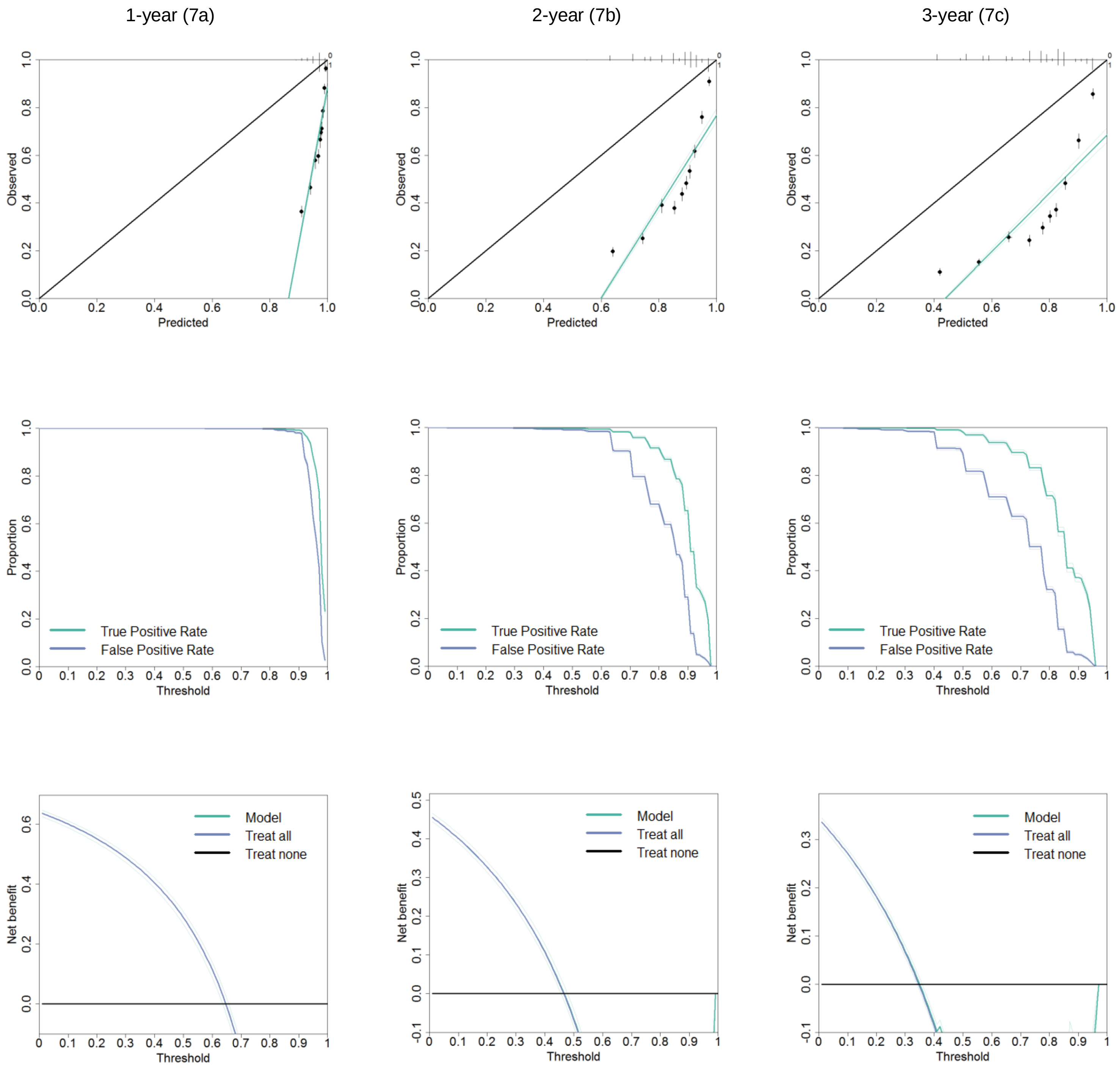
1-year (7a)
2-year (7b)
3-year (7c)
Observed
Predicted
Proportion
Threshold
True Positive Rate
False Positive Rate
Net benefit
Model
Treat all
Treat none

8. Tang et al. (Models 8a & 8b)

Reference: Tang, Y., Zhang, Y. J., Zhang, N., Shi, M., Wen, G., Cheng, J., ... & Li, Y. X. (2020). Nomogram predicting survival as a selection criterion for postmastectomy radiotherapy in patients with T1 to T2 breast cancer with 1 to 3 positive lymph nodes. *Cancer*, *126*, 3857-3866.
Number of models presented in the paper: 2

Outcome:	5-year overall survival<br>10-year overall survival
Input variables:	Age (≤40, >40)<br>Tumor location (Other, Inner)<br>Tumor size (continuous)<br>Lymph node ratio (≤10%, 10% - 20%, >20%)<br>ER status (negative, positive)<br>PR status (negative, positive)<br>HER2 status (negative, positive & trastuzumab, positive & no trastuzumab)
Inclusion criteria:	Pathologically confirmed invasive breast cancer, treated with mastectomy and ALND, pT1-2N1M0 disease
Exclusion criteria:	Neoadjuvant therapy, history of other cancers, bilateral or occult breast cancer, follow-up <6 months
Original validation:	0.70 (95% CI: 0.67 – 0.74)

Variable name	Input	5-year OS<br>N (%) total = 8774	10-year OS<br>N (%) total = 7238
Age	≤40	781 (8.9%)	631 (8.7%)
	>40	7993 (91.1%)	6607 (91.3%)
Tumor location	Inner	1266 (14.4%)	1042 (14.4%)
	Other	7508 (85.6%)	6196 (85.6%)
Tumor size	mm (median (IQR))	23 (17 – 30)	23 (17 – 30)
Lymph node ratio	≤10%	4452 (50.7%)	3597 (49.7%)
	10 – 20%	2769 (31.6%)	2297 (31.7%)
	>20%	1553 (17.7%)	1344 (18.6%)
ER status	Negative	1636 (18.6%)	1396 (19.3%)
	Positive	6965 (79.4%)	5674 (78.4%)
	Missing	173 (2%)	168 (2.3%)
PR status	Negative	2876 (32.8%)	2431 (33.6%)
	Positive	5483 (62.5%)	4399 (60.8%)
	Missing	415 (4.7%)	408 (5.6%)
HER2 status	Negative	5701 (65%)	4498 (62.1%)
	Positive & trastuzumab	892 (10.2%)	616 (8.5%)
	Positive & no trastuzumab	414 (4.7%)	379 (5.2%)
	Missing	1767 (20.1%)	1745 (24.1%)
OS	No	1566 (17.8%)	2745 (37.9%)
	Yes	7208 (82.2%)	4493 (62.1%)

	5-year OS (8a)	10-year OS (8b)
AUC	0.650 (0.638 – 0.663)	0.604 (0.591 – 0.618)
Brier score	0.166 (0.157 – 0.173)	0.322 (0.314 – 0.332)
Scaled Brier score	-0.129 (-0.138 – -0.116)	-0.369 (-0.396 – -0.346)

5-year Overall Survival (8a)

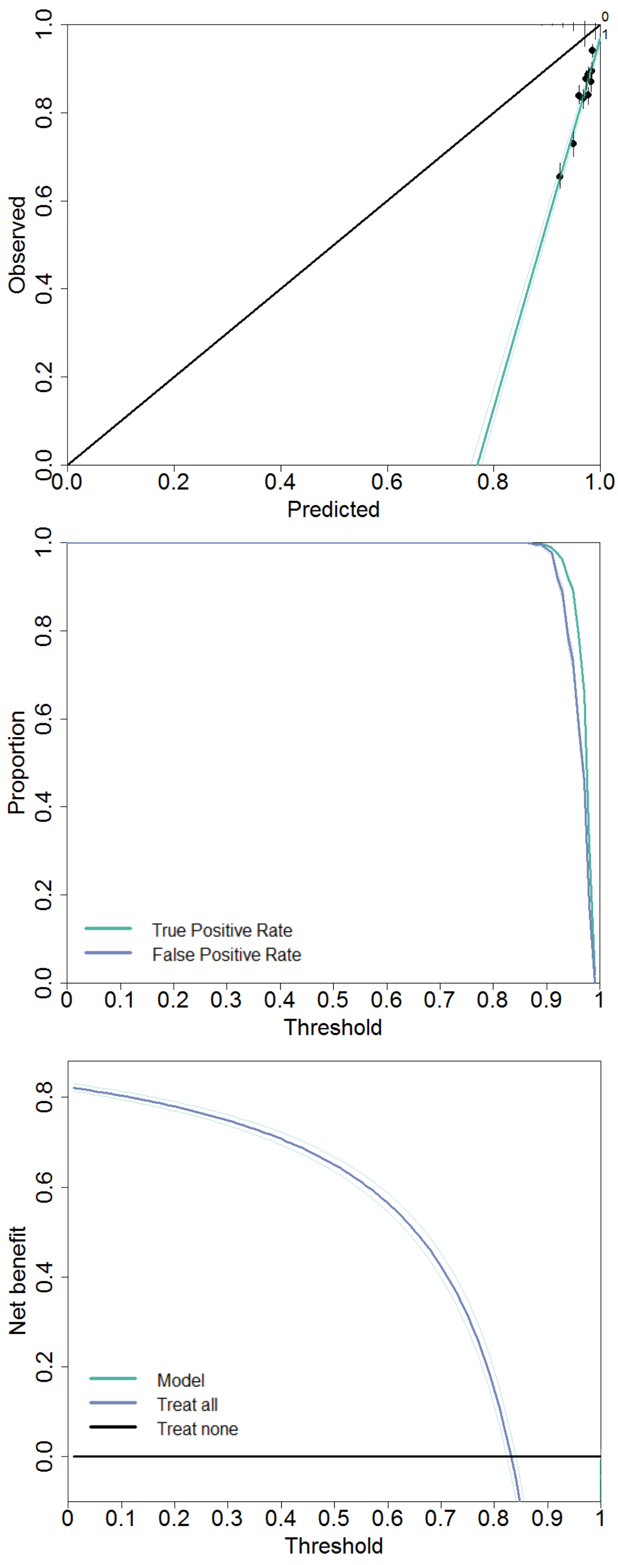

10-year Overall survival (8b)

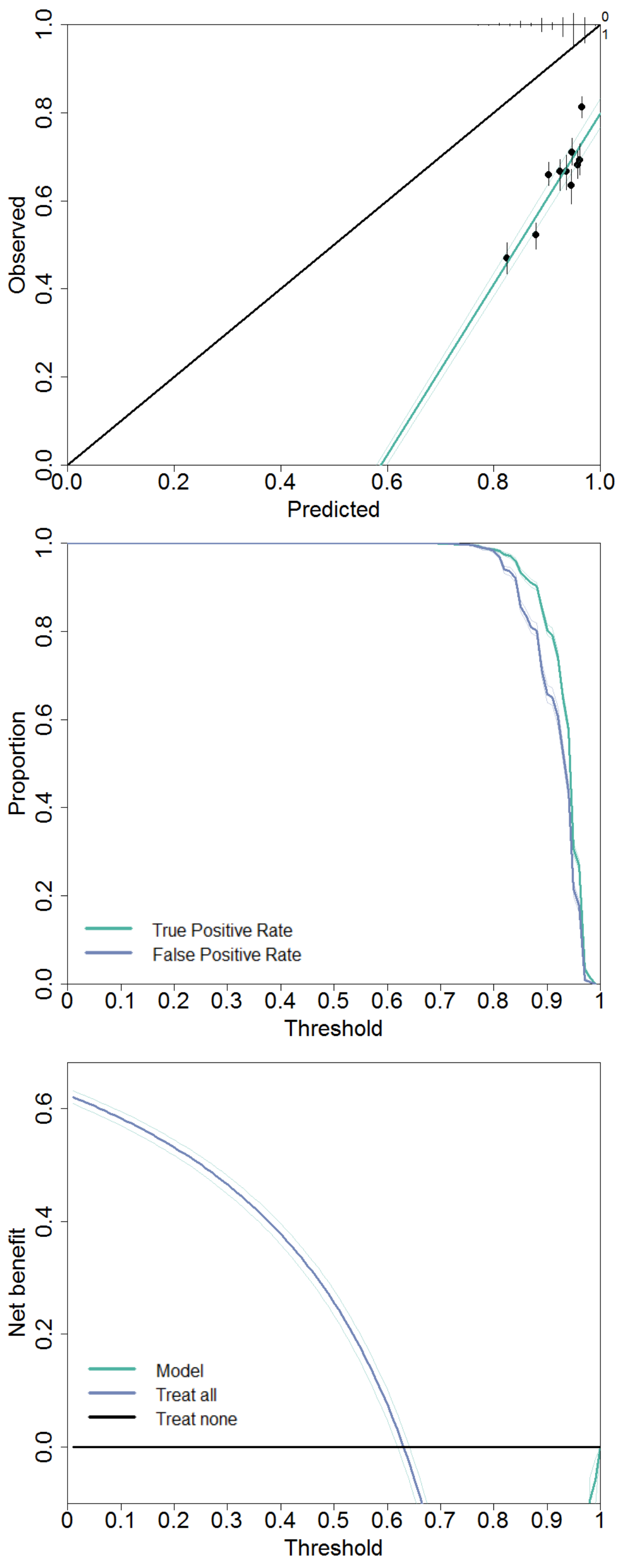

9. Xu et al. (Models 9a – 9i)

Reference: Xu, Y. B., Liu, H., Cao, Q. H., Ji, J. L., Dong, R. R., & Xu, D. (2020). Evaluating overall survival and competing risks of survival in patients with early-stage breast cancer using a comprehensive nomogram. *Cancer medicine*, *9*(12), 4095-4106.
Number of models presented in the paper: 9

Outcome:	3-year Overall, breast cancer specific, and other cause specific survival<br>4-year Overall, breast cancer specific, and other cause specific survival<br>5-year Overall, breast cancer specific, and other cause specific survival
Input variables:	Age (years) (<60, 60 – 65, 66 – 70, 71 – 75, 76 – 80, 81 – 85, >85)<br>Grade (I, II, III/IV)<br>Tumor size (cm) (<1, 1.0 – 1.9, 2.0 – 2.9, 3.0 – 3.9, ≥4.0)<br>Molecular subtype (Lum A, Lum B, HER2+, Triple negative)<br>Surgical treatment (no, yes)
Inclusion criteria:	Stage I-II breast cancer
Exclusion criteria:	Stage III-IV, other malignancies, diagnosis at autopsy, incomplete survival time, Age <18 years, survival time <1 month.
Original validation:	3-year: 0.802, 0.830, and 0.813 for OS, BCSS, and OCSS, respectively<br>4-year: 0.795, 0.817 and 0.808 for OS, BCSS, and OCSS, respectively<br>5-year: 0.787, 0.803, and 0.817 for OS, BCSS, and OCSS, respectively

Variable name	Input	3-year OS N (%) total = 175927	4-year OS N (%) total = 161550	5-year OS N (%) total = 147892	3-year BCSS N (%) total = 168847	4-year BCSS N (%) total = 151702	5-year BCSS N (%) total = 135451
Age	<60	83439 (47.4%)	76860 (47.6%)	70454 (47.6%)	81923 (48.5%)	74665 (49.2%)	67622 (49.9%)
	60 – 65	28914 (16.4%)	26583 (16.5%)	24246 (16.4%)	28119 (16.7%)	25463 (16.8%)	22791 (16.8%)
	66 – 70	23919 (13.6%)	21580 (13.4%)	19343 (13.1%)	23017 (13.6%)	20302 (13.4%)	17702 (13.1%)
	71 – 75	19033 (10.8%)	17184 (10.6%)	15588 (10.5%)	18033 (10.7%)	15803 (10.4%)	13840 (10.2%)
	76 – 80	9373 (5.3%)	8671 (5.4%)	8125 (5.5%)	8391 (5%)	7330 (4.8%)	6443 (4.8%)
	81 – 85	7732 (4.4%)	7296 (4.5%)	6886 (4.7%)	6662 (3.9%)	5843 (3.9%)	5076 (3.7%)
	>85	3517 (2%)	3376 (2.1%)	3250 (2.2%)	2702 (1.6%)	2296 (1.5%)	1977 (1.5%)
Grade	I	40190 (22.8%)	36815 (22.8%)	33575 (22.7%)	39108 (23.2%)	35286 (23.3%)	31585 (23.3%)
	II	77289 (43.9%)	70365 (43.6%)	63914 (43.2%)	74649 (44.2%)	66570 (43.9%)	58930 (43.5%)
	III	49502 (28.1%)	45898 (28.4%)	42438 (28.7%)	46473 (27.5%)	41841 (27.6%)	37567 (27.7%)
	IV	18 (0%)	18 (0%)	18 (0%)	17 (0%)	17 (0%)	16 (0%)
	Missing	8928 (5.1%)	8454 (5.2%)	7947 (5.4%)	8600 (5.1%)	7988 (5.3%)	7353 (5.4%)
Tumor size	<1.0	35508 (20.2%)	31352 (19.4%)	27584 (18.7%)	34692 (20.5%)	30194 (19.9%)	26109 (19.3%)
	1.0 – 1.9	77040 (43.8%)	71081 (44%)	65290 (44.1%)	74786 (44.3%)	67846 (44.7%)	61078 (45.1%)
	2.0 – 2.9	38287 (21.8%)	35852 (22.2%)	33459 (22.6%)	36350 (21.5%)	33144 (21.8%)	30023 (22.2%)
	3.0 – 3.9	13675 (7.8%)	12821 (7.9%)	12032 (8.1%)	12667 (7.5%)	11432 (7.5%)	10314 (7.6%)
	≥4.0	11418 (6.5%)	10444 (6.5%)	9527 (6.4%)	10352 (6.1%)	9086 (6%)	7927 (5.9%)
Molecular subtype	Luminal A	112979 (64.2%)	103270 (63.9%)	94086 (63.6%)	108908 (64.5%)	97425 (64.2%)	86406 (63.8%)
	Luminal B	12249 (7%)	11252 (7%)	10337 (7%)	11826 (7%)	10651 (7%)	9548 (7%)
	HER2 +	5974 (3.4%)	5547 (3.4%)	5178 (3.5%)	5607 (3.3%)	5070 (3.3%)	4607 (3.4%)
	TNBC	16151 (9.2%)	15021 (9.3%)	13972 (9.4%)	14542 (8.6%)	12962 (8.5%)	11637 (8.6%)
	Missing	28574 (16.2%)	26460 (16.4%)	24319 (16.4%)	27964 (16.6%)	25594 (16.9%)	23253 (17.2%)
Surgery	No	55 (0%)	54 (0%)	42 (0%)	54 (0%)	52 (0%)	40 (0%)
	Yes	175872 (100%)	161496 (100%)	147850 (100%)	168793 (100%)	151650 (100%)	135411 (100%)
OS	No	10609 (6%)	14904 (9.2%)	18983 (12.8%)	NA	NA	NA
	Yes	165318 (94%)	146646 (90.8%)	128909 (87.2%)	NA	NA	NA
BCSS	No	NA	NA	NA	1586 (0.9%)	2319 (1.5%)	2973 (2.2%)
	Yes	NA	NA	NA	167261 (99.1%)	149383 (98.5%)	132478 (97.8%)
OCSS	No	NA	NA	NA	9023 (5.3%)	12585 (8.3%)	16010 (11.8%)
	Yes	NA	NA	NA	166904 (98.8%)	148965 (98.2%)	131882 (97.4%)

Overall survival

3 year (9a) | 4-year (9b) | 5-year (9c)

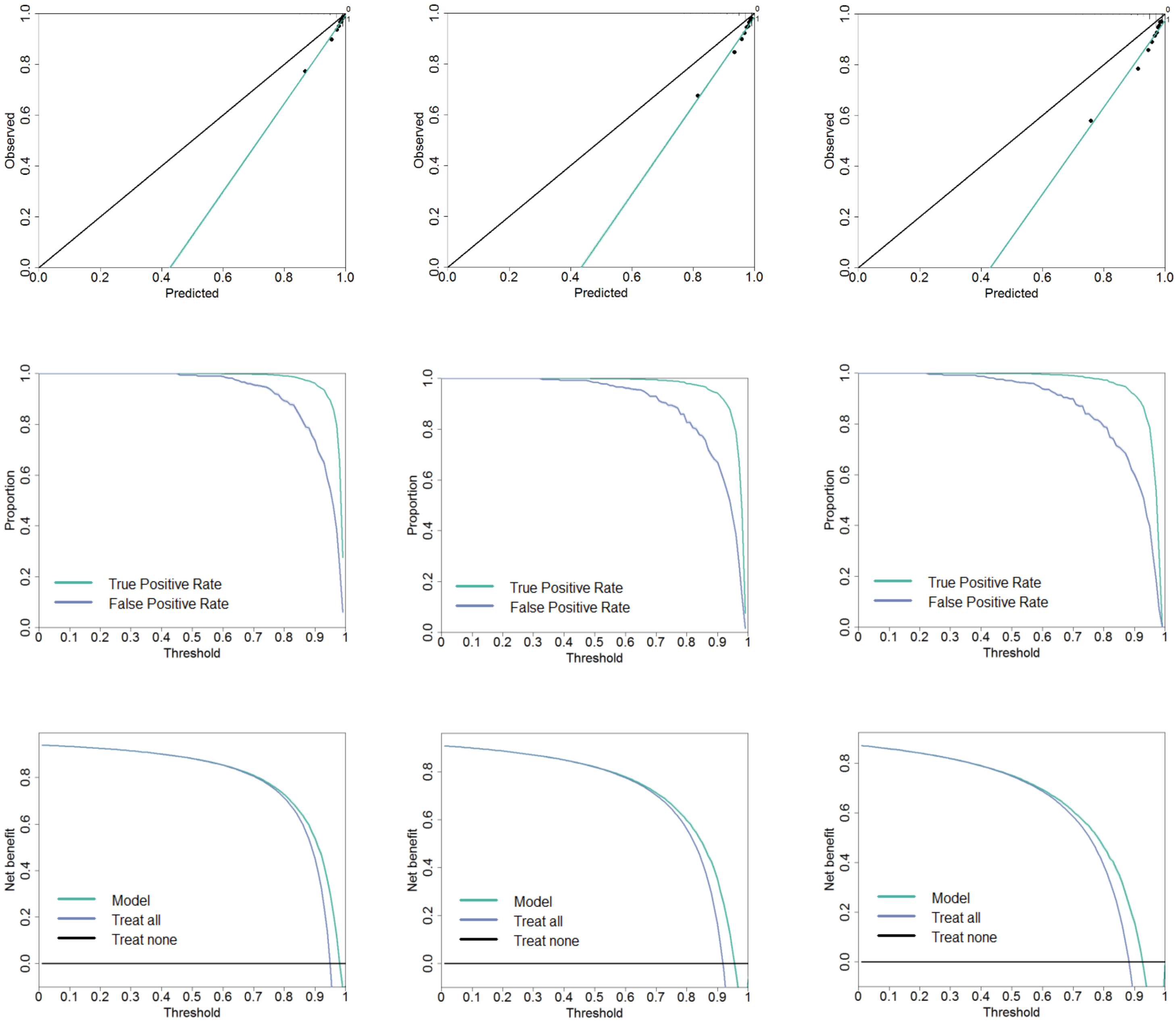

	3-year (9a)	4-year (9b)	5-year (9c)
AUC	0.775 (0.770 – 0.779)	0.769 (0.766 – 0.774)	0.763 (0.760 – 0.767)
Brier score	0.053 (0.052 – 0.054)	0.078 (0.077 – 0.079)	0.104 (0.103 – 0.106)
Scaled Brier score	0.060 (0.057 – 0.063)	0.067 (0.064 – 0.071)	0.067 (0.063 – 0.070)

Breast cancer specific survival

3 year (9d) | 4-year (9e) | 5-year (9f)

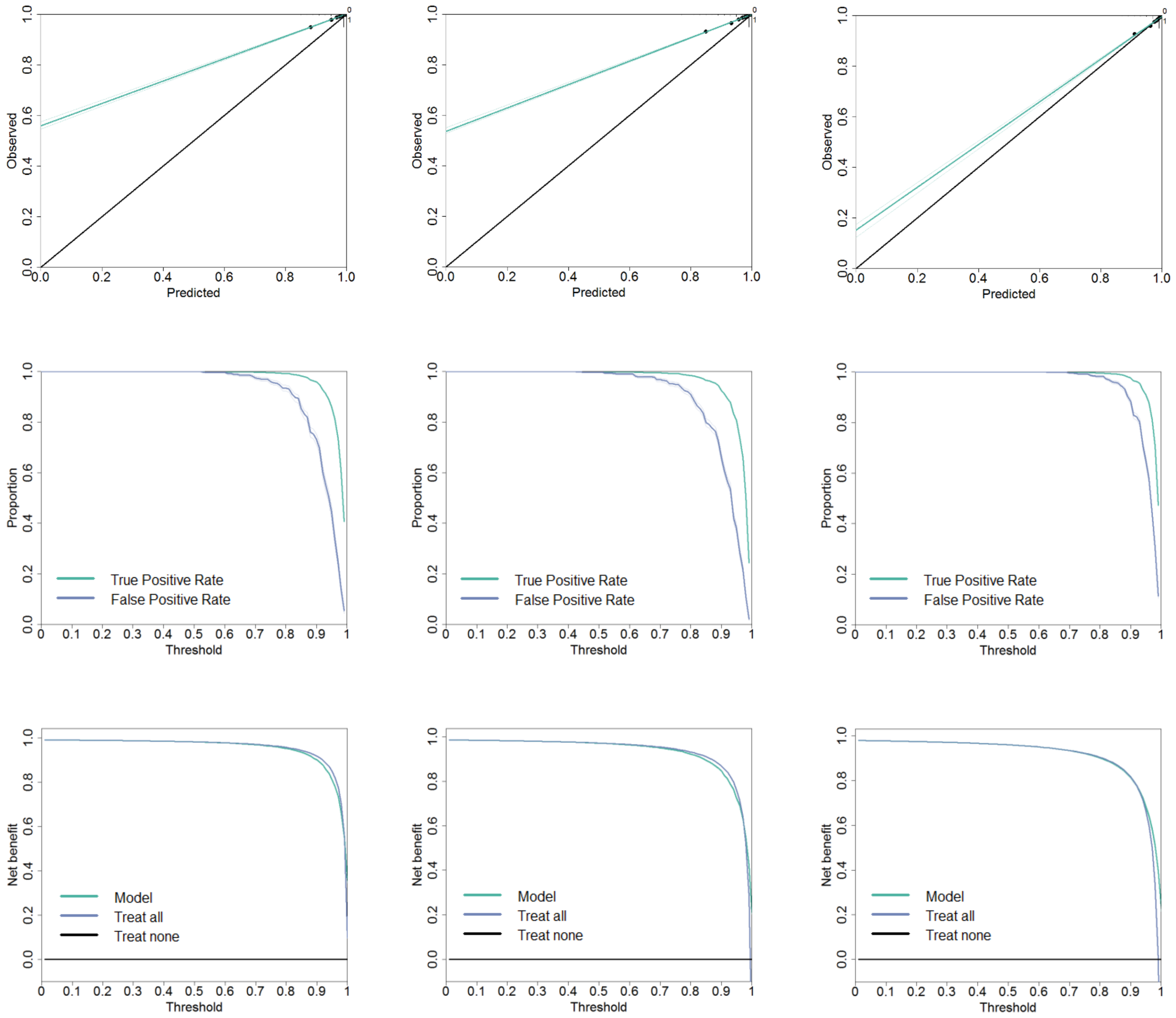

	3-year (9d)	4-year (9e)	5-year (9f)
AUC	0.818 (0.808 – 0.828)	0.796 (0.788 – 0.803)	0.774 (0.766 – 0.781)
Brier score	0.010 (0.009 – 0.010)	0.015 (0.015 – 0.016)	0.019 (0.019 – 0.020)
Scaled Brier score	-0.092 (-0.105 – -0.079)	-0.086 (-0.099 – -0.077)	0.014 (0.011 – 0.018)

Other cause specific survival

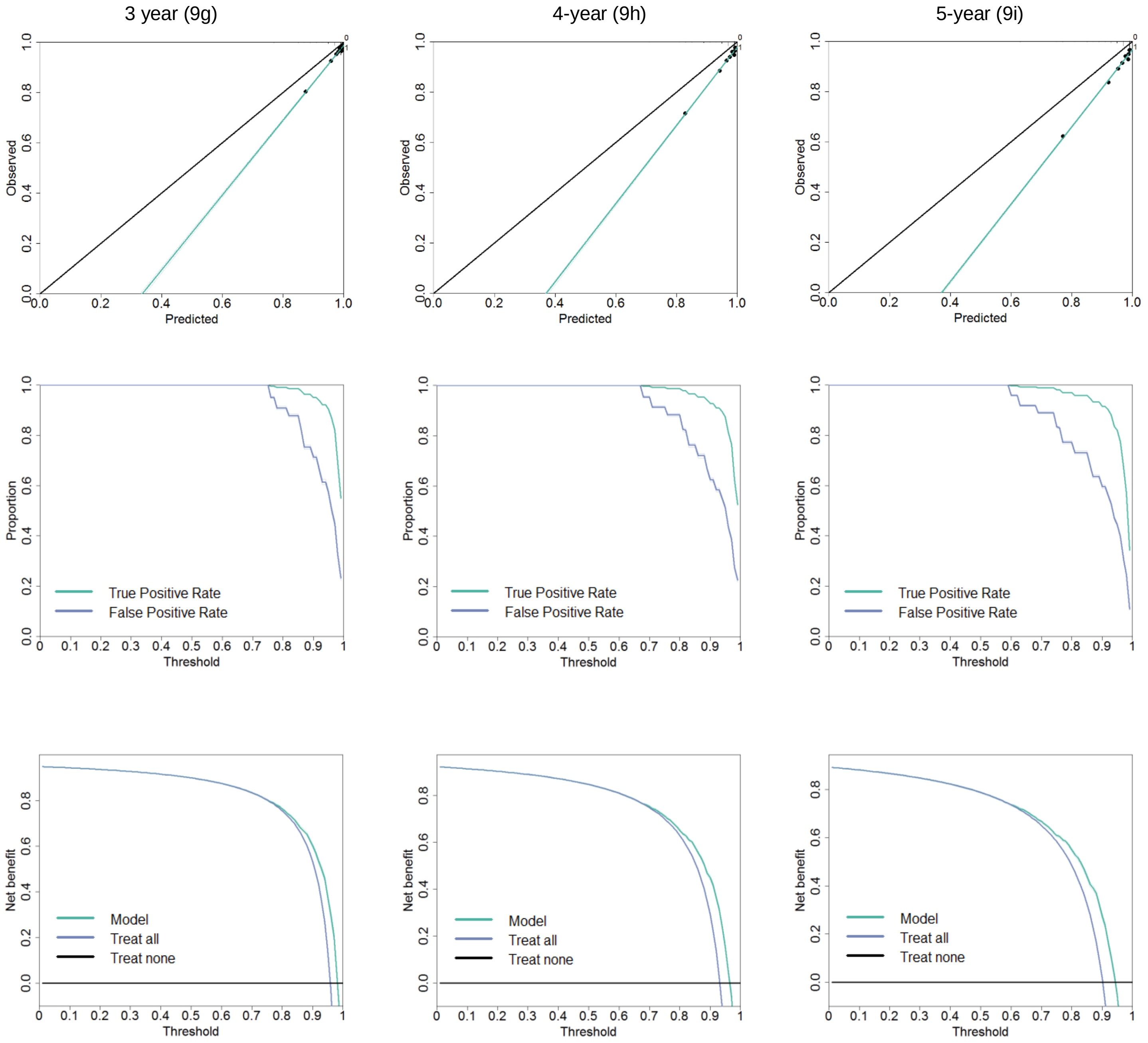

	3-year (9g)	4-year (9h)	5-year (9i)
AUC	0.749 (0.744 – 0.754)	0.747 (0.743 – 0.752)	0.747 (0.743 – 0.751)
Brier score	0.046 (0.045 – 0.047)	0.068 (0.067 – 0.069)	0.090 (0.089 – 0.092)
Scaled Brier score	0.049 (0.046 -0.052)	0.059 (0.056 – 0.062)	0.067 (0.063 – 0.069)

10. Wang et al. (10a, 10b, 10c, 10d)

Reference: Wang, Z., Cheng, Y., Chen, S., Shao, H., Chen, X., Wang, Z., ... & Ye, Z. (2020). Novel prognostic nomograms for female patients with breast cancer and bone metastasis at presentation. *Annals of translational medicine*, *8*(5).
Number of models presented in the paper: 4

Outcome:	3-year OS & BCSS<br>5-year OS & BCSS
Input variables:	Grade (I, II, III/IV)<br>Histological type (ductal, lobular, mixed, others)<br>Tumor size (cm) (<5, 5-10, >10)<br>Surgery (No, Yes)<br>Chemotherapy (No, Yes)<br>Number of metastatic organs except bone (0, 1, ≥2)<br>Molecular subtype (Luminal A, Luminal B, HER2+, Triple negative)
Inclusion criteria:	Bone metastasis, female, diagnosis confirmed by histology, age 20 – 80
Exclusion criteria:	Diagnosis according to autopsy or clinical/imaging findings. Unknown survival or <1month survival.
Original validation:	OS: 0.705 (95% CI: 0.691 – 0.719) & 0.678 (95% CI: 0.661 – 0.695) for training and validation cohorts respectively<br>BCSS: 0.710 (95% CI: 0.696 – 0.724) & 0.684 (95% CI: 0.666 – 0.702) for training and validation cohorts respectively

Variable name	Input	3-year (N = 5834)	5-year (N = 5375)
Grade	I	221 (3.8%)	195 (3.6%)
	II	1294 (22.2%)	1084 (20.2%)
	III	1009 (17.3%)	934 (17.4%)
	Missing	3310 (56.7%)	3162 (58.8%)
Histological type	Ductal	3714 (63.7%)	3371 (62.7%)
	Lobular	1014 (17.4%)	935 (17.4%)
	Mixed	183 (3.1%)	174 (3.2%)
	Others	923 (15.8%)	895 (16.7%)
Tumor size	<5 cm	907 (15.5%)	797 (14.8%)
	5 – 10 cm	231 (4%)	216 (4%)
	>10 cm	44 (0.8%)	42 (0.8%)
	Missing	4652 (79.7%)	4320 (80.4%)
Surgical treatment	No	4413 (75.6%)	4088 (76.1%)
	Yes	1421 (24.4%)	1287 (23.9%)
Chemotherapy	No	3426 (58.7%)	3164 (58.9%)
	Yes	2408 (41.3%)	2211 (41.1%)
No. of metastatic organs (except bone)	0	2771 (47.5%)	2510 (46.7%)
	1	1680 (28.8%)	1570 (29.2%)
	≥2	1383 (23.7%)	1295 (24.1%)
Molecular subtype	Luminal A	3576 (61.3%)	3242 (60.3%)
	Luminal B	676 (11.6%)	601 (11.2%)
	HER2+	349 (6%)	314 (5.8%)
	TNBC	436 (7.5%)	428 (8%)
	Missing	797 (13.7%)	790 (14.7%)
Outcome	Diseased	3149 (54%)	4124 (76.7%)
	Survived	2685 (46%)	1251 (23.3%)

Overall survival

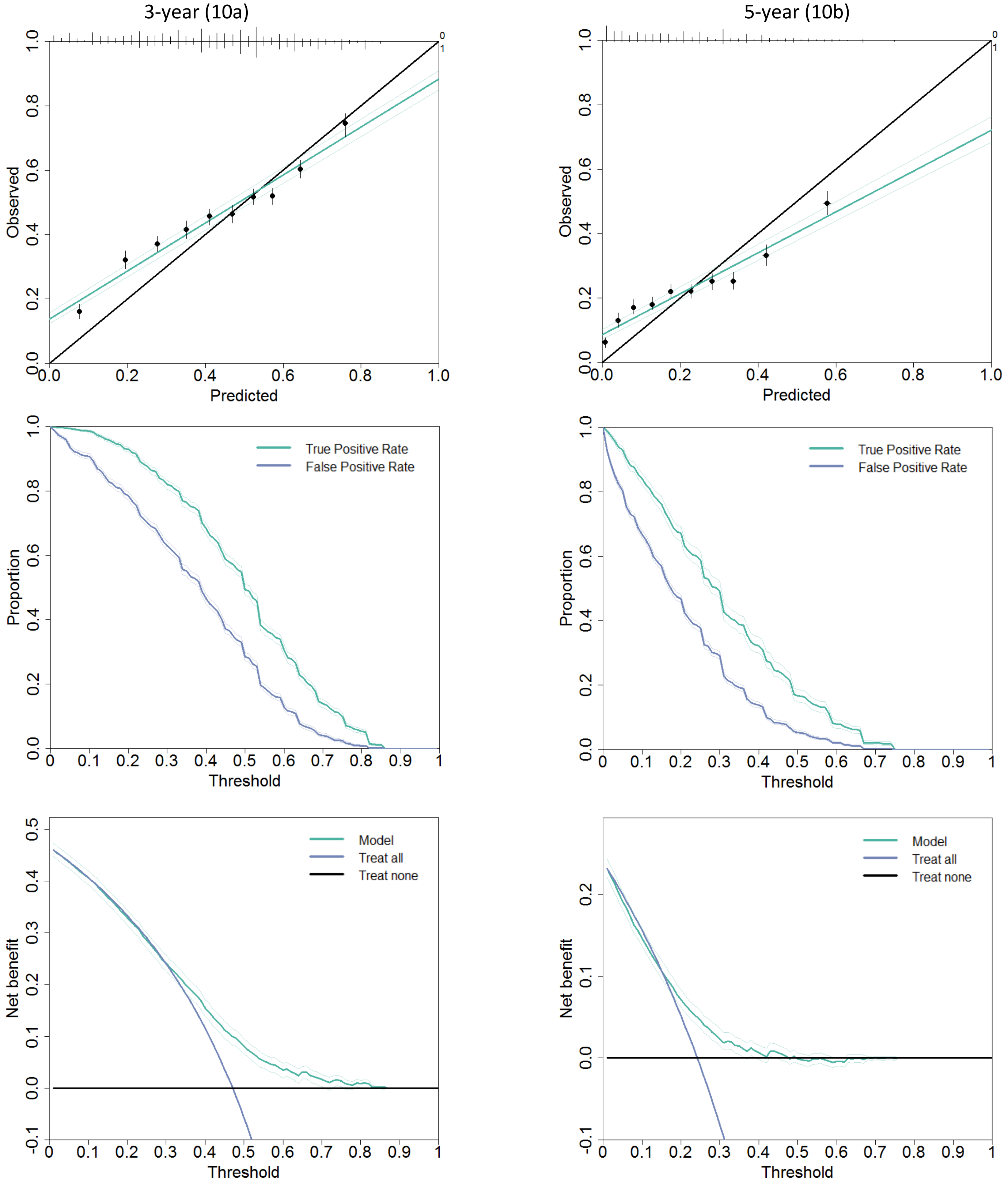

	3-year OS (10a)	5-year OS (10b)
AUC	0.665 (0.650 – 0.677)	0.663 (0.646 – 0.682)
Brier score	0.231 (0.227 – 0.236)	0.170 (0.165 – 0.178)
Scaled Brier score	0.070 (0.049 – 0.086)	0.044 (0.025 – 0.071)

Breast cancer specific survival

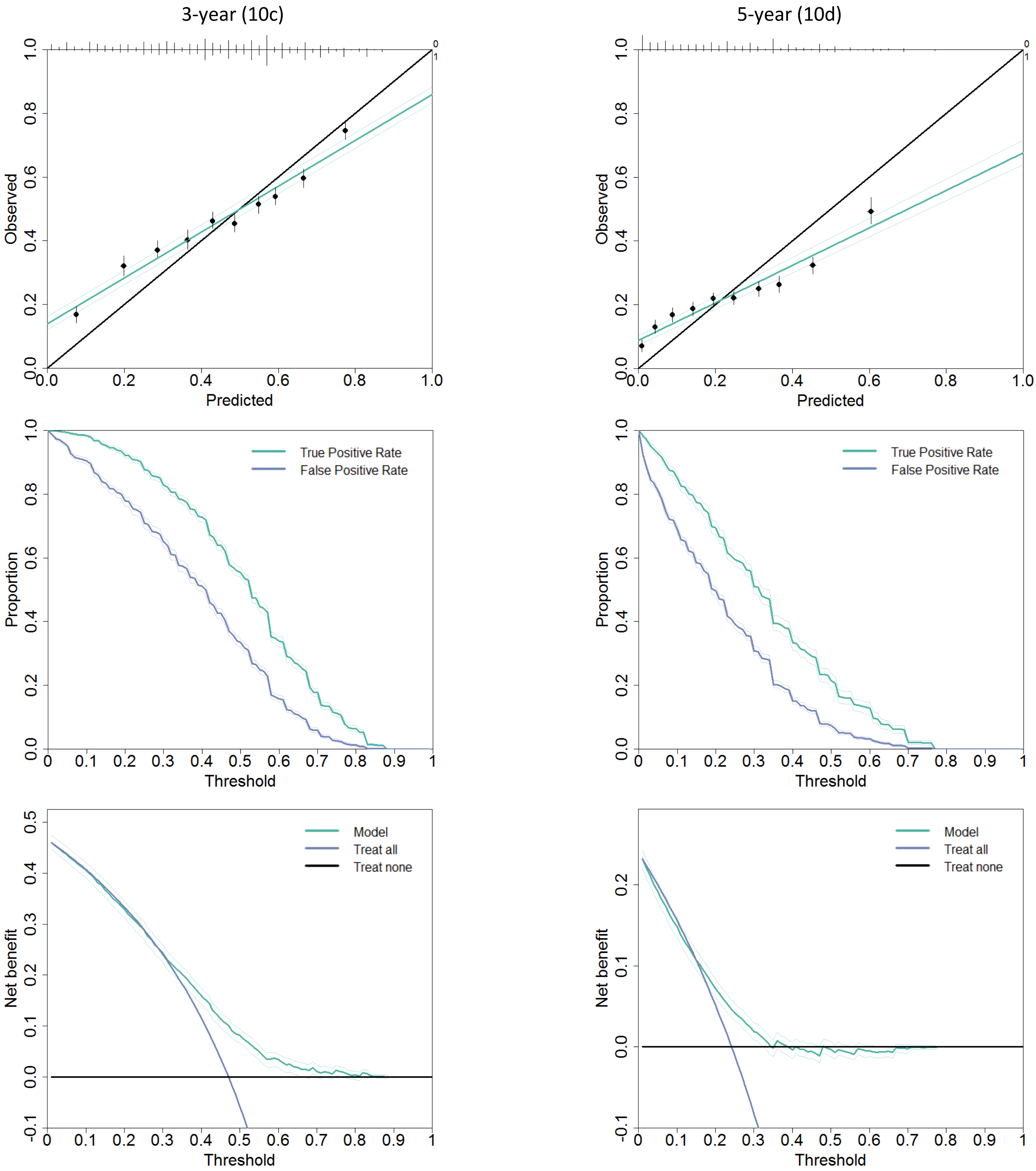

	3-year BCSS (10c)	5-year BCSS (10d)
AUC	0.663 (0.652 – 0.677)	0.661 (0.642 – 0.677)
Brier score	0.232 (0.227 – 0.237)	0.172 (0.166 – 0.178)
Scaled Brier score	0.066 (0.050 – 0.085)	0.036 (0.009 – 0.059)

11. Zheng et al. (Models 11a – 11l)

Reference: Zheng, Y., Zhong, G., Yu, K., Lei, K., & Yang, Q. (2020). Individualized prediction of survival benefit from Locoregional surgical treatment for patients with metastatic breast Cancer. *Frontiers in oncology*, *10*, 148.
Number of models presented in the paper: 12

Outcome:	1-year OS and BCSS (surgery, no surgery, preoperative)<br>3-year OS and BCSS (surgery, no surgery, preoperative)
Input variables:	Age (18-34, 35-49, 50-69, ≥70)<br>Grade (I, II, III, IV)<br>T stage (T1, T2, T3, T4)<br>Metastasis (Bone, Lung, Liver, Brain, other site, Multiple sites)<br>ER status (negative, positive)<br>PR status (negative, positive)<br>HER2 status (negative, positive)<br>Radiation (no, yes)<br>Chemotherapy (no, yes)
Inclusion criteria:	Metastatic breast cancer
Exclusion criteria:	Stage T0, Tx or Nx, missing data, incomplete follow-up, or with multiple primary cancers.
Original validation:<br>(internal and external cohorts, respectively).	OS surgery: 0.721 (0.707– 0.735) and 0.760 (0.730 – 0.790)<br>BCSS surgery: 0.722 (0.708 – 0.736) and 0.770 (0.740 – 0.800)<br>OS non-surgery: 0.664 (0.652 – 0.676) and 0.692 (0.674 – 0.710)<br>BCSS non surgery: 0.666 (0.654 – 0.678) and 0.696 (0.677 – 0.715)<br>OS preoperative: 0.713 (0.699 – 0.727) and 0.745 (0.714 – 0.776)<br>BCSS preoperative: 0.715 (0.701 – 0.729) and 0.758 (0.727 – 0.789)

Variable	Input	Preoperative		Surgical		Non-Surgical	
		1-year N = 8409	3-year N = 7577	1-year N = 1994	3-year N = 1769	1-year N = 6415	3-year N = 5808
Age	18 – 34	179 (2.1%)	153 (2%)	76 (3.8%)	63 (3.6%)	103 (1.6%)	90 (1.5%)
	35 – 49	1730 (20.6%)	1525 (20.1%)	622 (31.2%)	539 (30.5%)	1108 (17.3%)	986 (17%)
	50 – 69	3601 (42.8%)	3230 (42.6%)	867 (43.5%)	763 (43.1%)	2734 (42.6%)	2467 (42.5%)
	≥70	2899 (34.5%)	2669 (35.2%)	429 (21.5%)	404 (22.8%)	2470 (38.5%)	2265 (39%)
Grade	I	281 (3.3%)	219 (2.9%)	92 (4.6%)	79 (4.5%)	189 (2.9%)	140 (2.4%)
	II	1972 (23.5%)	1577 (20.8%)	692 (34.7%)	577 (32.6%)	1280 (20%)	1000 (17.2%)
	III	1716 (20.4%)	1512 (20%)	781 (39.2%)	694 (39.2%)	935 (14.6%)	818 (14.1%)
	IV	8 (0.1%)	8 (0.1%)	1 (0.1%)	1 (0.1%)	7 (0.1%)	7 (0.1%)
	Missing	4432 (52.7%)	4261 (56.2%)	428 (21.5%)	418 (23.6%)	4004 (62.4%)	3843 (66.2%)
T stage	T1	964 (11.5%)	885 (11.7%)	260 (13%)	238 (13.5%)	704 (11%)	647 (11.1%)
	T2	2896 (34.4%)	2564 (33.8%)	870 (43.6%)	767 (43.4%)	2026 (31.6%)	1797 (30.9%)
	T3	1288 (15.3%)	1116 (14.7%)	370 (18.6%)	314 (17.8%)	918 (14.3%)	802 (13.8%)
	T4	3261 (38.8%)	3012 (39.8%)	494 (24.8%)	450 (25.4%)	2767 (43.1%)	2562 (44.1%)
Metastasis	Bone	2526 (30%)	2221 (29.3%)	848 (42.5%)	729 (41.2%)	1678 (26.2%)	1492 (25.7%)
	Lung	379 (4.5%)	346 (4.6%)	113 (5.7%)	108 (6.1%)	226 (3.5%)	238 (4.1%)
	Liver	640 (7.6%)	580 (7.7%)	246 (12.3%)	218 (12.3%)	394 (6.1%)	362 (6.2%)
	Brain	42 (0.5%)	39 (0.5%)	8 (0.4%)	6 (0.3%)	34 (0.5%)	33 (0.6%)
	Other	726 (8.6%)	646 (8.5%)	329 (16.5%)	286 (16.2%)	397 (6.2%)	360 (6.2%)
	Multiple	3807 (45.3%)	3456 (45.6%)	387 (19.4%)	359 (20.3%)	3420 (53.3%)	3097 (53.3%)
ER	Negative	1882 (22.4%)	1731 (22.8%)	567 (28.4%)	505 (28.5%)	1315 (20.5%)	1226 (21.1%)
	Positive	6043 (71.9%)	5363 (70.8%)	1340 (67.2%)	1177 (66.5%)	4703 (73.3%)	4186 (72.1%)
	Missing	484 (5.8%)	483 (6.4%)	87 (4.4%)	87 (4.9%)	397 (6.2%)	396 (6.8%)

PR	Negative	3480 (41.4%)	3162 (41.7%)	923 (46.3%)	824 (46.6%)	2557 (39.9%)	2338 (40.3%)
	Positive	4299 (51.1%)	3785 (50%)	954 (47.8%)	828 (46.8%)	3345 (52.1%)	2957 (50.9%)
	Missing	630 (7.5%)	630 (8.3%)	117 (5.9%)	117 (6.6%)	513 (8%)	513 (8.8%)
HER2	Negative	5369 (63.8%)	4781 (63.1%)	1334 (66.9%)	1178 (66.6%)	4035 (62.9%)	3603 (62%)
	Positive	1857 (22.1%)	1628 (21.5%)	498 (25%)	429 (24.3%)	1359 (21.2%)	1199 (20.6%)
	Missing	1183 (14.1%)	1168 (15.4%)	162 (8.1%)	162 (9.2%)	1021 (15.9%)	1006 (17.3%)
Radio	No	6962 (82.8%)	6349 (83.8%)	933 (46.8%)	905 (51.2%)	6029 (94%)	5444 (93.7%)
	Yes	1447 (17.2%)	1228 (16.2%)	1061 (53.2%)	864 (48.8%)	386 (6%)	364 (6.3%)
Chemo	No	4715 (56.1%)	4282 (56.5%)	655 (32.8%)	638 (36.1%)	4060 (63.3%)	3644 (62.7%)
	Yes	3694 (43.9%)	3295 (43.5%)	1339 (67.2%)	1131 (63.9%)	2355 (36.7%)	2164 (37.3%)
Survival status	Deceased	2079 (24.7%)	4547 (60%)	189 (9.5%)	721 (40.8%)	1890 (29.5%)	3826 (65.9%)
	Survived	6330 (75.3%)	3030 (40%)	1805 (90.5%)	1048 (59.2%)	4525 (70.5%)	1982 (34.1%)

1-year overall survival

Pre-operative (11a) | Surgical patients (11c) | Non-surgical patients (11e)

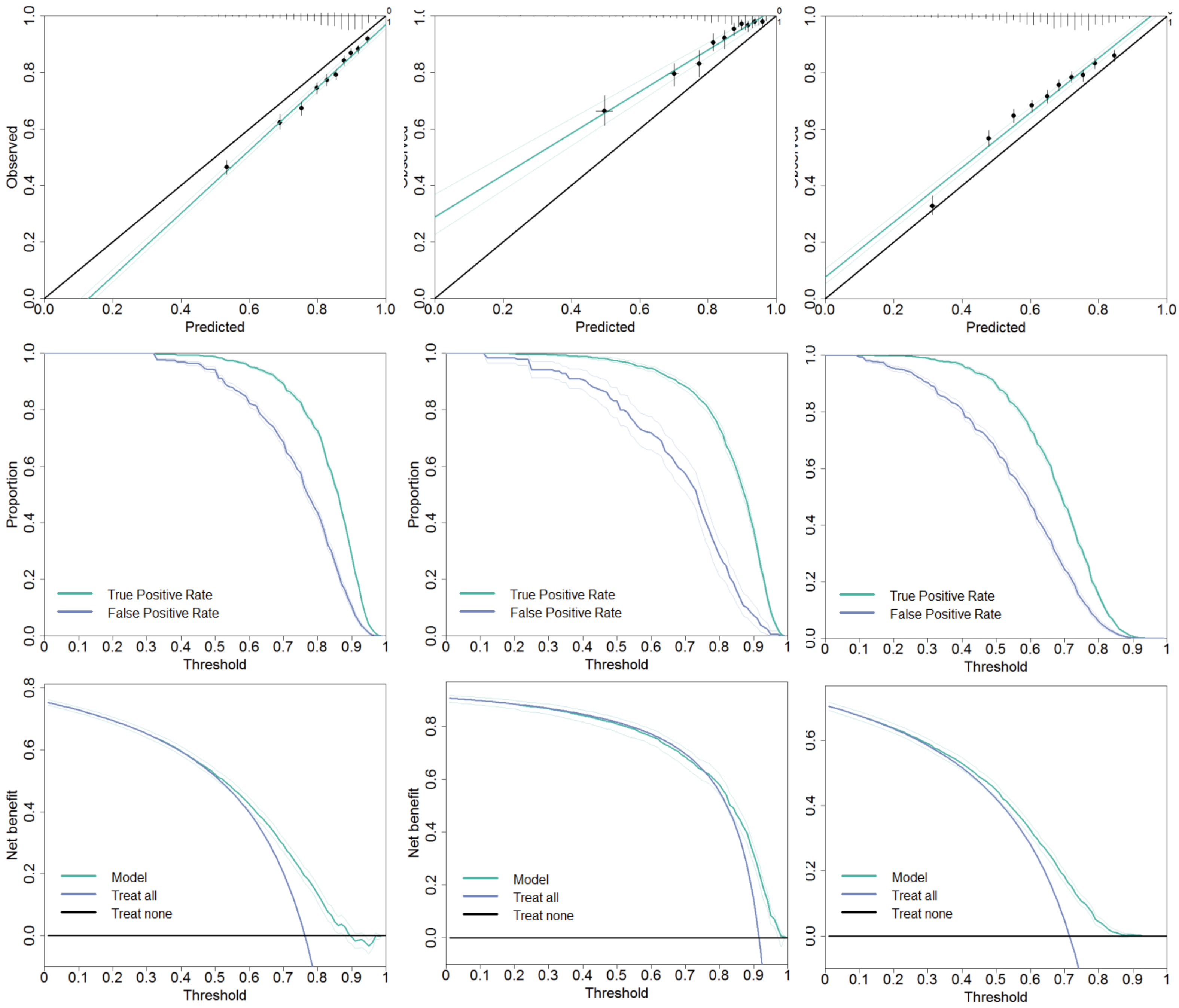

	Pre-operative (11a)	Surgical patients (11c)	Non-surgical patients (11e)
AUC	0.701 (0.689 – 0.714)	0.786 (0.757 – 0.818)	0.691 (0.675 – 0.704)
Brier score	0.171 (0.166 – 0.175)	0.085 (0.077 – 0.093)	0.190 (0.186 – 0.195)
Scaled Brier score	0.084 (0.074 – 0.095)	0.011 (-0.062 – 0.074)	0.087 (0.067 – 0.104)

3-year overall survival

Pre-operative (11b) | Surgical patients (11d) | Non-surgical patients (11f)

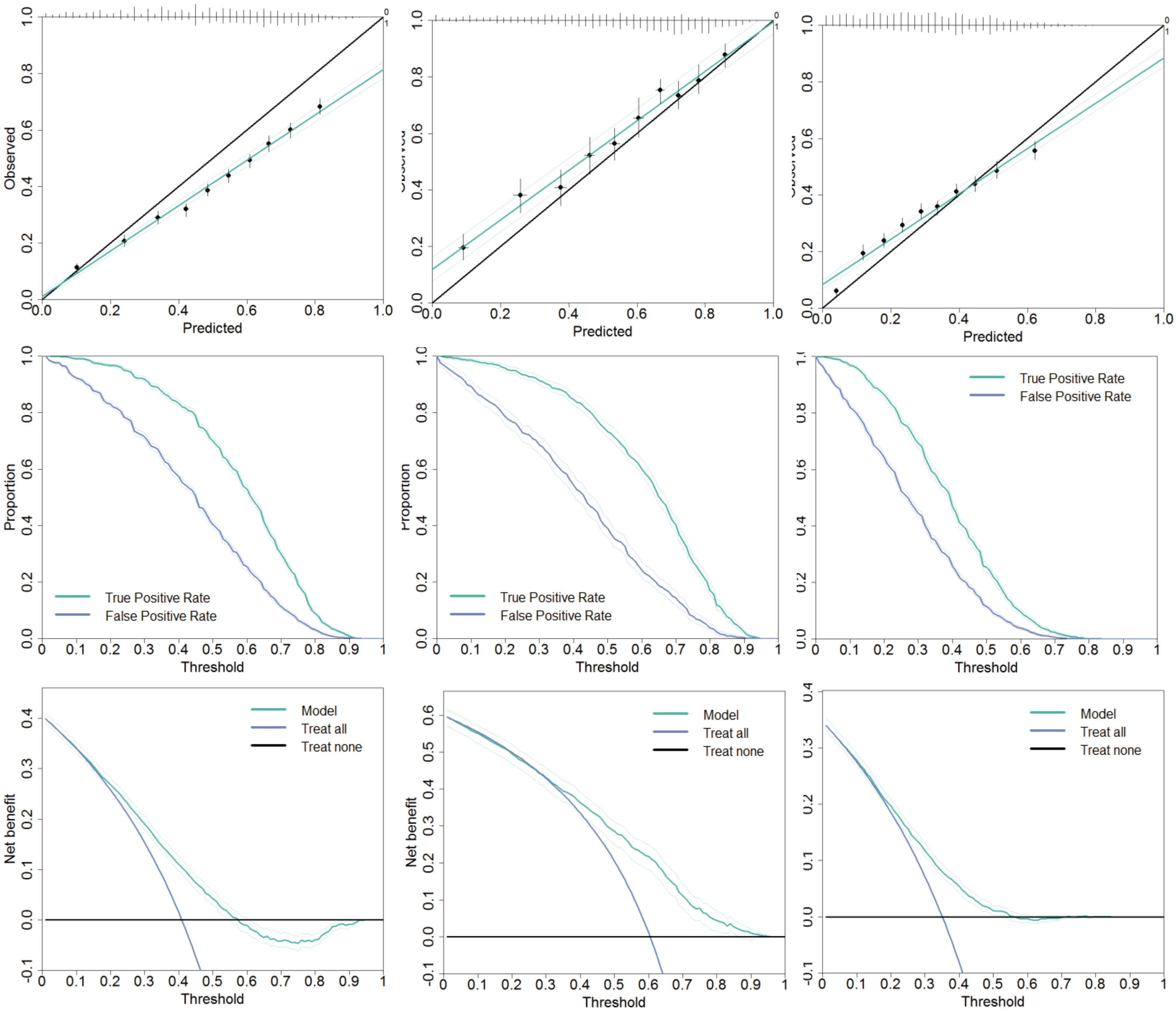

	Pre-operative (11b)	Surgical patients (11d)	Non-surgical patients (11f)
AUC	0.703 (0.694 – 0.714)	0.735 (0.714 – 0.759)	0.678 (0.661 – 0.691)
Brier score	0.22 (0.217 – 0.224)	0.207 (0.197 – 0.215)	0.208 (0.203 – 0.214)
Scaled Brier score	0.081 (0.066 – 0.099)	0.143 (0.110 – 0.181)	0.076 (0.056 – 0.091)

1-year breast cancer specific survival

Pre-operative (11g) | Surgical patients (11i) | Non-surgical patients (11k)

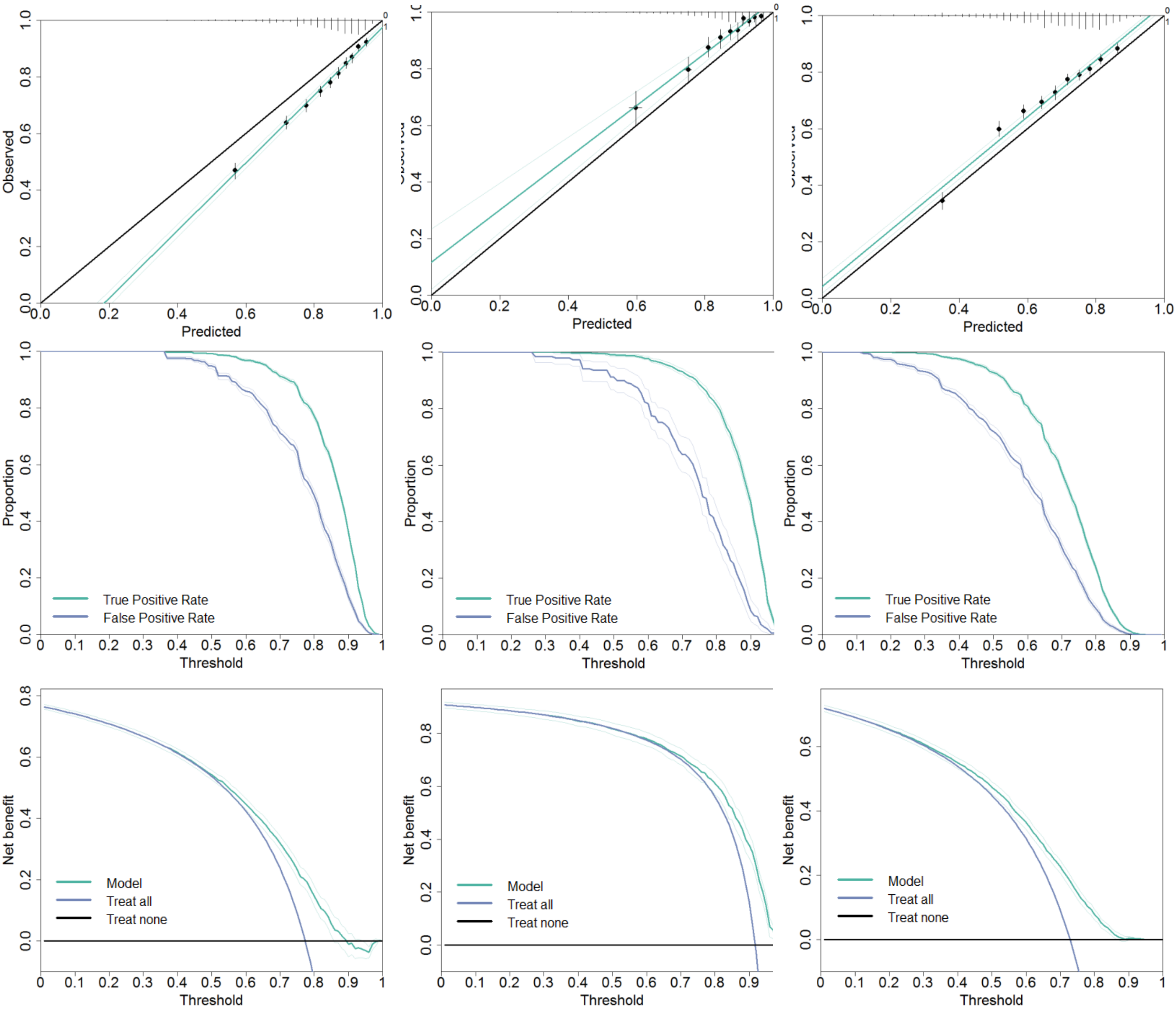

	Pre-operative (11g)	Surgical patients (11i)	Non-surgical patients (11k)
AUC	0.708 (0.695 – 0.723)	0.791 (0.758 – 0.822)	0.694 (0.680 – 0.707)
Brier score	0.166 (0.160 – 0.171)	0.077 (0.071 – 0.083)	0.182 (0.178 – 0.187)
Scaled Brier score	0.083 (0.070 – 0.098)	0.083 (0.038 – 0.132)	0.098 (0.084 – 0.112)

3-year breast cancer specific survival

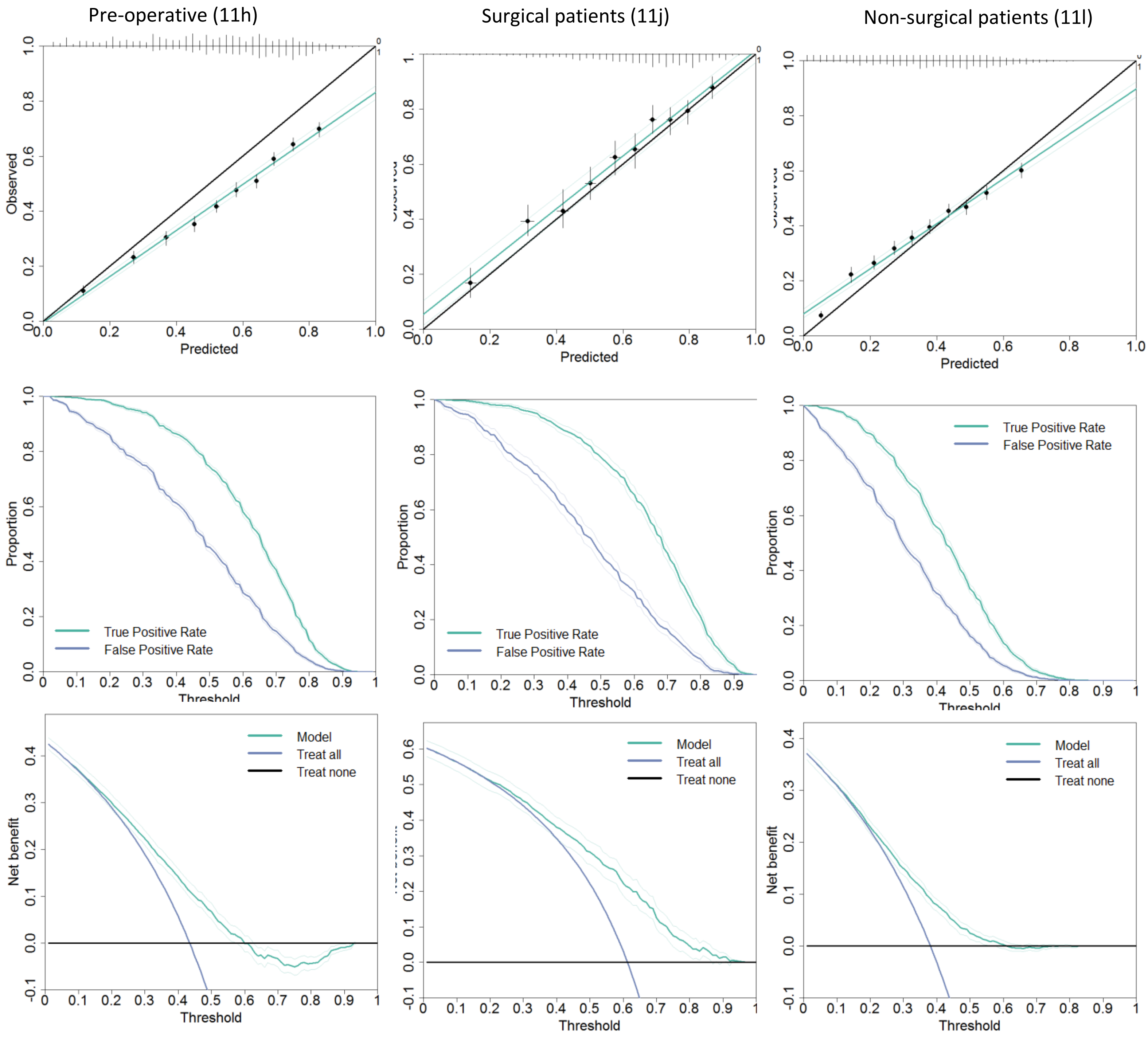

	Pre-operative (11h)	Surgical patients (11j)	Non-surgical patients (11l)
AUC	0.711 (0.697 – 0.722)	0.742 (0.718 – 0.764)	0.680 (0.664 – 0.693)
Brier score	0.221 (0.216 – 0.225)	0.199 (0.190 – 0.207)	0.213 (0.208 – 0.219)
Scaled Brier score	0.097 (0.076 – 0.114)	0.169 (0.131 – 0.203)	0.084 (0.067 – 0.101)

12. Janssen et al. (Models 12a & 12b)

Reference: Janssen, S., Haus, R., Schild, S. E., & Rades, D. (2020). A simple clinical instrument to predict the survival probability of breast cancer patients receiving radiotherapy for bone metastases. *Anticancer research*, *40*(1), 367-371.
Number of models presented in the paper: 2 models

Outcome:	1-year and 2-year overall survival.
Input variables:	ECOG performance score (<2, ≥2)<br>Visceral metastasis (no, yes)
Inclusion criteria:	Female breast cancer patients with bone metastasis undergoing radiation therapy
Exclusion criteria:	Spinal metastasis associated with cord compression.
Original validation:	Not assessed

Variable	Input	1-year N = 520	2-year N = 432
ECOG	<2	385 (74%)	113 (26.2%)
	≥2	135 (26%)	319 (73.8%)
Visceral metastasis	No	374 (71.9%)	306 (70.8%)
	Yes	146 (28.1%)	126 (29.2%)
Survival status	Deceased	96 (18.5%)	271 (62.7%)
	Survived	424 (81.5%)	161 (37.3%)

	1-year (12a)	2-year (12b)
AUC	0.630 (0.573 – 0.678)	0.657 (0.611 – 0.706)
Brier score	0.165 (0.151 – 0.182)	0.218 (0.199 – 0.238)
Scaled Brier score	-0.095 (-0.224 – 0.008)	0.058 (-0.019 – 0.159)

1-year OS (12a)

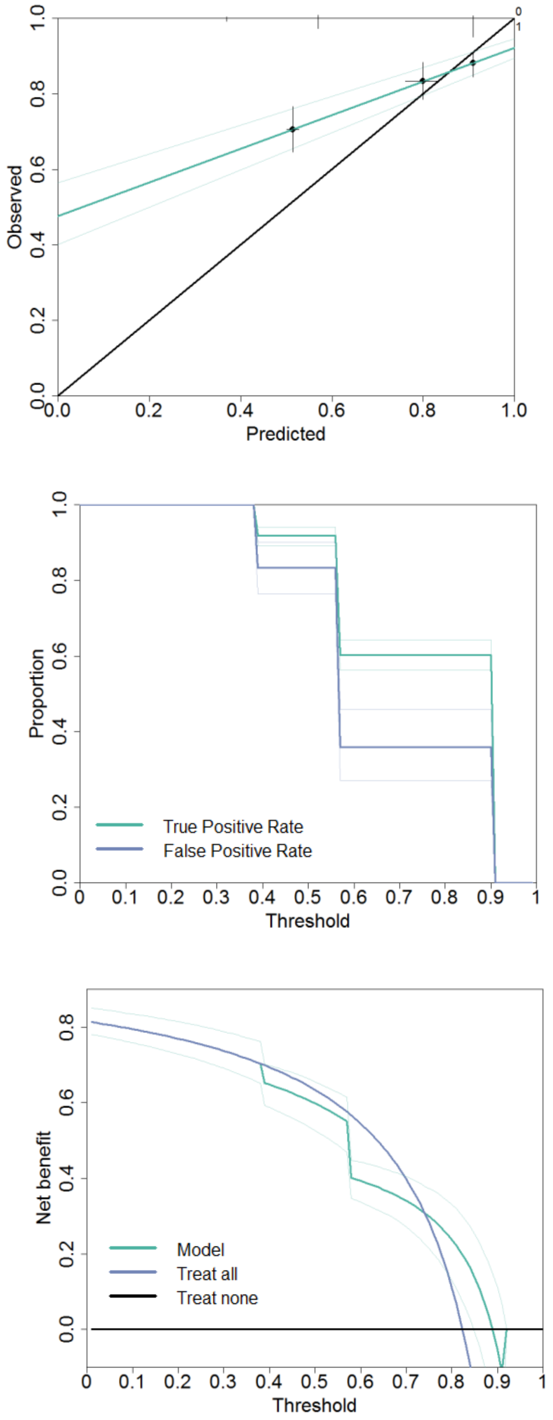

2-year OS (12b)

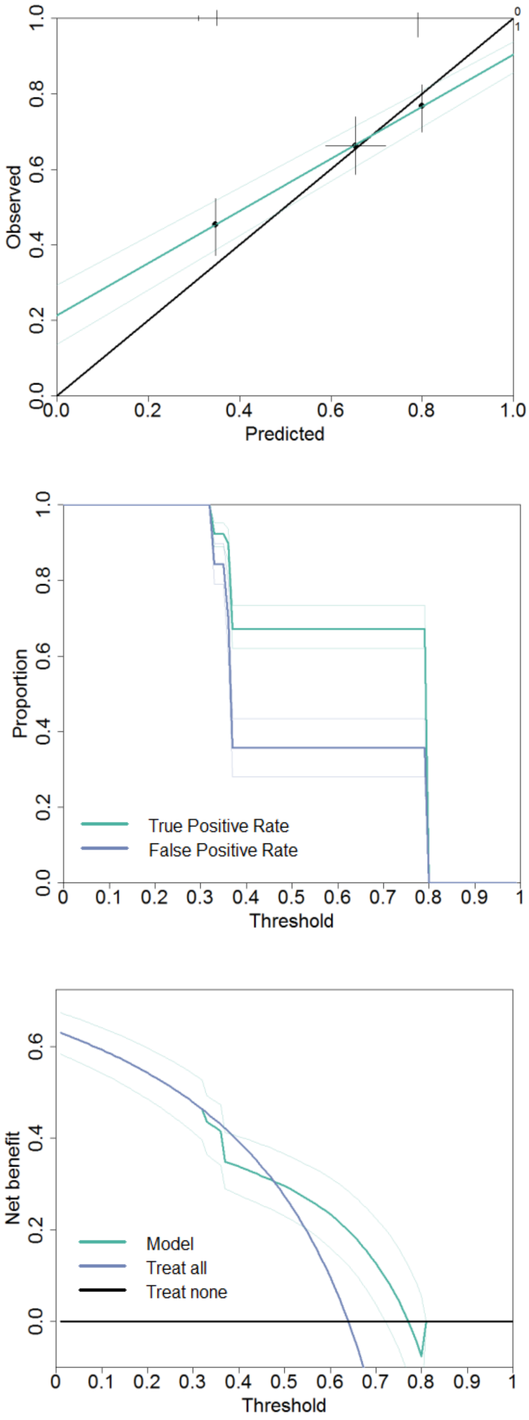

13. Wang et al. (Models 13a & 13b)

Reference: Wang, X., Feng, Z., Huang, Y., Li, H., Cui, P., Wang, D., ... & Chen, K. (2019). A nomogram to predict the overall survival of breast cancer patients and guide the postoperative adjuvant chemotherapy in China. *Cancer management and research*, *11*, 10029.

Number of models presented in the paper:

Outcome:	3-year and 5-year overall survival
Input variables:	Age (continuous per 5 year)<br>Tumor size (≤2cm, >2cm)<br>Nodal stage (N0, N+)<br>Grade (I, II, III/IV)<br>ER status (negative, positive)<br>PR status (negative, positive)
Inclusion criteria:	Aged 18-90, female, unilateral malignant breast cancer, radical mastectomy.<br>TNM stage I-III
Exclusion criteria:	neoadjuvant chemotherapy or radiotherapy, distant metastasis
Original validation:	3-year: 0.74 (95% CI: 0.69 – 0.78)<br>5-year: 0.72 (95% CI: 0.69 – 0.75)

Variable	Input	3-year N = 71758	5-year N = 65171
Age	Years (mean (sd))	62 (14)	62 (15)
Tumor size	≤2cm	32922 (45.9%)	29414 (45.1%)
	>2cm	34884 (48.6%)	31991 (49.1%)
	Missing	3952 (5.5%)	3766 (5.8%)
Nodal stage	N0	38824 (54.1%)	34747 (53.3%)
	N+	32934 (45.9%)	37011 (56.8%)
Grade	I	12265 (17.1%)	10984 (16.9%)
	II	32326 (45%)	28809 (44.2%)
	III/IV	22765 (31.7%)	21188 (32.5%)
	Missing	4402 (6.1%)	4190 (6.4%)
ER status	Negative	12492 (17.4%)	11765 (18.1%)
	Positive	56741 (79.1%)	50953 (78.2%)
	Missing	2525 (3.5%)	2453 (3.8%)
PR status	Negative	23316 (32.5%)	21593 (33.1%)
	Positive	44546 (62.1%)	39759 (61%)
	Missing	3896 (5.4%)	3819 (5.9%)
Survival status	Deceased	7177 (10%)	12480 (19.1%)
	Survived	64581 (90%)	52691 (80.9%)

	3-year (13a)	5-year (13b)
AUC	0.750 (0.745 – 0.756)	0.737 (0.731 – 0.742)
Brier score	0.088 (0.086 – 0.090)	0.148 (0.146 – 0.151)
Scaled Brier score	0.024 (0.021 – 0.027)	0.043 (0.038 – 0.048)

3-year (13a)

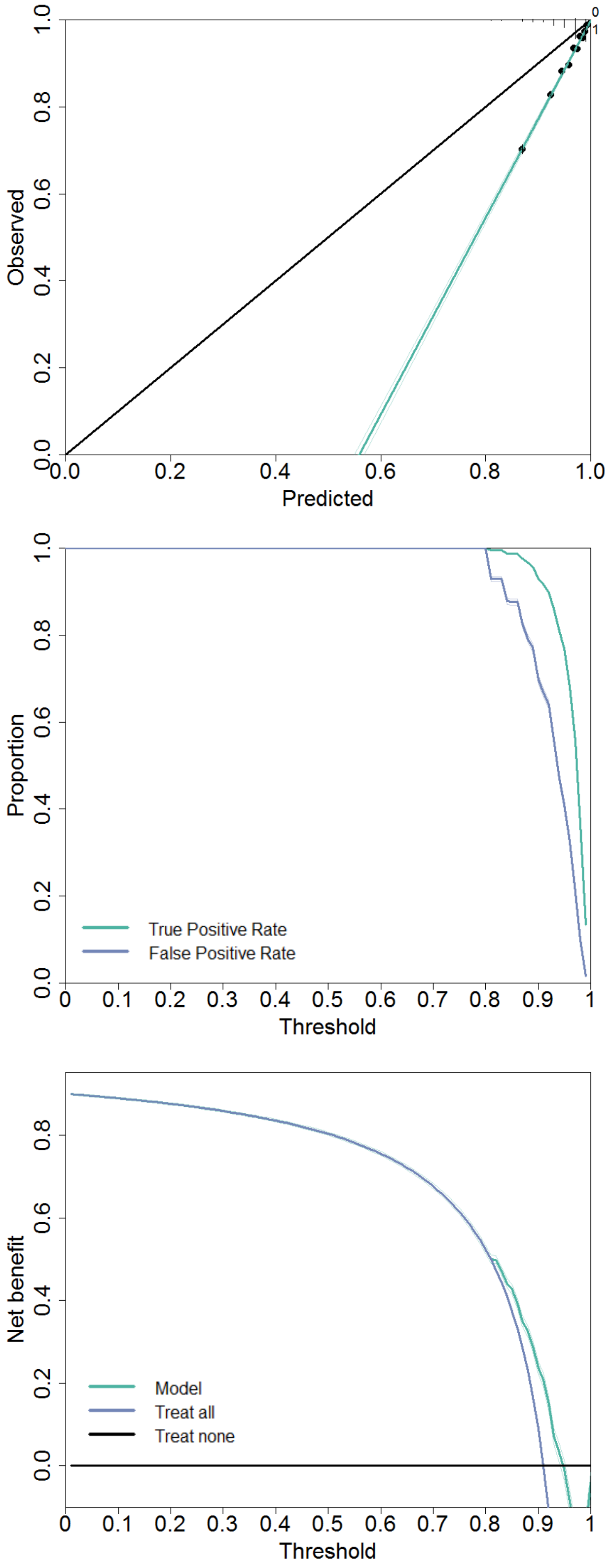

5-year (13b)

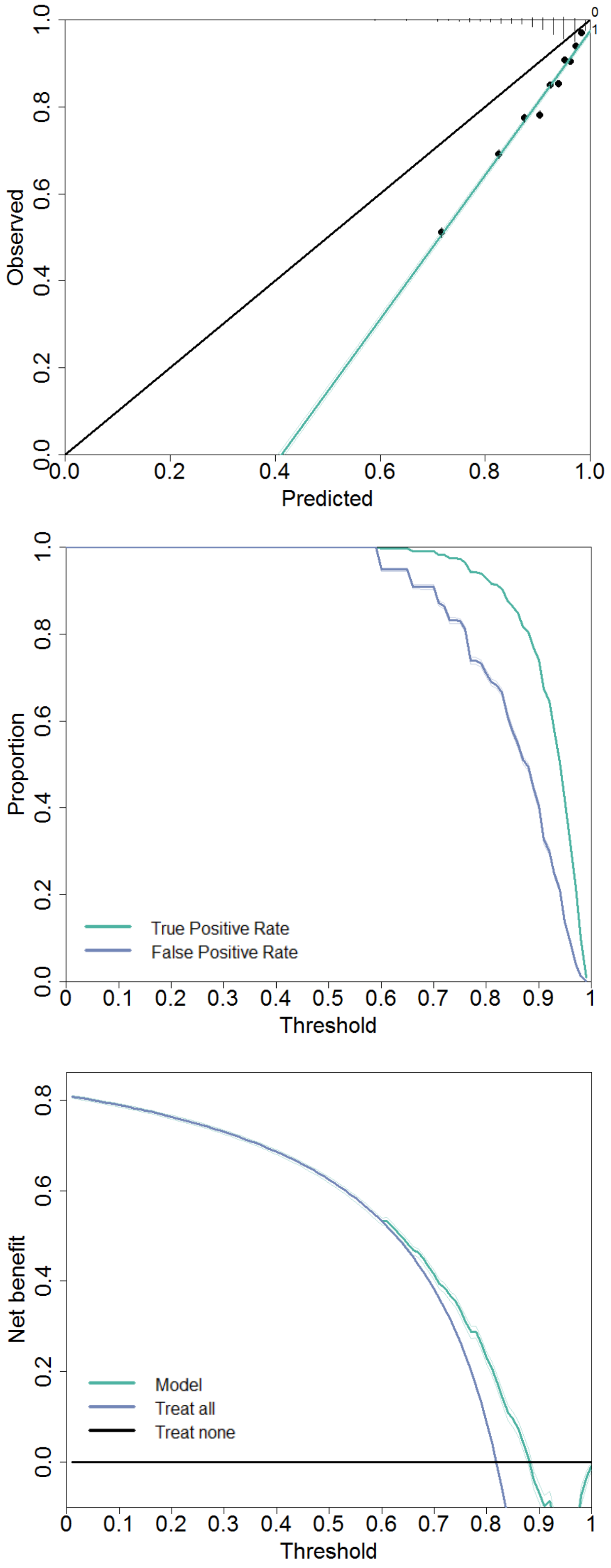

14. Abdel Rahman et al. (Model 14)

Abdel-Rahman, O. (2018). M-bioscore: proposing a new statistical model for prognostic factors in metastatic breast cancer patients. Journal of comparative effectiveness research, 7(09), 845-854.

Number of models developed in the paper: 1

Outcome:	4-year breast cancer specific survival
Input variables:	Metastatic site (distant LN, bone/skin, liver/lung, brain/multiple)<br>ER status (Negative, Positive)<br>PR status (Negative, Positive)<br>HER2-status (Negative, Positive)<br>Grade (I, II, III/IV)
Inclusion criteria:	Metastatic breast cancer
Exclusion criteria:	NA
Original validation:	0.665 (95% CI 0.646 – 0.685)

Variable name	Input	N (%) total = 10651
Metastatic site	Isolated distant LN	483 (4.5%)
	Isolated bone/skin	3625 (34.0%)
	Isolated liver/lung	1394 (13.1%)
	Isolated brain/multiple sites	5149 (48.3%)
ER status	Negative	2243 (21.1%)
	Positive	7519 (70.6%)
	Missing	889 (8.3%)
PR status	Negative	4236 (39.8%)
	Positive	5301 (49.8%)
	Missing	1114 (10.5%)
HER2 status	Negative	6608 (62%)
	Positive	1975 (18.5%)
	Missing	2068 (19.4%)
Tumor grade	I	342 (3.2%)
	II	1946 (18.3%)
	III	1968 (18.4%)
	Missing	6395 (60%)
4-year breast cancer specific survival	No	7686 (72.2%)
	Yes	2965 (27.8%)

AUC	0.666 (0.657 – 0.675)
Brier score	0.236 (0.234 – 0.238)
Scaled Brier score	-0.174 (-0.199 – - 0.149)

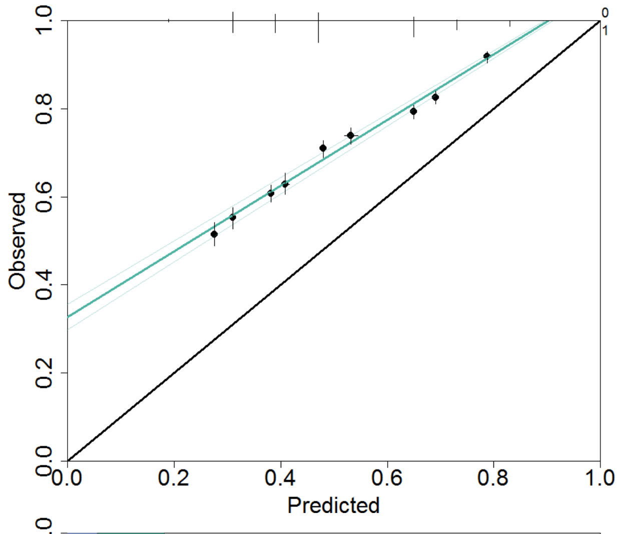

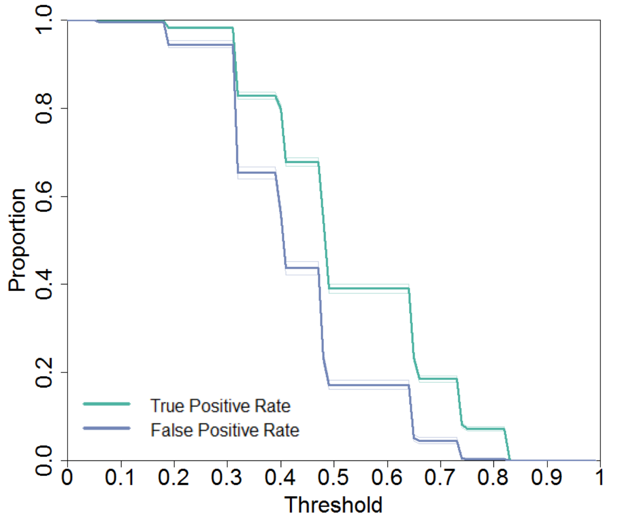

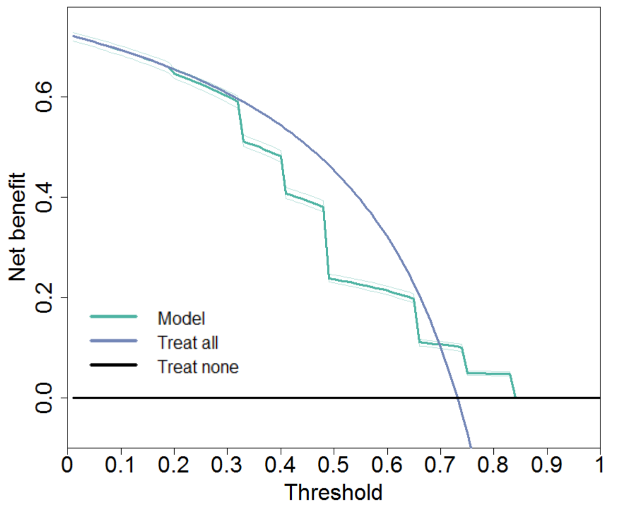

15. Elwood et al. (Model 15)

Reference: *Elwood, J. M., Tawfiq, E., TinTin, S., Marshall, R. J., Phung, T. M., Campbell, I., ... & Lawrenson, R. (2018). Development and validation of a new predictive model for breast cancer survival in New Zealand and comparison to the Nottingham prognostic index. BMC cancer, 18(1), 1-12.*

Number of models presented in the paper: 1

Outcome:	10-year disease specific survival
Input variables:	HER2 (negative, positive, Not assessed)*<br>Histology (Ductal, Lobular, Other)<br>Age (<40, 40-49, 50-59, 60-69, ≥70)<br>Ethnicity (Maori, Pacific, Euro, Other)**<br>Metastasis (No, Yes)<br>Tumor size (0-20, 20-50, ≥50)<br>Receptor status (+ve & +ve, +ve & -ve, -ve & -ve)<br>Grade (1, 2, 3)<br>Nodes positive (0, 1-3, 4-9, ≥10)
Inclusion criteria:	Women diagnosed with Invasive breast cancer
Exclusion criteria:	NA
Original validation:	0.84 for internal and 0.83 for external validation cohorts

*For validation, the option for HER2 "not assessed" was not used, as the model variable option refers to a time in which they did not collect HER2-status.

**European ethnicity was entered for all patients

Variable name	Input	N (%) total = 48661
HER2	Negative	29376 (60.4%)
	Positive	4953 (10.2%)
	Missing	14332 (29.5%)
Histology	Ductal	36764 (75.6%)
	Lobular	5953 (12.2%)
	Other	5944 (12.2%)
Age	<40	2120 (4.4%)
	40 – 49	6637 (13.6%)
	50 – 59	10042 (20.6%)
	60 – 69	10863 (22.3%)
	≥70	18999 (39%)
Metastasis	No	47144 (96.9%)
	Yes	1517 (3.1%)
Tumor size	0-20	24054 (49.4%)
	20-50	21753 (44.7%)
	≥50	2854 (5.9%)
Receptor status	ER+ and PR+	26918 (55.3%)
	ER+ or PR+	8577 (17.6%)
	ER- and PR -	9948 (20.4%)
	Missing	3218 (6.6%)
Grade	I	8655 (17.8%)
	II	19852 (40.8%)
	III	16805 (34.5%)
	Missing	3349 (6.9%)
Positive lymph nodes	0	26825 (55.1%)
	1 – 3	13595 (27.9%)
	4 – 9	3464 (7.1%)
	>9	2961 (6.1%)
10-year survival status	Survived	41979 (86.3%)
	Deceased	6682 (13.7%)

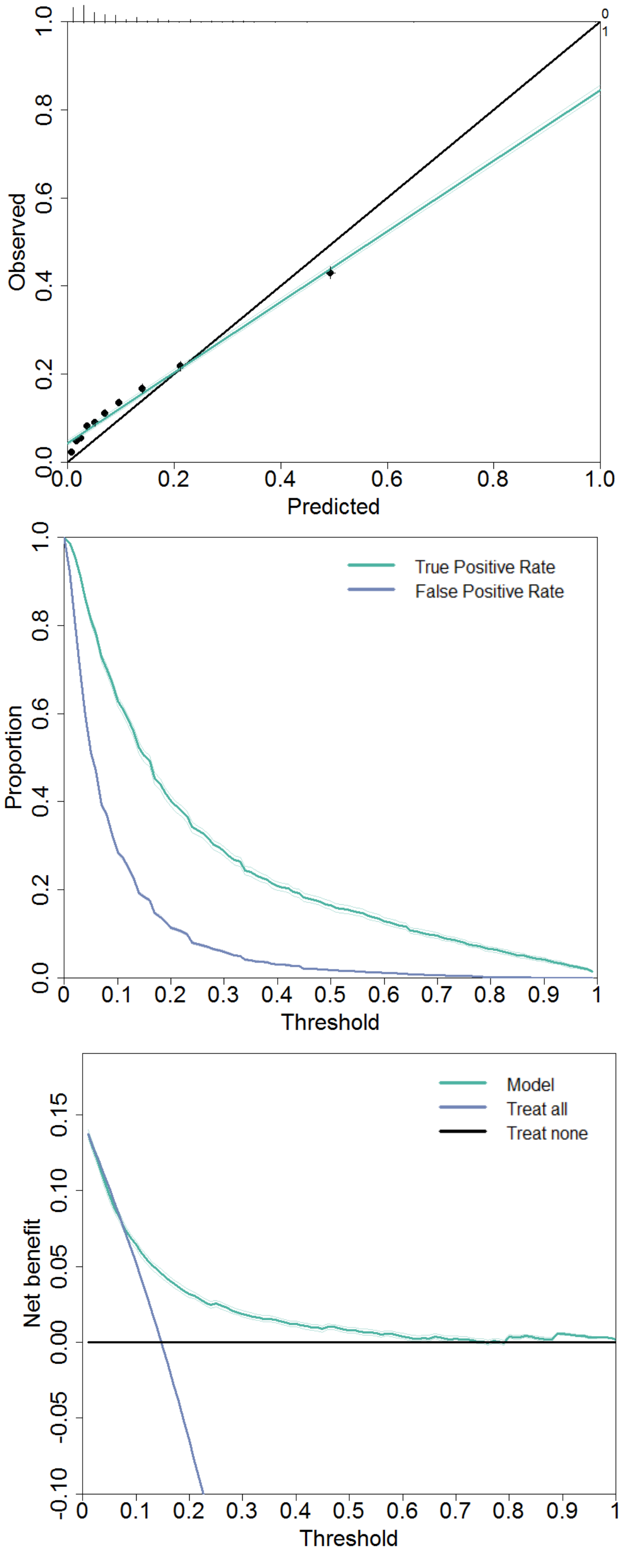

AUC	0.740 (0.733 – 0.745)
Brier score	0.105 (0.103 – 0.107)
Scaled Brier Score	0.116 (0.105 – 0.125)

16. Paredes Aracil et al. (Models 16a & 16b)

Paredes-Aracil, E., Palazón-Bru, A., Folgado-de la Rosa, D. M., Ots-Gutiérrez, J. R., Compañ-Rosique, A. F., & Gil-Guillén, V. F. (2017). A scoring system to predict breast cancer mortality at 5 and 10 years. Scientific reports, 7(1), 1-8.

Number of models presented in the paper: 2

Outcome:	5-year breast cancer specific mortality<br>10-year breast cancer specific mortality
Input variables:	Age (<50, ≥50)<br>Stage (0, I, II, III, IV)<br>Grade (I, II, III)<br>Previous breast cancer (no, yes)<br>Multifocal tumor (no, yes)
Inclusion criteria:	Women diagnosed with breast cancer
Exclusion criteria:	NA
Original validation:	0.83

Variable	Input	5-year N = 195349	10-year N = 113615
Age	<50	43386 (22.2%)	26706 (23.5%)
	≥50	151963 (77.8%)	86909 (76.5%)
Stage	0	23067 (11.8%)	10827 (9.5%)
	I	77502 (39.7%)	41292 (36.3%)
	II	63479 (32.5%)	37981 (33.4%)
	III	19891 (10.2%)	12864 (11.3%)
	IV	11410 (5.8%)	10651 (9.4%)
Grade	I	38936 (19.9%)	20984 (18.5%)
	II	74280 (38%)	41050 (36.1%)
	III	53141 (27.2%)	32072 (28.2%)
	Missing	28992 (14.8%)	19509 (17.2%)
Previous breast cancer	No	188581 (96.5%)	110925 (97.6%)
	Yes	6768 (3.5%)	2690 (2.4%)
Multifocal tumor	No	153973 (78.8%)	86102 (75.8%)
	Yes	28484 (14.6%)	16103 (14.2%)
	Missing	12892 (6.6%)	11410 (10%)
Survival	Survived	182728 (93.5%)	97970 (86.2%)
	Deceased	12621 (6.5%)	15645 (13.8%)

	5-year BCSS (16a)	10-year BCSS (16b)
AUC	0.911 (0.908 – 0.913)	0.877 (0.874 – 0.881)
Brier score	0.054 (0.054 – 0.055)	0.099 (0.098 – 0.100)
Scaled Brier score	0.002 (-0.011 – 0.017)	0.019 (0.004 – 0.036)

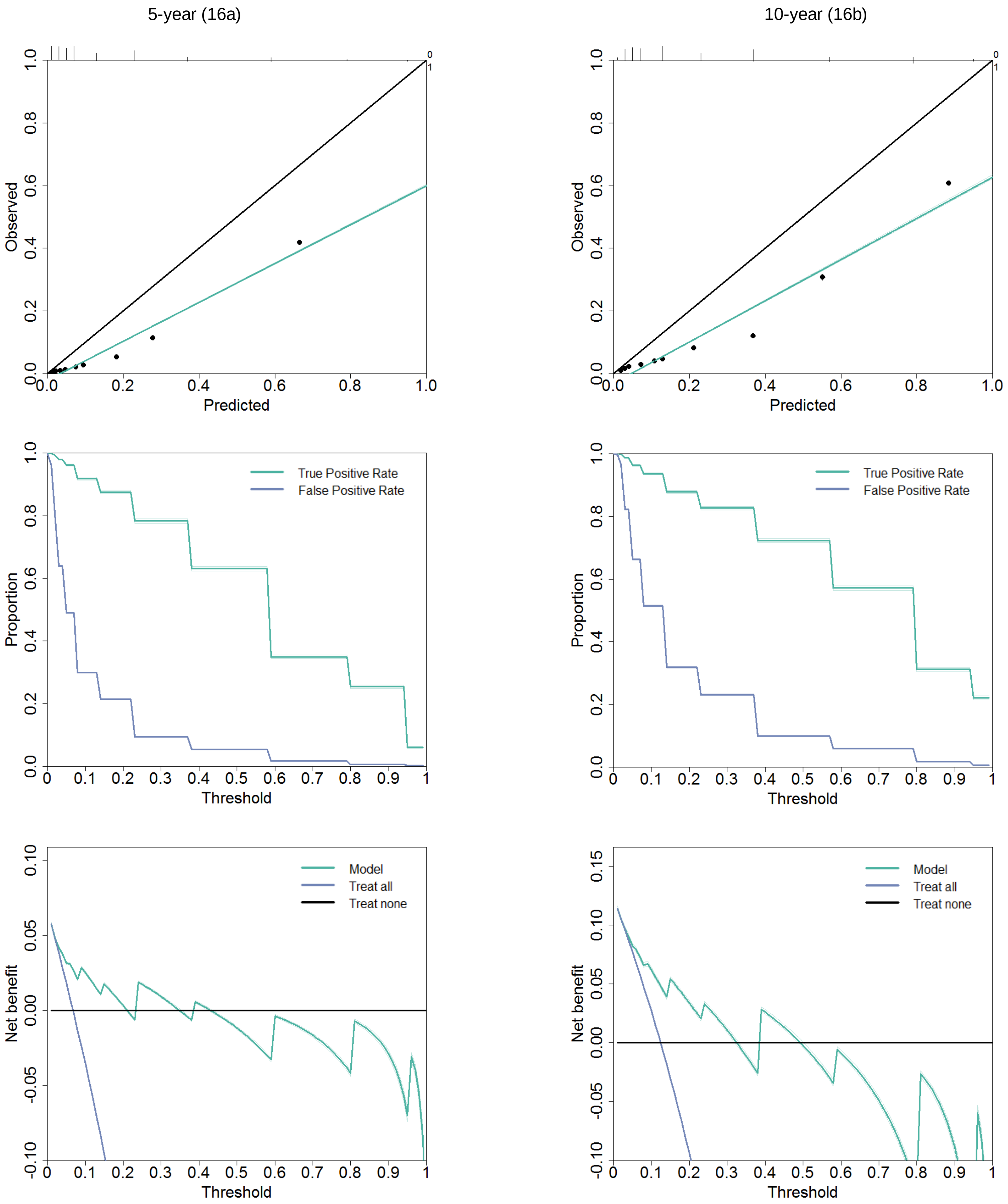
5-year (16a)
10-year (16b)
Observed
Predicted
Proportion
True Positive Rate
False Positive Rate
Threshold
Net benefit
Model
Treat all
Treat none

17. Wen et al. (Models 17a & 17b)

Reference: Wen, J., Yang, Y., Liu, P., Ye, F., Tang, H., Huang, X., ... & Xie, X. (2017). Development and validation of a nomogram for predicting survival on the base of modified lymph node ratio in breast cancer patients. The Breast, 33, 14-22.
Number of models presented in the paper: 2

Outcome:	5-year breast cancer specific survival<br>10-year breast cancer specific survival
Input variables:	Menopausal status (premenopause, menopause)<br>T stage (T1, T2, T3)<br>pN stage (N0, N1, N2, N3)<br>mLNR (continuous)<br>ER status (negative, positive)<br>HER2 status (negative, positive)
Inclusion criteria:	Female, received surgical treatment, invasive ductal or invasive lobular carcinoma.
Exclusion criteria:	Neoadjuvant chemo or radiotherapy, previous cancer, metastasis at diagnosis, SLNB only, bilateral cancer
Original validation:	0.747 and 0.789 for training and validation cohorts, respectively

Variable name	Input	5-year BCSS<br>N (%) total = 45517	10-year BCSS<br>N (%) total = 35270
Menopause	Pre	3526 (7.7%)	1903 (5.4%)
	Peri	645 (1.4%)	251 (0.7%)
	Post	9170 (20.1%)	4088 (11.6%)
	Missing	32176 (70.7%)	29028 (82.3%)
T stage	T1	21330 (46.9%)	16604 (47.1%)
	T2	21405 (47%)	16600 (47.1%)
	T3	2782 (6.1%)	2066 (5.9%)
P stage	N0	9745 (21.4%)	8207 (23.3%)
	N1	24605 (54.1%)	18597 (52.7%)
	N2	7102 (15.6%)	5350 (15.2%)
	N3	4065 (8.9%)	3116 (8.8%)
mLNR	Ratio (median (IQR))	0.14 (0.08 – 0.32)	0.14 (0.08 – 0.30)
ER status	Negative	7661 (16.8%)	6406 (18.2%)
	Positive	36304 (79.8%)	27502 (78%)
	Missing	1552 (3.4%)	1362 (3.9%)
HER2 status	Negative	29789 (65.4%)	21429 (60.8%)
	Positive	5864 (12.9%)	4323 (12.3%)
	Missing	9864 (21.7%)	9518 (27%)
Breast cancer specific survival	Died	2239 (4.9%)	3301 (9.4%)
	Survived	43278 (95.1%)	31969 (90.6%)

5-year disease specific survival (17a)

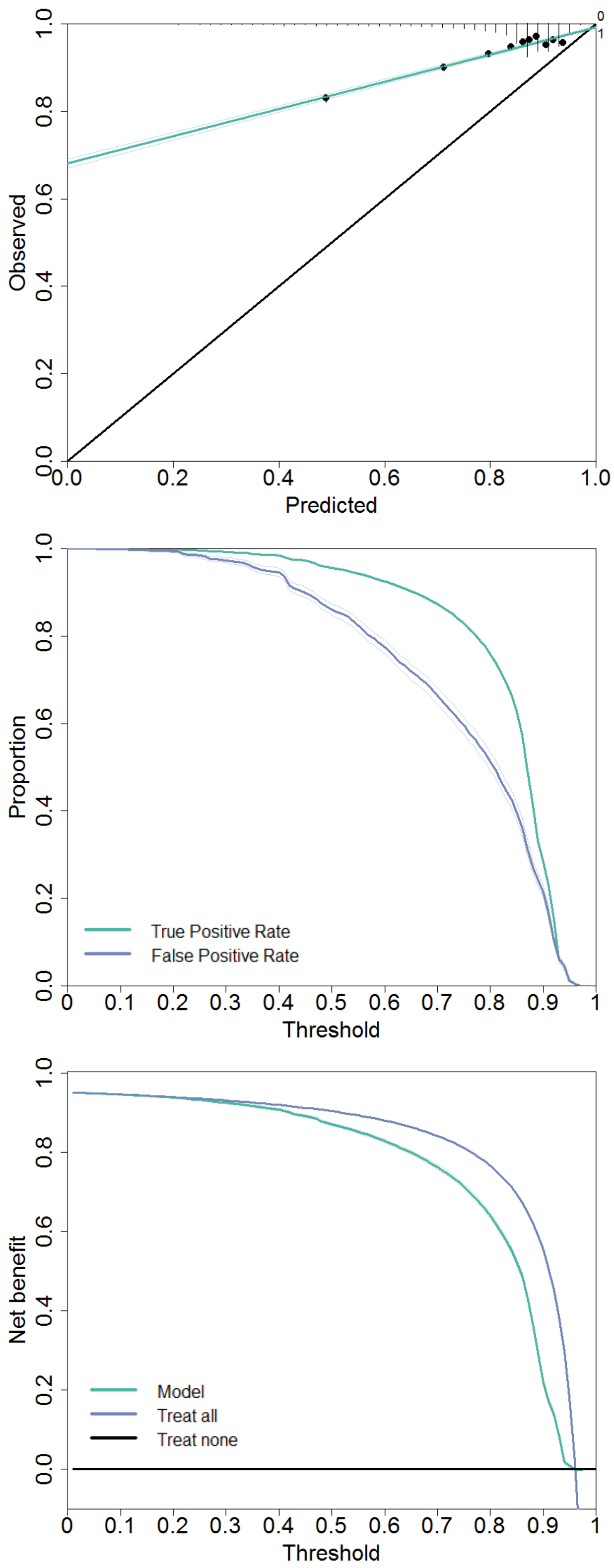

10-year disease specific survival (17b)

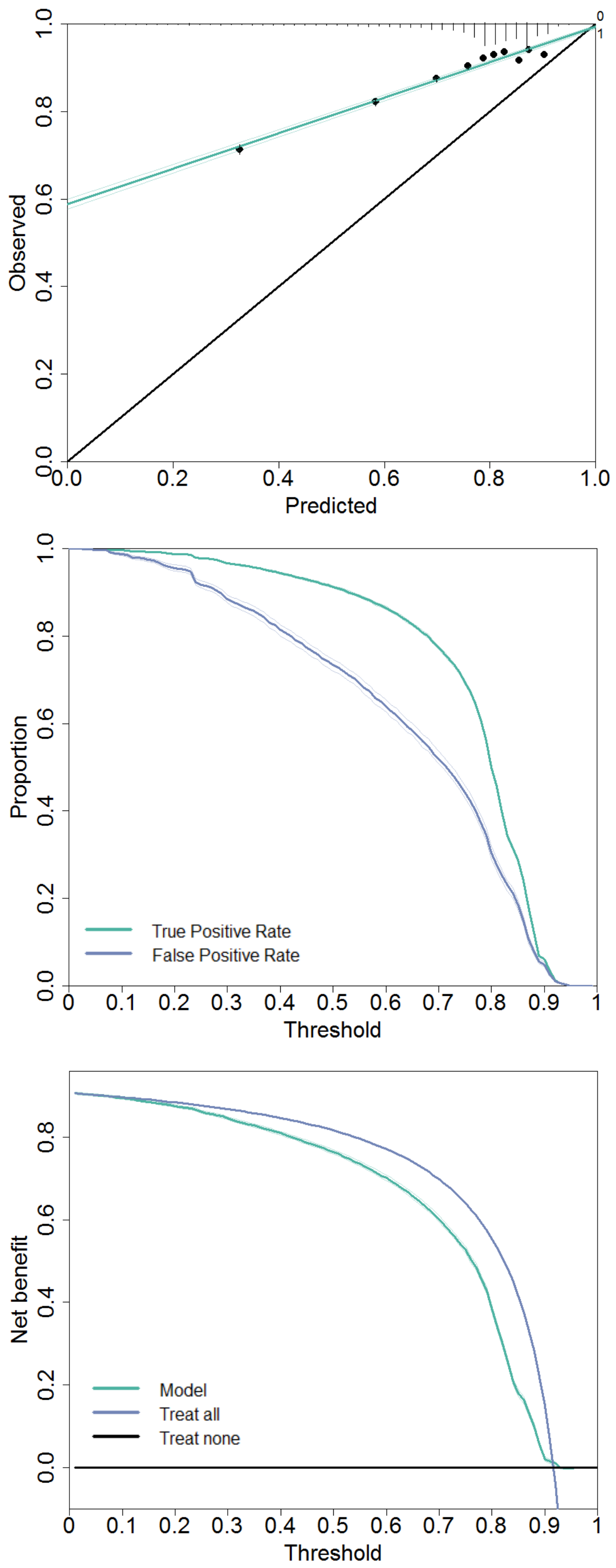

Disease specific survival	5-year	10-year
AUC	0.641 (0.628 – 0.653)	0.650 (0.639 – 0.660)
Brier score	0.074 (0.073 – 0.076)	0.124 (0.123 – 0.126)
Scaled Brier score	-0.591 (-0.634 – -0.548)	-0.465 (-0.501 – -0.433)

18. Wen et al. (Models 18a & 18b)

Wen, J., Ye, F., He, X., Li, S., Huang, X., Xiao, X., & Xie, X. (2016). Development and validation of a prognostic nomogram based on the log odds of positive lymph nodes (LODDS) for breast cancer. *Oncotarget*, *7*(15), 21046.

Models presented: 2

Outcome:	5-year & 10-year breast cancer specific survival
Input variables:	ER status (negative, positive)<br>HER2 status (negative, positive)<br>T-stage (T1, T2, T3)<br>pN stage (pN0, pN1, pN2, pN3)<br>Log Odds of positive lymph nodes (continuous)<br>Menopause (No, Yes)
Inclusion criteria:	Radical mastectomy, female, invasive ductal or invasive lobular carcinoma.
Exclusion criteria:	Neoadjuvant treatment, previous cancers, metastasis
Original validation:	0.745 and 0.796 in training and validation cohorts, respectively.

Variable	Input	5-year N = 41122	10-year N = 26164
ER status	Negative	6981 (17%)	4918 (18.8%)
	Positive	32804 (79.8%)	20082 (76.8%)
	Missing	1337 (3.3%)	1164 (4.4%)
HER2 status	Negative	27671 (67.3%)	15416 (58.9%)
	Positive	5671 (13.8%)	3407 (13%)
	Missing	7780 (18.9%)	7341 (28.1%)
T stage	T1	19845 (48.3%)	12275 (46.9%)
	T2	18811 (45.7%)	12331 (47.1%)
	T3	2466 (6%)	1558 (6%)
N stage	N0	21586 (52.5%)	13203 (50.5%)
	N1	13303 (32.4%)	8497 (32.5%)
	N2	3981 (9.7%)	2837 (10.8%)
	N3	2252 (5.5%)	1627 (6.2%)
Log odds positive LN	Median (IQR)	-1.609 (-2.197 – -1.099)	-1.609 (-2.269 – -1.099)
Menopause	No	4866 (11.8%)	1371 (5.2%)
	Yes	10389 (25.3%)	2214 (8.5%)
	Missing	25867 (62.9%)	22579 (86.3%)
Survival status	Deceased	1708 (4.2%)	2499 (9.6%)
	Survived	39414 (95.8%)	23665 (90.4%)

	5-year (18a)	10-year (18b)
AUC	0.642 (0.623 – 0.656)	0.647 (0.634 – 0.658)
Brier score	0.045 (0.044 – 0.046)	0.088 (0.085 – 0.090)
Scaled Brier score	-0.239 (-0.267 – -0.209)	-0.157 (-0.182 – -0.134)

5-year BCSS (18a)

10-year BCSS (18b)

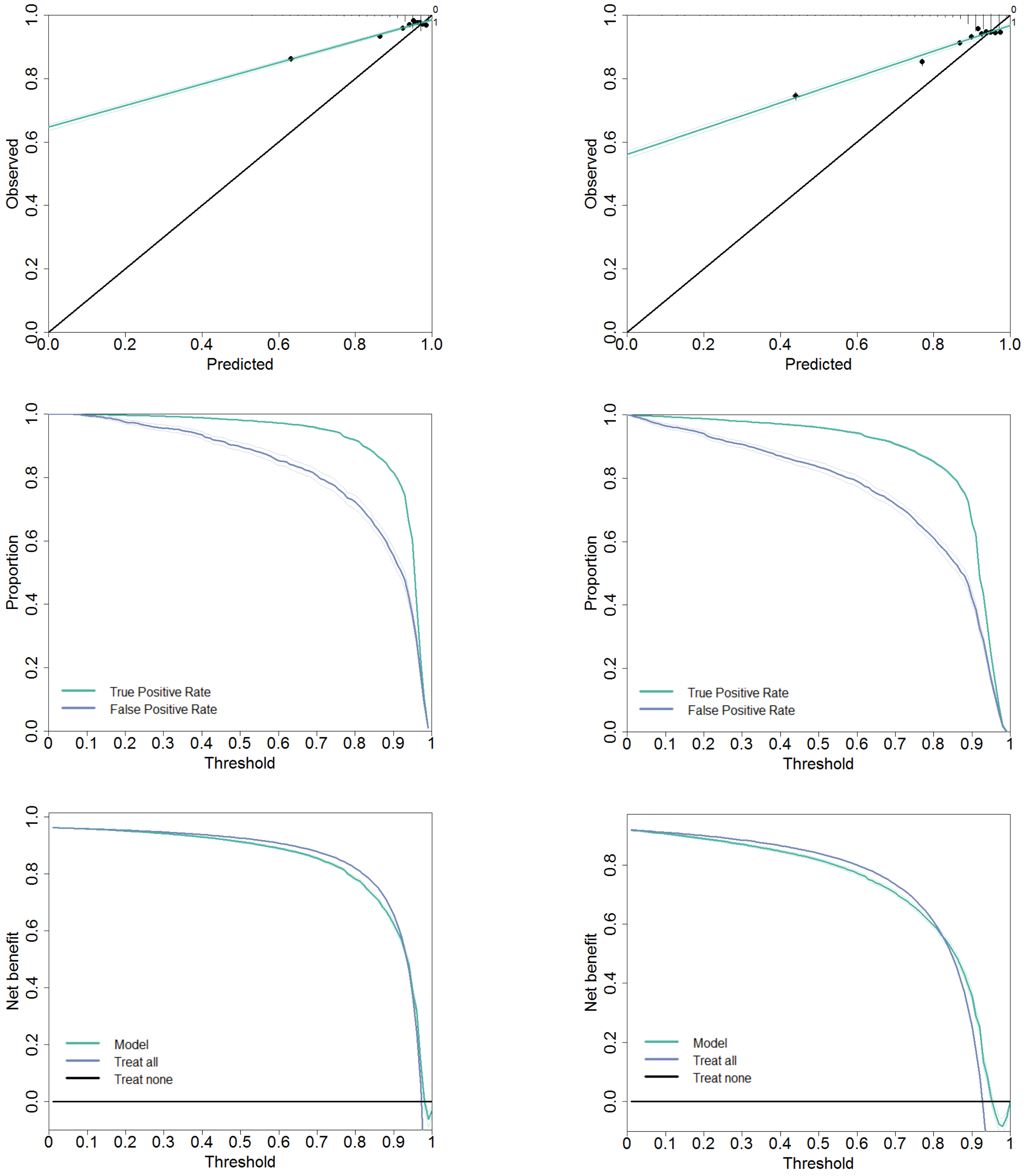

19. Chen et al. (Models 19a & 19b)

Reference: Chen, S., Liu, Y., Yang, J., Liu, Q., You, H., Dong, Y., & Lyu, J. (2019). Development and validation of a nomogram for predicting survival in male patients with breast cancer. *Frontiers in oncology*, *9*, 361.

Number of models presented in the paper: 2

Outcome:	3-year breast cancer specific survival<br>5-year breast cancer specific survival
Input variables:	Age (<65, ≥65)<br>Tumor Stage (I, II, III, IV)<br>ER status (Negative, Positive)<br>PR status (Negative, Positive)<br>Surgery (No, Yes)
Inclusion criteria:	Male patients diagnosed with breast cancer
Exclusion criteria:	Diagnosed at autopsy or by death certificate
Original validation:	3-year BCSS: 0.788<br>5-year BCSS: 0.825

Variable name	Input	3-year BCSS<br>N (%) total = 1330	5-year BCSS<br>N (%) total = 991
Age	<65	579 (43.5%)	475 (47.9%)
	≥65	751 (56.5%)	516 (52.1%)
Tumor stage	I	533 (40.1%)	394 (39.8%)
	II	485 (36.5%)	351 (35.4%)
	III	174 (13.1%)	136 (13.7%)
	IV	13 (1.0%)	12 (1.2%)
	Missing	125 (9.4%)	98 (9.9%)
ER status	Negative	31 (2.3%)	28 (2.8%)
	Positive	1152 (86.6%)	842 (85%)
	Missing	147 (11.1%)	121 (12.2%)
PR status	Negative	175 (13.2%)	134 (13.5%)
	Positive	985 (74.1%)	715 (72.1%)
	Missing	170 (12.8%)	142 (14.3%)
Surgery	No	126 (9.5%)	99 (10%)
	Yes	1204 (90.5%)	892 (90%)
BCSS	No	76 (5.7%)	103 (10.4%)
	Yes	1254 (94.3%)	888 (89.6%)

	3-year BCSS (19a)	5-year BCSS (19b)
AUC	0.827 (0.782 – 0.867)	0.789 (0.752 – 0.832)
Brier score	0.049 (0.040 – 0.061)	0.082 (0.070 – 0.095)
Scaled Brier score	0.078 (0.010 – 0.150)	0.112 (0.055 – 0.182)

3-year BCSS (19a)

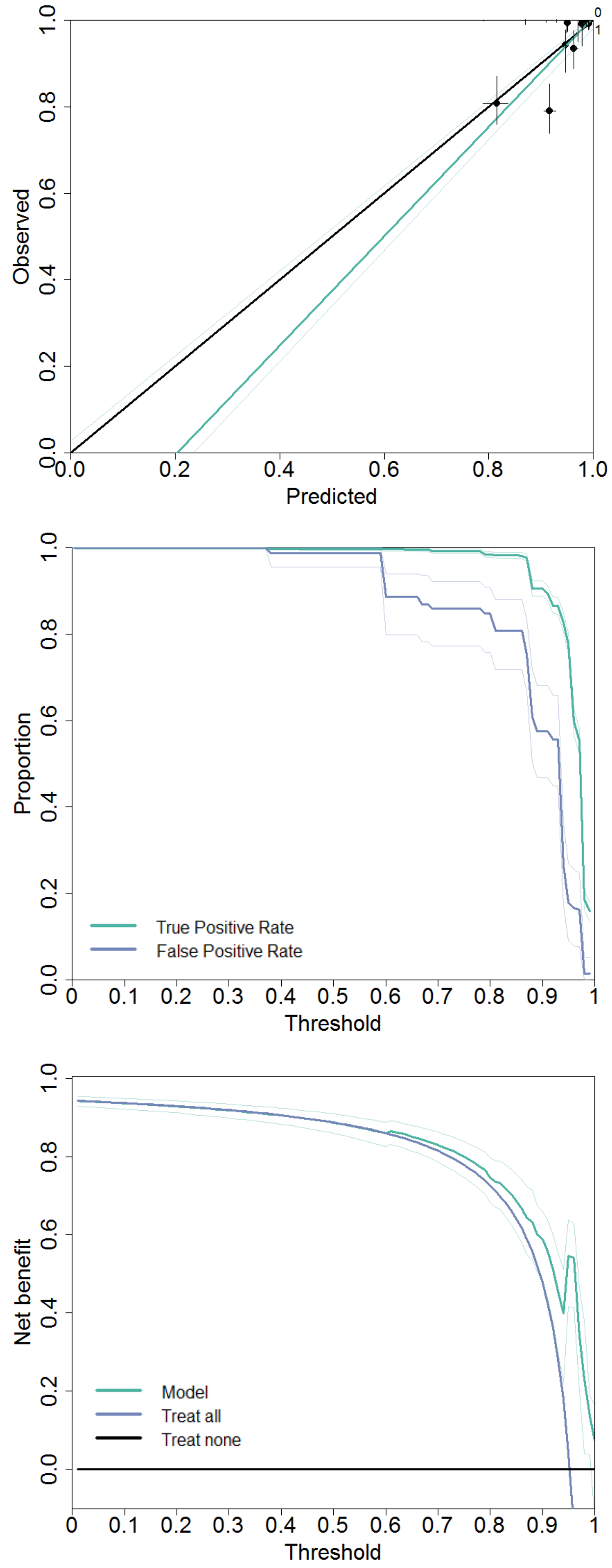

5-year BCSS (19b)

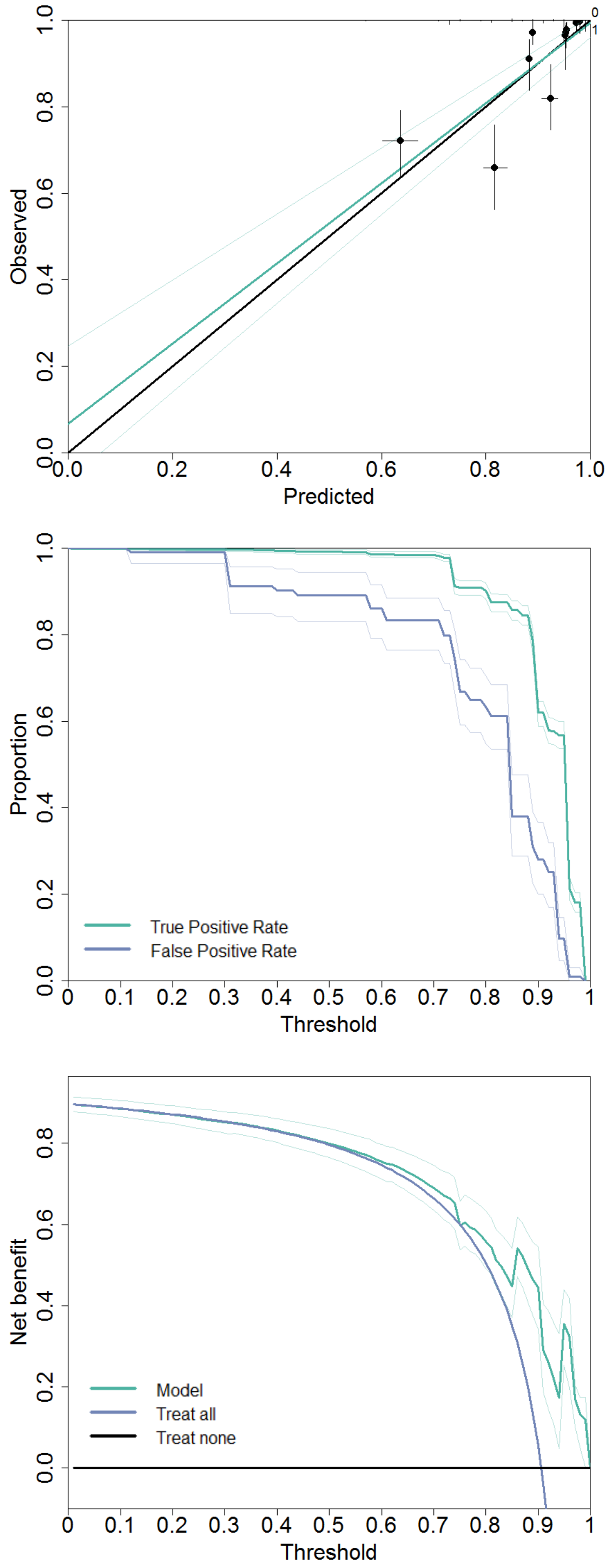

20. Fu et al. (Models 20a & 20b)

Reference: Fu, R., Yang, J., Wang, H., Li, L., Kang, Y., Kaaya, R. E., ... & Lyu, J. (2020). A nomogram for determining the disease-specific survival in invasive lobular carcinoma of the breast: A population study. *Medicine*, *99*(43).

Number of models presented in the paper: 2

Outcome:	3-year disease specific survival<br>5-year disease specific survival
Input variables:	Age (<40, 40 – 60 , 60 – 80, ≥80)<br>Site (Axillary tail, Central, Lower inner, Lower outer, Upper inner, Upper outer)<br>Grade (I, II, III)<br>Stage (II, III, IV)<br>Surgery (No, Yes)<br>Chemotherapy (No, Yes)<br>Molecular subtype (Luminal A, Luminal B, HER2 enriched, Triple negative)
Inclusion criteria:	Invasive Lobular Carcinoma, TNM stage II, III or IV
Exclusion criteria:	NA
Original validation:	3-year: 0.793 and 0.83 for training and validation cohorts, respectively<br>5-year: 0.772 and 0.824 for training and validation cohorts, respectively

Variable name	Input	3-year BCSS<br>N (%) total = 12246	5-year BCSS<br>N (%) total = 9849
Age	<40	274 (2.2%)	235 (2.4%)
	40 – 60	5041 (41.2%)	4293 (43.6%)
	60 – 80	5410 (44.2%)	4287 (43.5%)
	≥80	1521 (12.4%)	1034 (10.5%)
Site	Axillary tail	95 (0.8%)	80 (0.8%)
	Central	1850 (15.1%)	1468 (14.9%)
	Lower inner	817 (6.7%)	671 (6.8%)
	Lower outer	1327 (10.8%)	1048 (10.6)
	Upper inner	1569 (12.8%)	1243 (12.6%)
	Upper outer	6588 (52.8%)	5339 (54.2%)
Grade	I	1598 (13%)	1340 (13.6%)
	II	6969 (56.9%)	5417 (55.0%)
	III	1364 (11.1%)	1147 (11.6%)
	Missing	2315 (18.9%)	1945 (19.7%)
Stage	II	8396 (68.6%)	6659 (67.6%)
	III	2840 (23.2%)	2264 (23.0%)
	IV	1010 (8.2%)	926 (9.4%)
Surgery	No	1196 (9.8%)	888 (9.0%)
	Yes	11050 (90.2%)	8961 (91.0%)
Chemotherapy	No	6360 (51.9%)	4814 (48.9%)
	Yes	5886 (48.1%)	5035 (51.1%)
Molecular subtype	Luminal A	9496 (77.5%)	7472 (75.9%)
	Luminal B	420 (3.4%)	340 (3.5%)
	HER2 enriched	111 (0.9%)	92 (0.9%)
	Triple negative	255 (2.1%)	208 (2.1%)
	Missing	1964 (16%)	1737 (17.6%)
BCSS	No	717 (5.9%)	1080 (11%)
	Yes	11529 (94.1%)	8769 (89%)

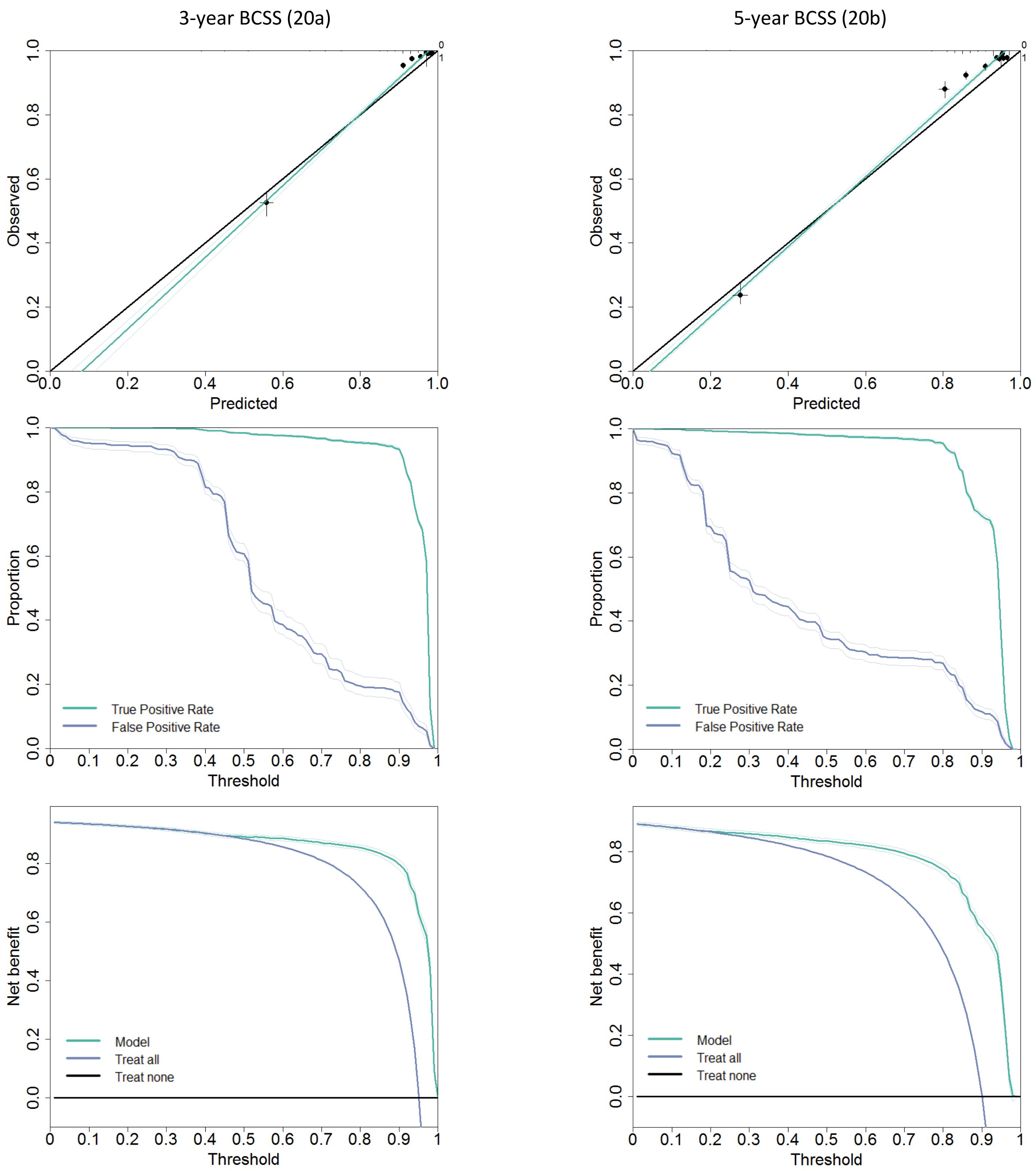

	3-year BCSS (20a)	5-year BCSS (20b)
AUC	0.926 (0.911 – 0.936)	0.900 (0.889 – 0.912)
Brier score	0.034 (0.031 – 0.036)	0.049 (0.047 – 0.052)
Scaled Brier score	0.389 (0.358 – 0.419)	0.491 (0.466 – 0.518)

21. Herrero Vicent et al. (Model 21)

Reference: Herrero-Vicent, C., Guerrero-Zotano, A., *Gavilá-Gregori, J., Hernández-Blanquisett, A., Sandiego-Contreras, S., Samper-Hiraldo, J. M., ... & Ruiz-Simón, A. (2016). A prognostic index for locoregional recurrence after neoadjuvant chemotherapy. ecancermedicalscience, 10.*

Number of models presented in the paper: 1

Outcome:	6-year locoregional recurrence
Input variables:	Point score with 1 point for HER2 positive, DCIS, age <40, and No-PCR (if patient underwent mastectomy).
Inclusion criteria:	Histologically confirmed breast cancer treated with neoadjuvant chemotherapy.
Exclusion criteria:	No follow-up
Original validation:	Not assessed

Variable name	Input	N (%) total = 739
Age	<40	623 (84.3%)
	≥40	116 (15.7%)
HER2-status	Negative	502 (67.9%)
	Positive	174 (23.6%)
	Missing	63 (8.5)
DCIS	No	714 (96.6%)
	Yes	63 (3.4%)
PCR	No	661 (89.4%)
	Yes	78 (10.6%)
6-year LRR	No	566 (76.6%)
	Yes	173 (23.4%)

AUC	0.583 (0.544 – 0.617)
Brier score	0.201 (0.178 – 0.227)
Scaled Brier score	-0.124 (-0.170 – -0.088)

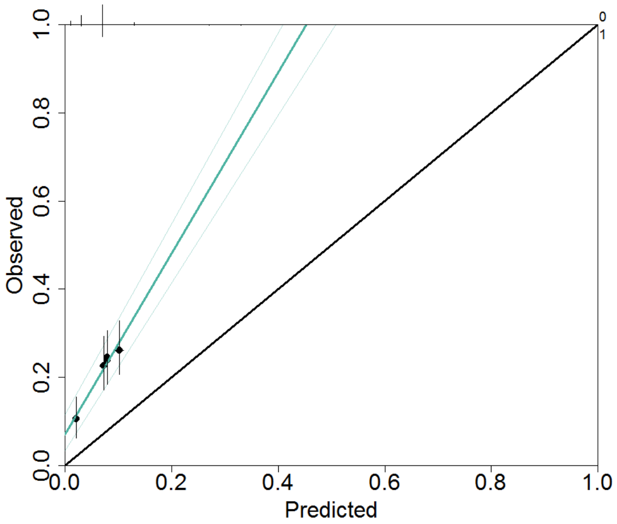

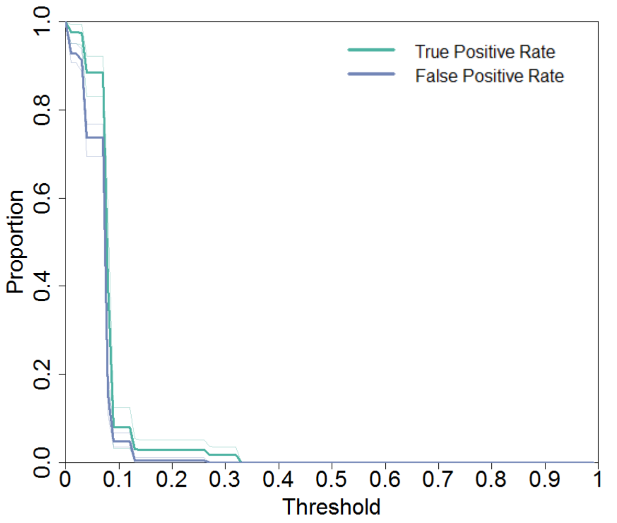

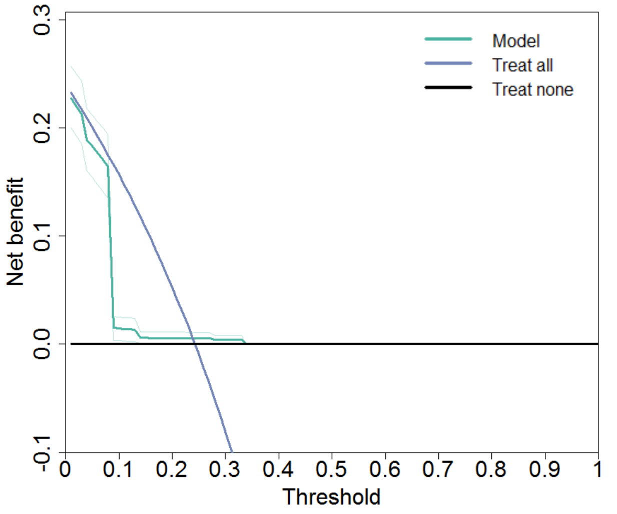

22. Wobb et al. (Model 22)

Reference: Wobb, J. L., Chen, P. Y., Shah, C., Moran, M. S., Shaitelman, S. F., Vicini, F. A., ... & Beitsch, P. (2015). Nomogram for predicting the risk of locoregional recurrence in patients treated with accelerated partial-breast irradiation. *International Journal of Radiation Oncology* Biology* Physics*, *91*(2), 312-318.

Number of models presented in the paper: 1

Outcome:	5-year locoregional recurrence
Input variables:	Age (<50, ≥50)<br>Menopausal status (Post, pre/peri)<br>Margin (negative, positive/close)<br>ER status (positive, negative)<br>Tumor grade (I, II/III)
Inclusion criteria:	Patients treated with a lumpectomy followed by accelerated partial breast irradiation
Exclusion criteria:	NA
Original validation:	0.641

Variable name	Input	N (%) total = 11822
Age	<50	3460 (29.3%)
	≥50	8362 (70.7%)
	Years (mean (sd))	56 (11)
Menopausal status	Pre	1550 (13.1%)
	Peri	307 (2.6%)
	Post	3307 (28%)
	Missing	6658 (56.3%)
Margin	Negative	10466 (88.5%)
	Positive/close	1356 (11.5%)
ER status	Negative	2375 (20.1%)
	Positive	9264 (78.4%)
	Missing	183 (1.5%)
Tumor grade	I	2712 (22.9%)
	II	4547 (38.5%)
	III	3621 (30.6%)
	Missing	942 (8%)
5-year locoregional recurrence	No	11553 (97.7%)
	Yes	269 (2.3%)

C-index	0.478 (0.448 – 0.565)
Brier score	0.067 (0.065 – 0.068)
Scaled Brier score	-1.996 (-2.235 – -1.767

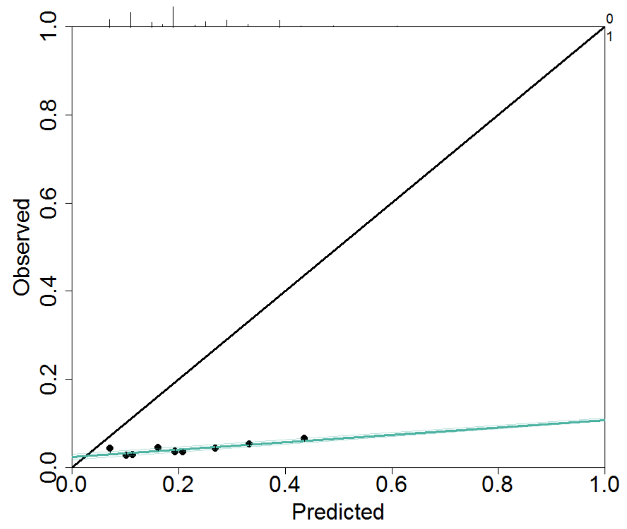

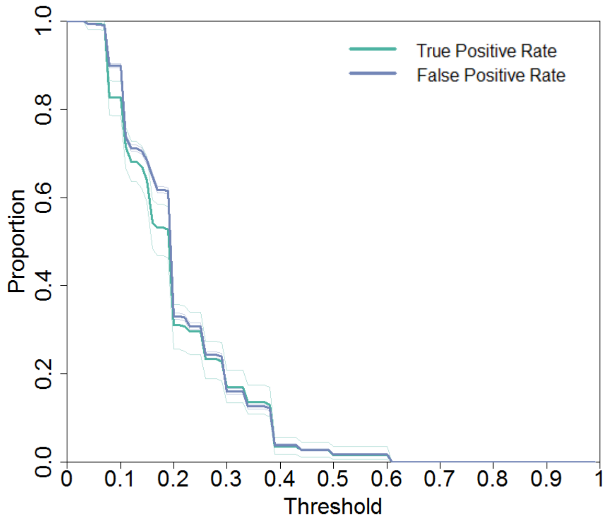

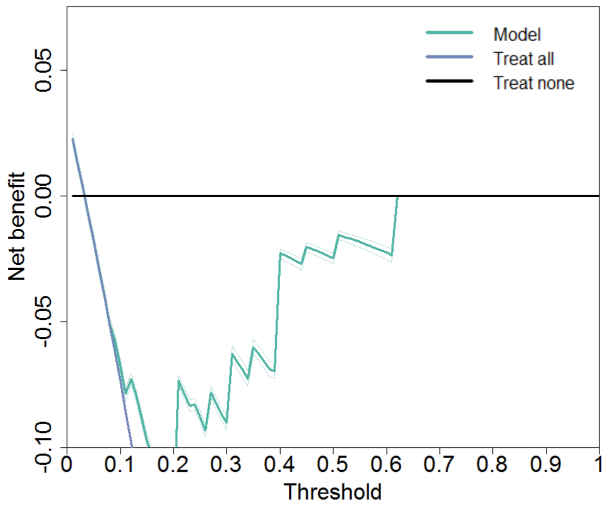

23. Sanghani et al. (Model 23)

Reference: Sanghani, M., Truong, P. T., Abi Raad, R., Niemierko, A., Lesperance, M., Olivotto, I. A., ... & Taghian, A. G. (2010). Validation of a web-based predictive nomogram for ipsilateral breast tumor recurrence after breast conserving therapy. *Journal of clinical oncology*, *28*(5), 718.

Outcome:	10-year ipsilateral (local) relapse
Input variables:	Age (≤40, 40–45, 45–50, 55–60, 60–65, >70)<br>LVI (No, Yes, Unknown)<br>Margin (Positive, Close, Negative, Unknown)<br>Size (≤1cm, 1 – 2 cm, >2 cm)<br>Grade (Low, Intermediate, High, Unknown)<br>Chemotherapy (with, without)<br>Hormonal therapy (with, without)
Inclusion criteria:	Invasive disease, pT1-3, M0, BCS
Exclusion criteria:	NA
Original validation:	0.66

Variable name	Input	N (%) total = 7343
Age	≤40	601 (8.2%)
	40 – 45	780 (10.6%)
	45 – 50	1248 (17%)
	50 – 55	1327 (18.1%)
	55 – 60	1293 (17.6%)
	60 – 65	944 (12.9%)
	65 – 70	696 (9.5%)
	>70	454 (6.2%)
Size	≤1 cm	1620 (22.1%)
	1 – 2 cm	3683 (50.2%)
	>2 cm	1713 (23.3%)
	Missing	327 (4.5%)
Grade	Low	1914 (26.1%)
	Intermediate	2874 (39.1%)
	High	2006 (27.3%)
	Missing	548 (7.5%)
Margin	Negative	4716 (64.2%)
	Close	154 (2.1%)
	Positive	35 (0.5%)
	Missing	2438 (33.2%)
Chemotherapy	No	4655 (63.4%)
	Yes	2688 (36.6%)
Hormonal therapy	No	4696 (64%)
	Yes	2647 (36%)
Radiotherapy	No	89 (1.2%)
	Yes	7254 (98.8%)
10-year relapse	No	6842 (93.2%)
	Yes	501 (6.8%)

AUC	0.592 (0.566 – 0.617)
Brier score	0.063 (0.059 – 0.069)
Scaled Brier score	0.006 (-0.003 – 0.016)

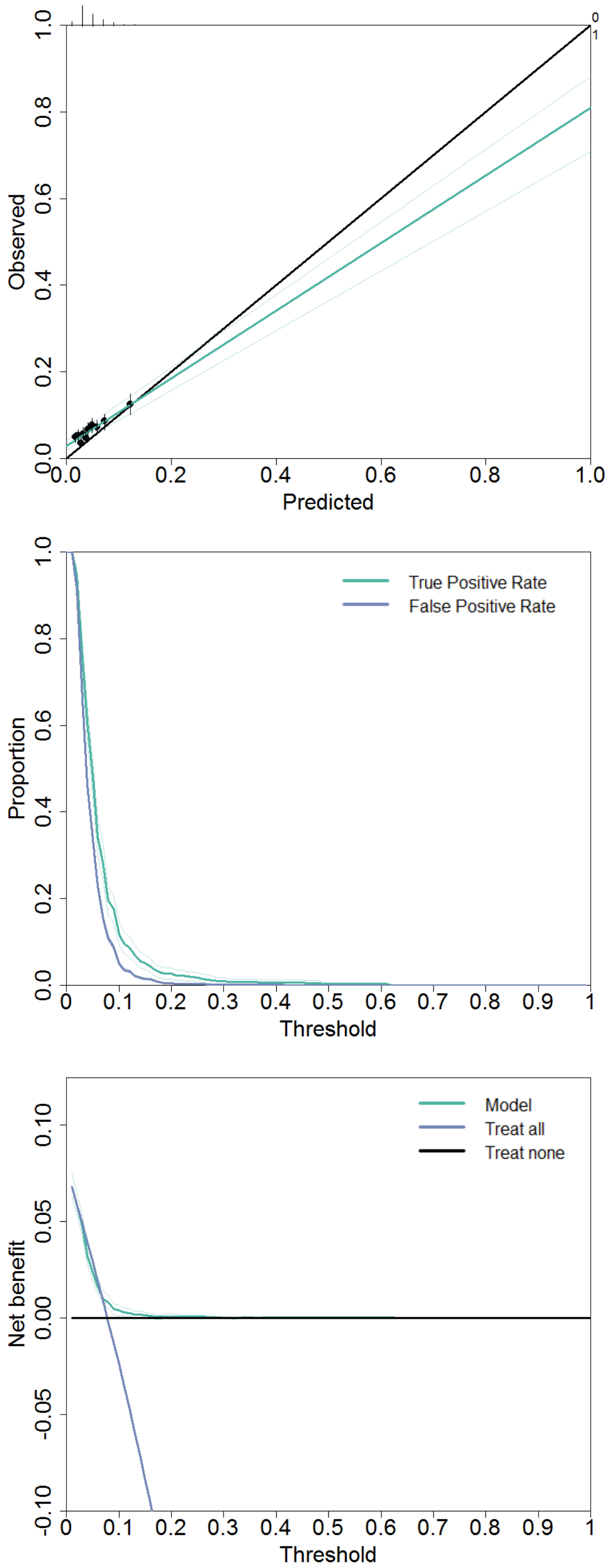

24. Li et al. (Model 24)

Reference: Li, M., Yue, J., Wan, X., Hua, B., Yang, Q., Yang, P., ... & Xia, X. (2020). Risk-Adapted Postmastectomy Radiotherapy Decision Based on Prognostic Nomogram for pT1-2N1M0 Breast Cancer: A Multicenter Study. *Frontiers in Oncology*, *10*.

Number of models presented in the paper: 1

Outcome:	5-year locoregional recurrence (free survival)
Input variables:	Age (>45, ≤45)<br>Topography (lateral region, inner/central)<br>Positive lymph nodes (1, 2, 3)<br>Pathological T stage (pT1, pT2)<br>Molecular subtype (Lum A, Lum B, HER2+, TNBC)
Inclusion criteria:	Female breast cancer patients, T1, T2 disease and 1-3 positive lymph nodes, treated with mastectomy followed by adjuvant systemic therapy.
Exclusion criteria:	Bilateral breast cancer, other (previous) malignancies, metastatic disease
Original validation:	0.735 and 0.703 on internal and external validation cohorts, respectively

Variable name	Input	N (%) total = 2886
Age	>45	1969 (68.2%)
	≤45	917 (31.8%)
Topography	Lateral	1285 (44.5%)
	Inner	396 (13.7%)
	Central	247 (8.6%)
	Other	958 (33.2%)
Positive lymph nodes	1	1486 (51.5%)
	2	903 (31.3%)
	3	497 (17.2%)
T stage	pT1	1119 (38.8%)
	pT2	1767 (61.2%)
Molecular subtype	Luminal A	1389 (48.1%)
	Luminal B	324 (11.2%)
	HER2+	253 (8.8%)
	TNBC	332 (11.5%)
	Missing	588 (20.4%)
Locoregional recurrence	No	2799 (97%)
	Yes	87 (3.0%)

AUC	0.619 (0.558 – 0.682)
Brier score	0.039 (0.034 – 0.043)
Scaled Brier score	-0.309 (-0.435 – -0.216)

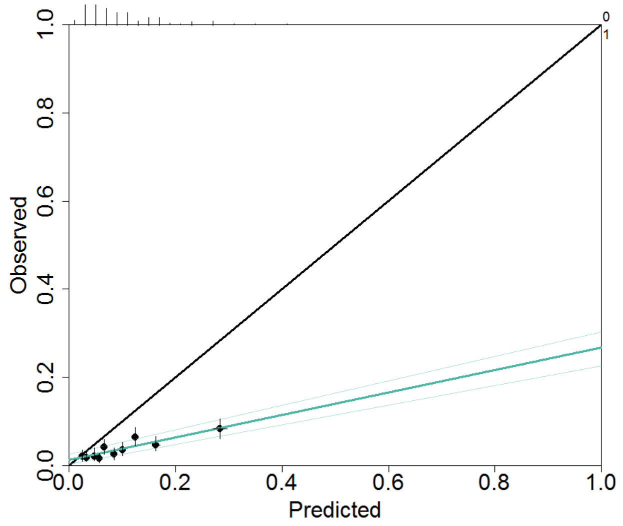

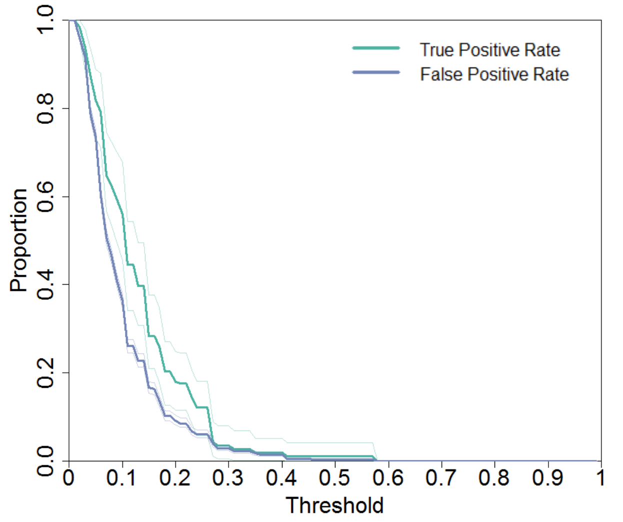

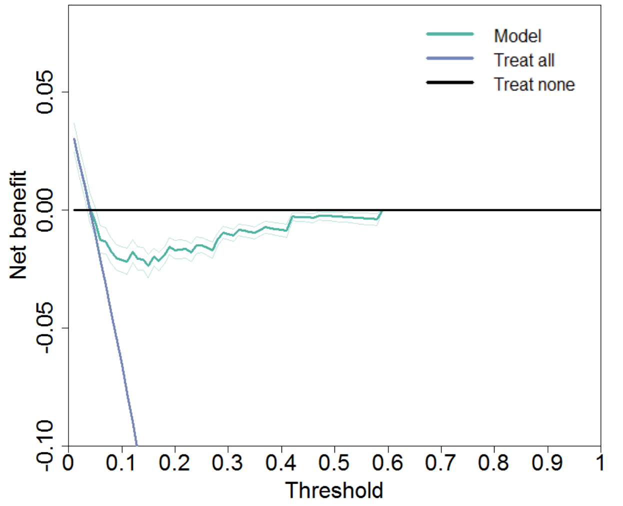

25. Corso et al. (Models 25a, 25b, 25c)

Reference: Corso, G., Maisonneuve, P., Massari, G., Invento, A., Pravettoni, G., De Scalzi, A., ... & Veronesi, P. (2020). Validation of a novel Nomogram for prediction of local relapse after surgery for invasive breast carcinoma. *Annals of surgical oncology*, 1-11.

Number of models presented in the paper: 6 models presented, 3 could be validated

Outcome:	1-year, 5-year, and 10-year ipsilateral local recurrence
Input variables:	Age (<35, 35-49, ≥50)<br>Histology (Lobular, Ductal, Mixed, Other)<br>Tumor stage (T1, T2, T3, T4)<br>Nodal stage (N0, 1-3 nodes, >3 nodes)<br>Molecular subtype (Luminal A, Luminal B, HER2+, TNBC)<br>Hormone therapy (no, yes)<br>Chemotherapy (no, yes)<br>Radiotherapy (none, intraoperative, external)
Inclusion criteria:	First primary invasive breast cancer, underwent mastectomy
Exclusion criteria:	Neoadjuvant treatment, metastatic disease
Original validation:	0.70 (95% CI: 0.56 – 0.82)

Variable	Input	1-year N = 22882	5-year N = 18498	10-year N = 15173
Age	<35	520 (2.3%)	434 (2.3%)	416 (2.7%)
	35-49	5086 (22.2%)	4478 (24.2%)	4175 (27.5%)
	≥50	17276 (75.5%)	13586 (73.4%)	10582 (69.7%)
Histology	Lobular	3414 (14.9%)	2790 (15.1%)	2259 (14.9%)
	Ductal	16094 (70.3%)	12931 (69.9%)	10672 (70.3%)
	Mixed	1630 (7.1%)	1373 (7.4%)	1118 (7.4%)
	Other	1744 (7.6%)	1404 (7.6%)	1124 (7.4%)
T stage	T1	10471 (45.8%)	9128 (49.3%)	7798 (51.4%)
	T2	10682 (46.7%)	8181 (44.2%)	6459 (42.6%)
	T3	1362 (6%)	978 (5.3%)	791 (5.2%)
	T4	367 (1.6%)	211 (1.1%)	125 (0.8%)
N stage	N0	11905 (52%)	10120 (54.7%)	8356 (55.1%)
	1-3 nodes	7175 (31.4%)	5860 (31.7%)	4870 (32.1%)
	>3 nodes	3774 (16.5%)	2506 (13.5%)	1938 (12.8%)
Molecular subtype	Luminal A	10680 (46.7%)	8935 (48.3%)	7326 (48.3%)
	Luminal B	803 (3.5%)	611 (3.3%)	478 (3.2%)
	HER2+	540 (2.4%)	361 (2%)	300 (2%)
	TNBC	1873 (8.2%)	1388 (7.5%)	1217 (8%)
Hormone therapy	No	10471 (45.8%)	8406 (45.4%)	7034 (46.4%)
	Yes	12411 (54.2%)	10092 (54.6%)	8139 (53.6%)
Chemotherapy	No	14071 (61.5%)	11016 (59.6%)	8376 (55.2%)
	Yes	8811 (38.5%)	7482 (40.4%)	6797 (44.8%)
Radiotherapy	No	17229 (75.3%)	14358 (77.6%)	11820 (77.9%)
	Yes	5653 (24.7%)	4140 (22.4%)	3353 (22.1%)
Recurrence	No	22731 (99.3%)	17752 (96%)	14324 (94.4%)
	Yes	151 (0.7%)	746 (4%)	849 (5.6%)

	1-year (25a)	5-year (25b)	10-year (25c)
AUC	0.765 (0.728 – 0.801)	0.689 (0.668 – 0.708)	0.679 (0.661 – 0.697)
Brier score	0.006 (0.006 – 0.007)	0.037 (0.035 – 0.039)	0.051 (0.048 – 0.054)
Scaled Brier score	0.016 (0.007 – 0.024)	0.037 (0.029 – 0.045)	0.038 (0.028 – 0.048)

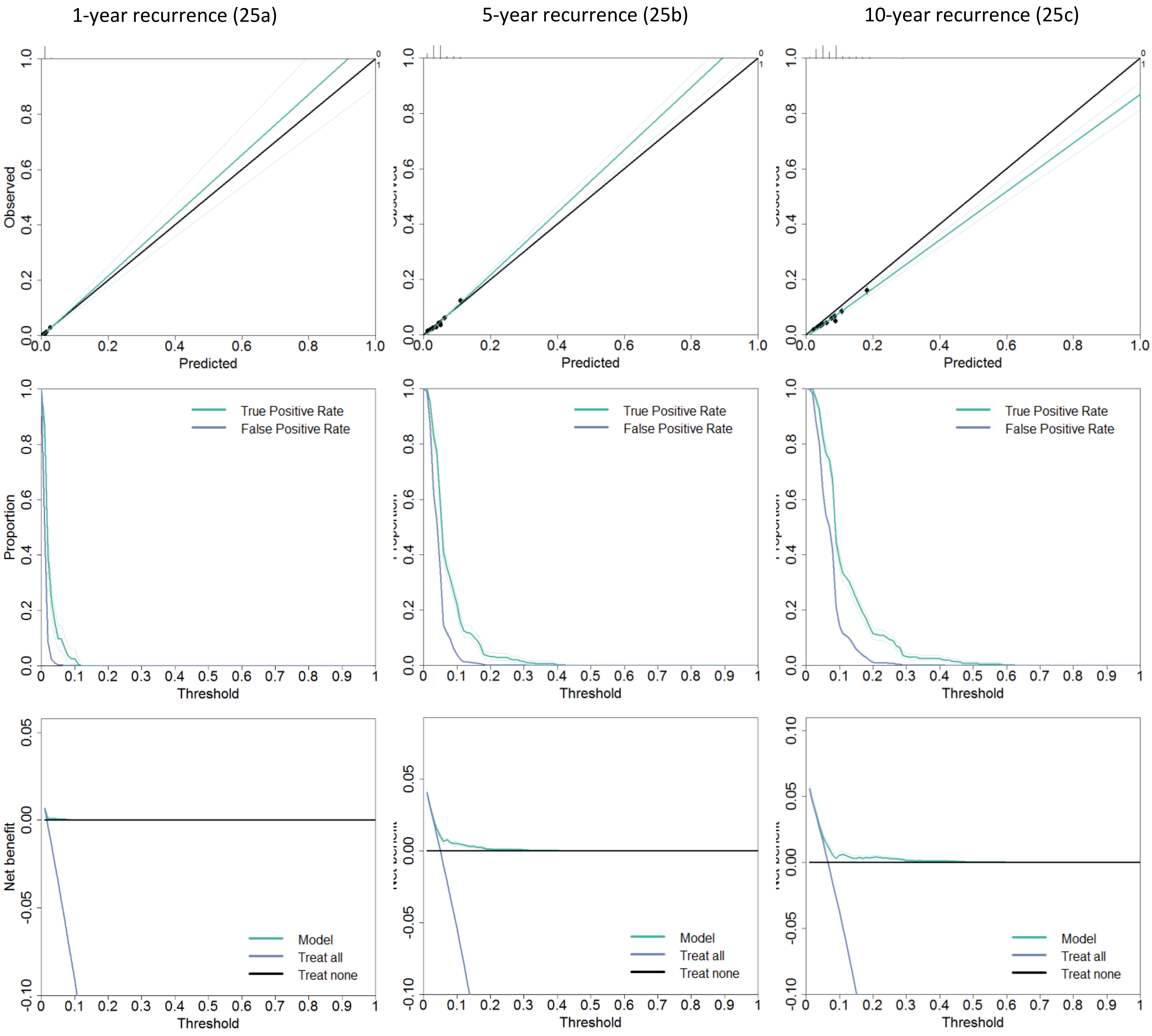
1-year recurrence (25a)
5-year recurrence (25b)
10-year recurrence (25c)
Observed
Predicted
Proportion
True Positive Rate
False Positive Rate
Threshold
Net benefit
Model
Treat all
Treat none

26. Li et al. (Model 26)

Reference: Li, S., Yu, K. D., Fan, L., Hou, Y. F., & Shao, Z. M. (2011). Predicting breast cancer recurrence following breast-conserving therapy: a single-institution analysis consisting of 764 Chinese breast cancer cases. *Annals of surgical oncology*, *18*(9), 2492-2499.

Number of models presented in the paper: 1

Outcome:	5-year recurrence survival
Input variables:	Molecular subtype (Luminal A, non-Luminal A)<br>Grade (I, II, III)<br>Positive lymph nodes (0, 1-3, ≥4)
Inclusion criteria:	Women diagnosed with invasive breast cancer, undergoing breast conserving surgery.
Exclusion criteria:	NA
Original validation:	0.70 (95%CI: 0.61 – 0. 78)

Variable name	Input	N (%) total = 44176
Molecular subtype	Luminal A	21646 (49%)
	Non-Luminal A	15896 (36%)
	Missing	5783 (13.1%)
Tumor grade	I	9958 (22.5%)
	II	17485 (39.6%)
	III	12859 (29.1%)
	Missing	3023 (6.8%)
Positive lymph nodes	0	29197 (66.1%)
	1 – 3	10932 (24.7%)
	≥4	3196 (7.2%)
5-year recurrence	No	33115 (75%)
	Yes	10210 (23.1%)

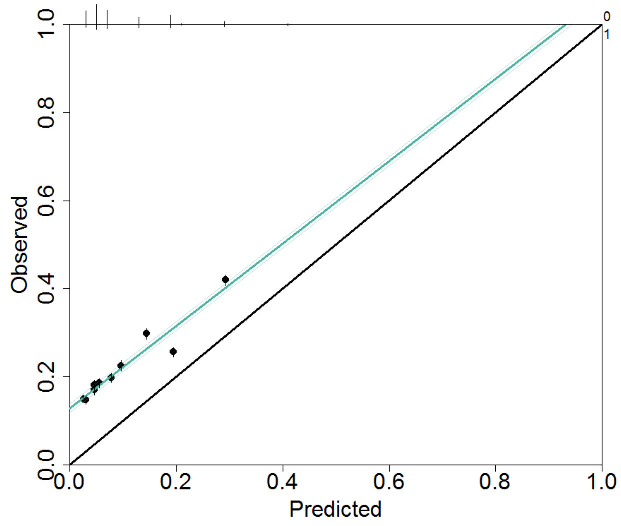

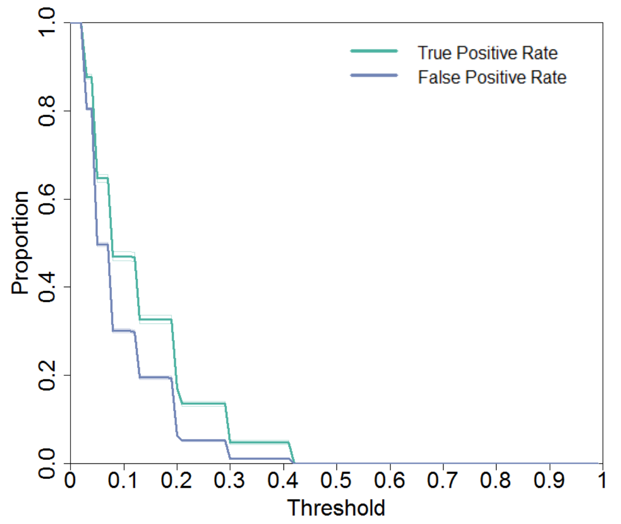

AUC	0.610 (0.604 – 0.616)
Brier score	0.192 (0.189 – 0.195)
Scaled Brier score	-0.066 (-0.073 – -0.060)

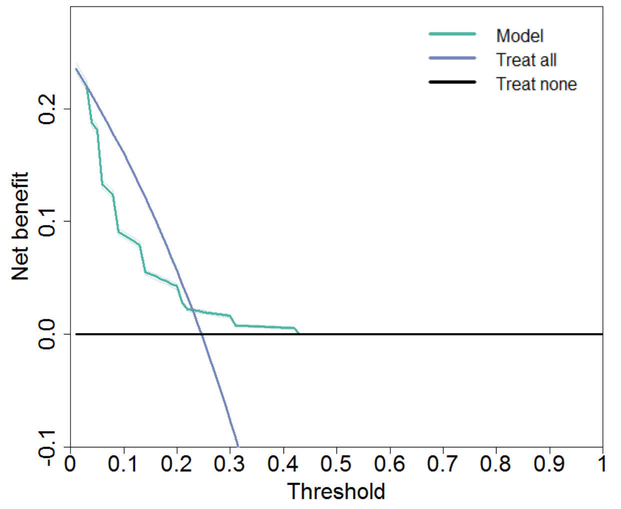

27. Tokatli et al. (Model 27)

Reference: Tokatli, Z. F., Türe, M., Ömürlü, İ. K., Alas, R. Ç., & Uzal, M. C. (2011). Developing and comparing two different prognostic indexes for predicting disease-free survival of nonmetastatic breast cancer patients. Turkish Journal of Medical Sciences, 41(5), 769-780.

Number of models presented in the paper: 1

Outcome:	5-year Disease free survival
Input variables:	Axillary nodal status (negative, positive)<br>HER2/neu (negative, positive)<br>Estrogen receptor status (negative, positive)
Inclusion criteria:	Non-metastatic breast cancer.
Exclusion criteria:	NA
Original validation:	0.70 & 0.715 in the training and validation set, respectively

Variable name	Input	N (%) total = 58568
Lymph node status	Negative	36983 (63.1%)
	Positive	21585 (36.9%)
HER2	Negative	32419 (55.4%)
	Positive	6182 (10.6%)
	Missing	19967 (34.1%)
ER	Negative	11390 (19.4%)
	Positive	43863 (74.9%)
	Missing	3315 (5.7%)
5-year disease free survival	No	7350 (12.5%)
	Yes	51218 (87.5%)

AUC	0.633 (0.626 – 0.638)
Brier score	0.129 (0.128 – 0.131)
Scaled Brier score	-0.180 (-0.193 – -0.166)

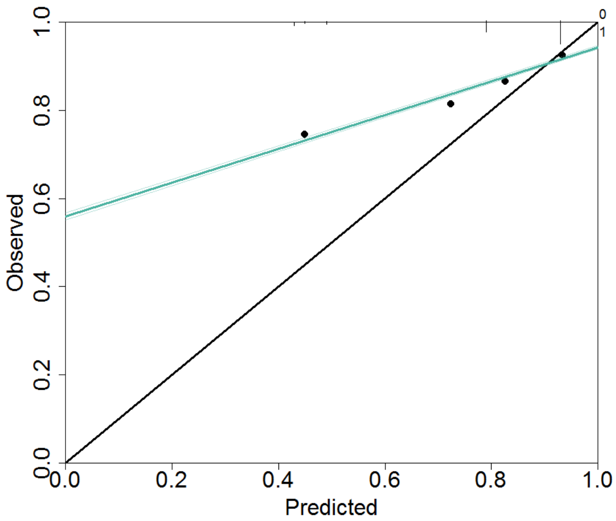

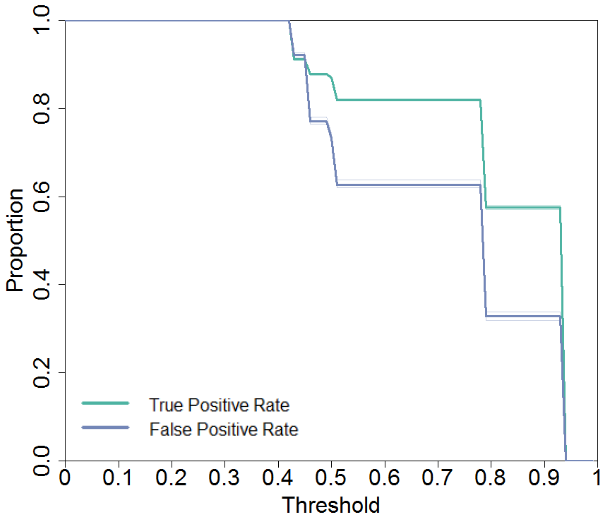

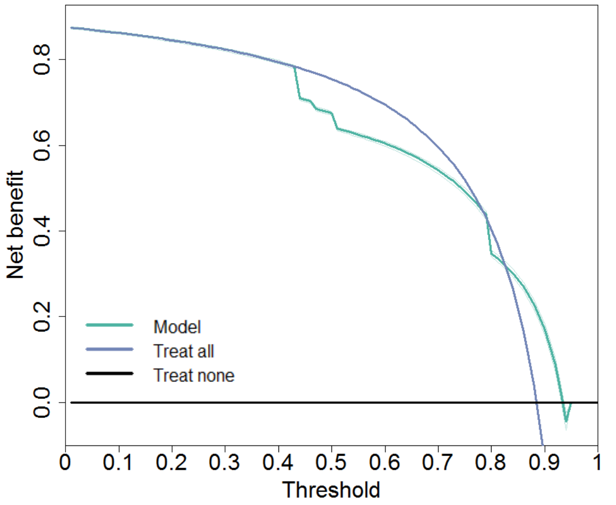

28. Lin et al. (Models 28a, 28b, 28c)

Reference: *Lin, H., Zhang, F., Wang, L., & Zeng, D. (2019). Use of clinical nomograms for predicting survival outcomes in young women with breast cancer. Oncology letters, 17(2), 1505-1516.*
Number of models presented in the paper: 9 models presented. 3 models could not be validated, 3 models had insufficient sample size.

Outcome:	1-year Visceral metastasis, Disease free survival, and Overall survival<br>2-year Visceral metastasis, Disease free survival, and Overall survival<br>3-year Visceral metastasis, Disease free survival, and Overall survival
Input variables:	<u>Visceral metastasis:</u><br>N-stage (N0, N1, N2, N3)<br>Molecular subtype (Luminal A, Luminal B, Triple negative, HER2-positive)<br>Age (<35, ≥35)<br><br><u>Disease free survival</u><br>N-stage (N0, N1, N2, N3)<br>Molecular subtype (Luminal A, Luminal B, Triple negative, HER2-positive)
Inclusion criteria:	≤40 years, female, breast cancer confirmed by pathologic diagnosis
Exclusion criteria:	Distant metastasis at diagnosis, follow-up <6 months
Original validation:	Not provided

Variable name	Input	1-year N = 5127	2-year N = 4919	3-year N = 4759
Age	<35	1594 (31.1%)	1514 (30.8%)	1461 (30.7%)
	≥35	3533 (68.9%)	3405 (69.2%)	3298 (69.3%)
	Years (mean (sd))	35.8 (3.8)	35.8 (3.8)	35.8 (3.8)
N-stage	N0	2565 (50%)	2485 (50.5%)	2421 (50.9%)
	N1	1680 (32.8%)	1611 (32.8%)	1558 (32.7%)
	N2	586 (11.4%)	553 (11.2%)	528 (11.1%)
	N3	296 (5.8%)	270 (5.5%)	252 (5.3%)
Molecular subtype	Luminal A	1677 (32.7%)	1643 (33.4%)	1586 (33.3%)
	Luminal B	508 (9.9%)	502 (10.2%)	492 (10.3%)
	HER2+	347 (6.8%)	338 (6.9%)	322 (6.8%)
	TNBC	1098 (21.4%)	952 (19.4%)	883 (18.6%)
	Missing	1497 (29.2%)	1484 (30.2%)	1476 (31%)
Disease free survival	No	123 (2.4%)	409 (8.3%)	593 (12.5%)
	Yes	5004 (97.6%)	4510 (91.7%)	4166 (87.5%)

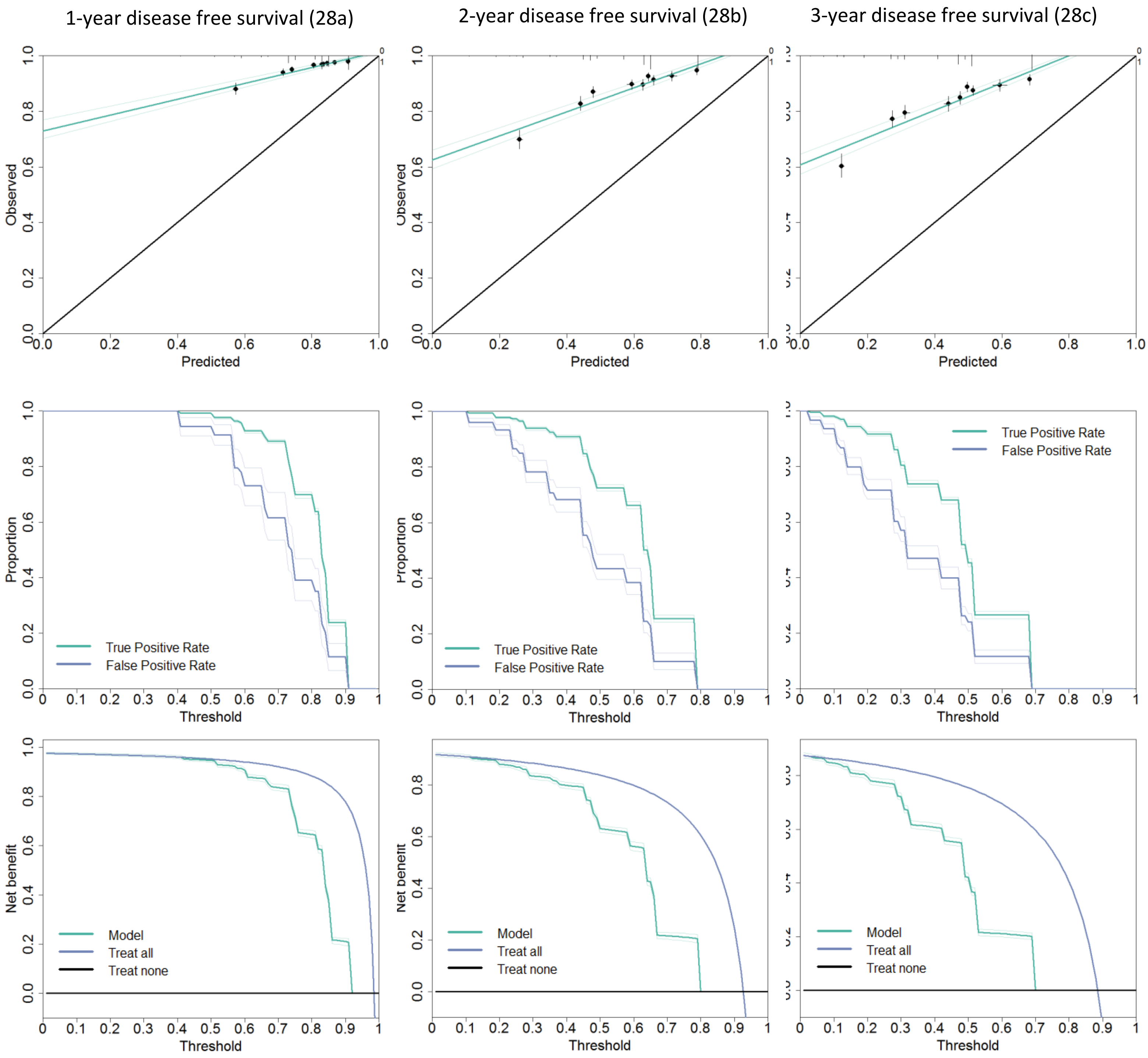

Disease free survival	1-year (28a)	2-year (28b)	3-year (28c)
AUC	0.692 (0.647 – 0.738)	0.693 (0.665 – 0.722)	0.684 (0.660 – 0.710)
Brier score	0.059 (0.056 – 0.061)	0.183 (0.179 – 0.187)	0.284 (0.279 – 0.290)
Scaled brier score	-1.488 (-1.839 – -1.218)	-1.406 (-1.633 – -1.225)	-1.608 (-1.811 – -1.442)

29. Paredes Aracil et al. (Models 29a & 29b)

Reference: *Paredes-Aracil, E., Palazón-Bru, A., Folgado-de la Rosa, D. M., Ots-Gutiérrez, J. R., Llorca-Ferrándiz, C., Alonso-Hernández, S., ... & Gil-Guillén, V. F. (2018). A scoring system to predict recurrence in breast cancer patients. Surgical oncology, 27(4), 681-687.*

Number of models presented in the paper: 2 models

Outcome:	5-year recurrent disease<br>10-year recurrent disease
Input variables:	Age (<80, ≥80 years)<br>Stage (0, I, IIA, IIB, IIIA, IIIB, IIIC)<br>Multicentricity (No, Yes)<br>Grade (Low, Moderate, High)
Inclusion criteria:	Breast cancer without distant metastasis
Exclusion criteria:	Stage IV breast cancer, Positive surgical margins
Original validation:	0.75

Variable name	Input	5-year recurrence<br>N (%) total = 21653	10-year recurrence<br>N (%) total = 7750
Stage	0	292 (1.3%)	31 (0.4%)
	I	6791 (31.4%)	3254 (42%)
	IA	2026 (9.4%)	0 (0%)
	IB	207 (1%)	0 (0%)
	IIA	6657 (30.7%)	2326 (30%)
	IIB	2799 (12.9%)	1032 (13.3%)
	IIIA	1808 (8.3%)	685 (8.8%)
	IIIB	204 (0.9%)	63 (0.8%)
	IIIC	869 (4%)	359 (4.6%)
Age	<80	20469 (94.5%)	7595 (98%)
	≥80	1184 (5.5%)	155 (2%)
	Years (mean (sd))	57 (13)	55 (11)
Multicentricity	No	17975 (83%)	6492 (83.8%)
	Yes	3016 (13.9%)	889 (11.5%)
	Missing	662 (3.1%)	369 (4.8%)
Grade	Low	3974 (18.4%)	1474 (19%)
	Moderate	8247 (38.1%)	2993 (38.6%)
	High	7234 (33.4%)	2578 (33.3%)
	Missing	2198 (10.2%)	705 (9.1%)
Recurrence	No	19064 (88%)	5776 (74.5%)
	Yes	2589 (12%)	1974 (25.5%)

5-year recurrence (29a)

10-year recurrence (29b)

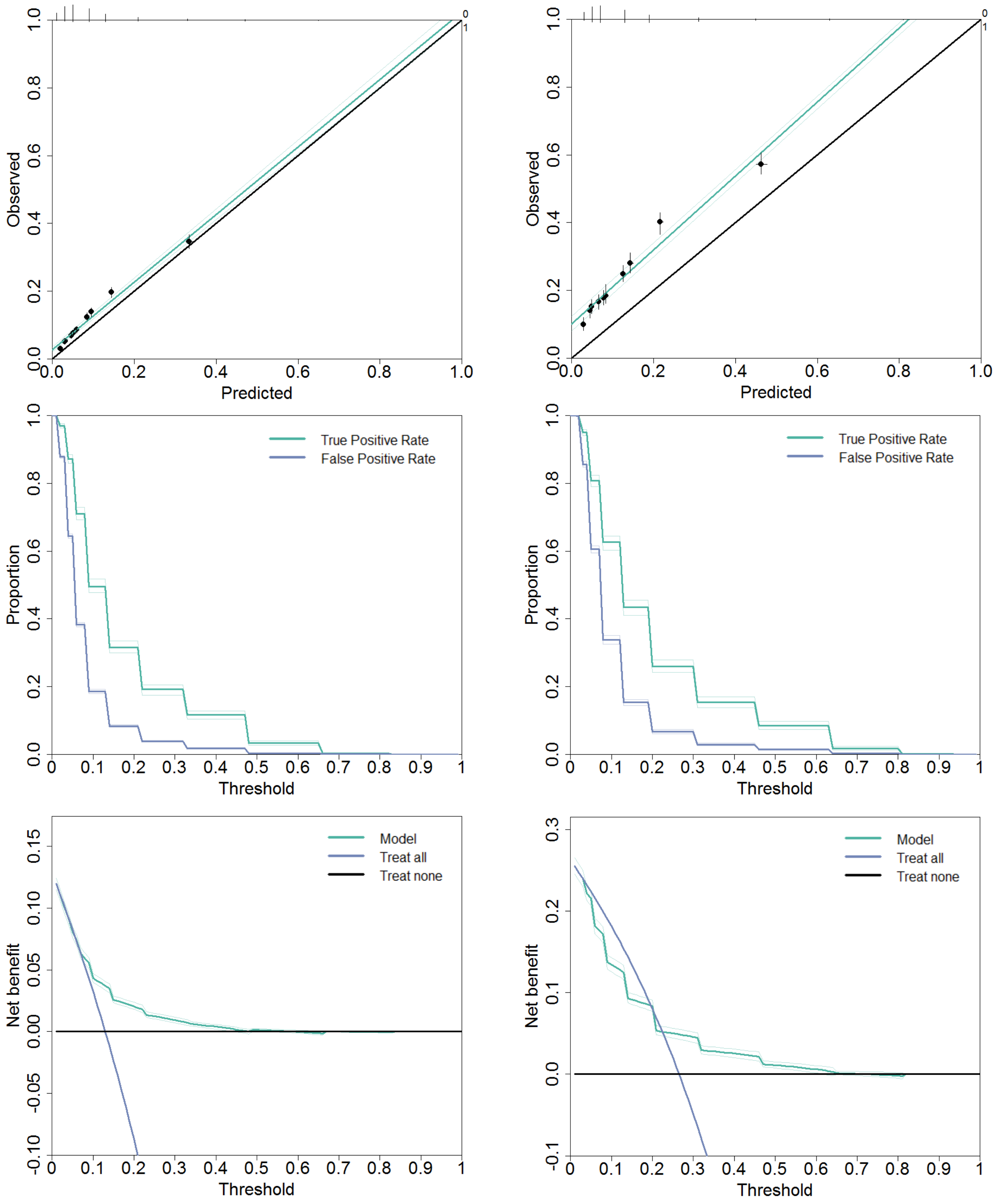

Recurrence	5-year recurrence (29a)	10-year recurrence (29b)
AUC	0.718 (0.707 – 0.729)	0.692 (0.678 – 0.705)
Brier score	0.098 (0.094 – 0.101)	0.187 (0.180 – 0.193)
Scaled Brier score	0.073 (0.063 – 0.085)	0.018 (0.001 – 0.034)

30. Dowsett et al. (Model 30)

Reference: Dowsett, M., Sestak, I., Regan, M. M., Dodson, A., Viale, G., Thürlimann, B., ... & Cuzick, J. (2018). Integration of clinical variables for the prediction of late distant recurrence in patients with estrogen receptor–positive breast cancer treated with 5 years of endocrine therapy: CTS5. Journal of Clinical Oncology, 36(19), 1941.

Number of models presented in the paper: 1

Outcome:	5 to 10 years distant recurrence
Input variables:	Tumor size (Continuous)<br>Lymph nodes positive (Continuous)<br>Age (Continuous)<br>Tumor grade (I, II, III)
Inclusion criteria:	ATAC trial (ISRCTN18233230)<br>Post-menopausal, ER positive, anastrozole alone or tamoxifen alone, distant recurrence free after 5 years of follow-up.<br><br>Validated in BIG 1-98 trial<br>Postmenopausal women with hormone receptor positive early stage breast cancer. Treated with letrozole or tamoxifen. Women were included if they received chemotherapy.
Exclusion criteria:	NA
Original validation:	0.678

Variable name	Input	N (%) total = 5716*
Tumor size	mm (median (IQR))	17 (10 – 23)
Tumor grade	I	804 (14.1%)
	II	2599 (45.5%)
	III	1923 (33.6%)
	Missing	390 (6.8%)
Age	Years (mean (SD))	61 (13.8)
Positive lymph nodes	0	2112 (36.9%)
	1	1593 (27.9%)
	2 – 3	1079 (18.9%)
	4 – 9	636 (11.1%)
	>9	296 (5.2%)
Distant recurrence 5 – 10 years	No	5404 (94.5%)
	Yes	312 (5.5%)

* Including NAs for postmenopausal status and hormone receptor status.

AUC	0.574 (0.540 – 0.604)
Brier score	0.057 (0.052 – 0.062)
Scaled Brier score	-0.107 (-0.143 – -0.080)

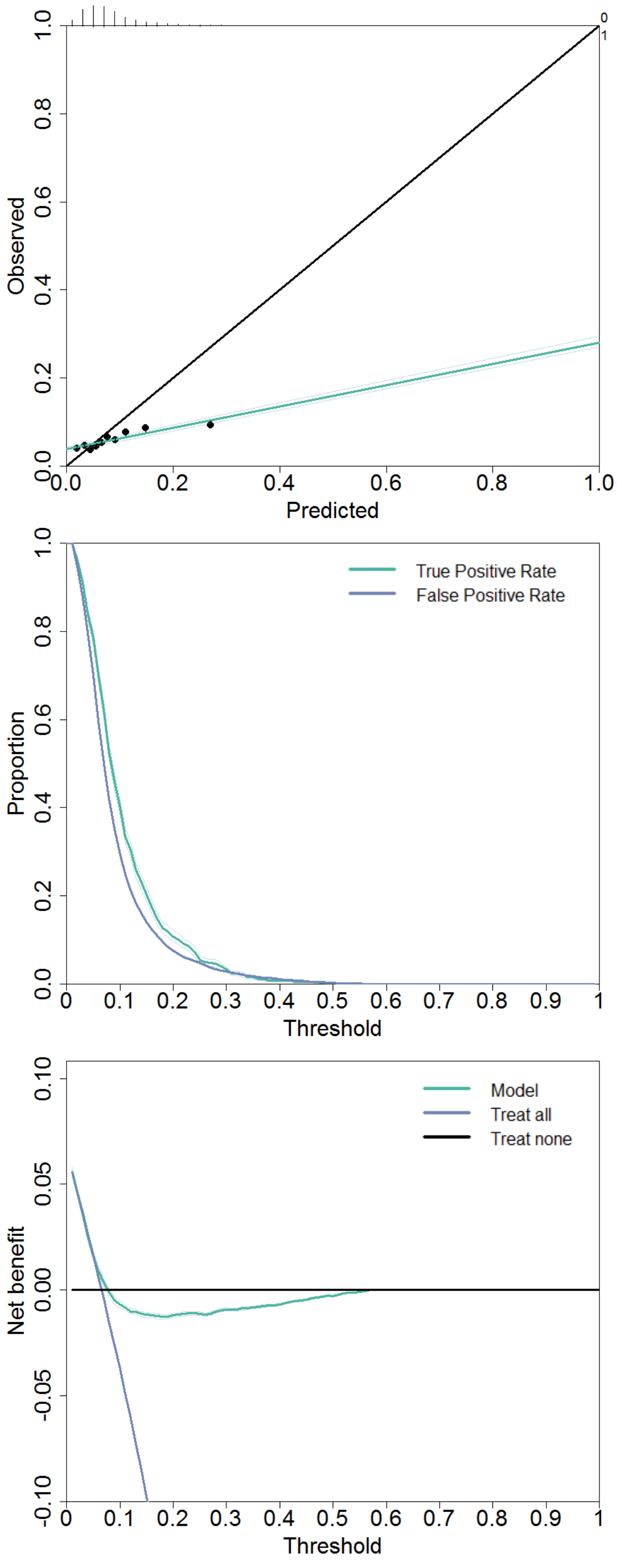

31. Lin et al. (Model 33)

Reference: *Lin, Z., Yan, S., Zhang, J., & Pan, Q. (2018). A nomogram for distinction and potential prediction of liver metastasis in breast cancer patients. Journal of Cancer, 9(12), 2098.*

Number of models presented in the paper: 1

Outcome:	Liver metastasis
Input variables:	Gender (male, female)<br>Age (continuous)<br>Histology (lobular, ductal)<br>N stage (N0, N1, N2, N3)<br>Tumor grade (I, II, III/IV)<br>ER status (negative, positive)<br>PR status (negative, positive)<br>HER2 status (unclear, negative, positive)
Inclusion criteria:	Primary malignant breast cancer<br>M1 stage at diagnosis
Exclusion criteria:	NA
Original validation:	0.66 (95% CI: 0.64 – 0.68) & 0.65 (95% CI: 0.63 – 0.67) for training and validation cohorts, respectively.

Variable name	Input	N (%) total = 10312
Gender	Male	91 (0.9%)
	Female	10221 (99.1%)
Age	Years (mean (sd))	63 (15)
Histology	Lobular	2056 (19.9%)
	Ductal	8256 (80.1%)
N stage	N0	3014 (29.2%)
	N1	5168 (50.1%)
	N2	399 (3.9%)
	N3	1731 (16.8%)
Tumor grade	I	434 (4.2%)
	II	2600 (25.2%)
	III/IV	2070 (20.1%)
	Missing	5208 (50.5%)
ER	Negative	2074 (20.1%)
	Positive	7607 (73.8%)
	Missing	631 (6.1%)
PR	Negative	4003 (38.8%)
	Positive	5484 (53.2%)
	Missing	825 (8%)
HER2	Negative	6668 (64.7%)
	Positive	1998 (19.4%)
	Unclear	957 (9.3%)
	Missing	689 (6.7%)
Liver metastasis	No	7760 (75.3%)
	Yes	2552 (24.7%)

AUC	0.652 (0.641 – 0.663)
Brier score	0.175 (0.172 – 0.180)
Scaled Brier score	0.056 (0.048 – 0.066)

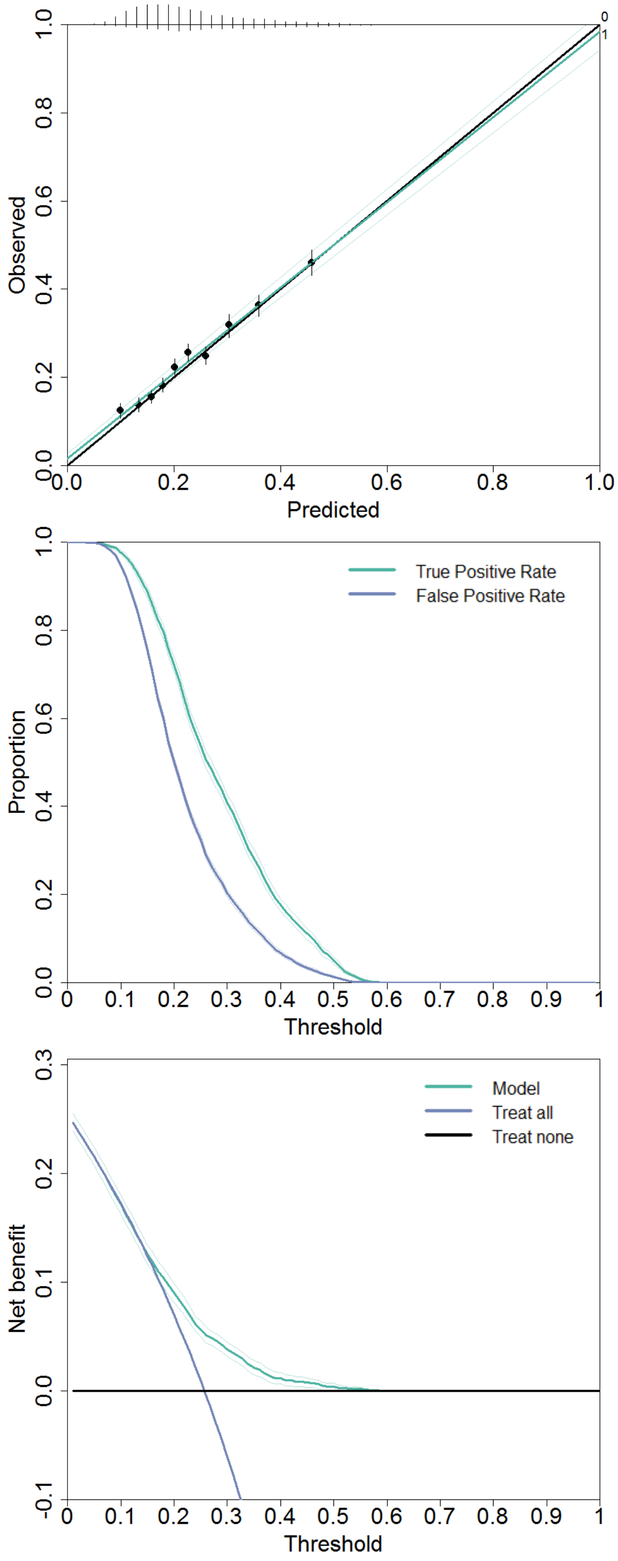

32. Lim et al. (Models 32a & 32b)

Reference: Lim, Y. J., Lee, S. W., Choi, N., Kwon, J., Eom, K. Y., Kang, E., ... & Kim, I. A. (2018). A novel prognostic nomogram for predicting risks of distant failure in patients with invasive breast cancer following postoperative adjuvant radiotherapy. Cancer research and treatment: official journal of Korean Cancer Association, 50(4), 1140.

Number of models presented in the paper: 2

Outcome:	5-year metastasis free interval<br>10-year metastasis free interval
Input variables:	Age (<45, ≥45)<br>Molecular subtype (Lum A, Lum B, HER2+, TNBC)<br>T stage (T1, T2, T3, T4)<br>pN stage (N0, N1, N2, N3)
Inclusion criteria:	Completed surgery and postoperative radiotherapy, Initial M0, no previous malignancy, no refusal of systematic/endocrine treatment, at least 1 year of follow-up.
Exclusion criteria:	NA
Original validation:	0.812

Variable	Input	5-year N = 24464	10-year N = 8601
Age	<45	3583 (14.6%)	1583 (18.4%)
	≥45	20881 (85.4%)	7018 (81.6%)
T stage	T1	15794 (64.6%)	5086 (59.1%)
	T2	7506 (30.7%)	2942 (34.2%)
	T3	897 (3.7%)	431 (5%)
	T4	267 (1.1%)	142 (1.7%)
N stage	N0	14377 (58.8%)	4529 (52.7%)
	N1	6223 (25.4%)	2237 (26%)
	N2	2516 (10.3%)	1096 (12.7%)
	N3	1348 (5.5%)	739 (8.6%)
Molecular subtype	Luminal A	13073 (53.4%)	3640 (42.3%)
	Luminal B	1767 (7.2%)	654 (7.6%)
	HER2+	1100 (4.5%)	409 (4.8%)
	Triple Negative	2422 (9.9%)	824 (9.6%)
	Missing	6102 (24.9%)	3074 (35.7%)
Metastasis free	No	2258 (9.2%)	2703 (31.4%)
	Yes	22206 (90.8%)	5898 (68.6%)

	5-year (32a)	10-year (32a)
AUC	0.748 (0.738 – 0.759)	0.735 (0.722 – 0.746)
Brier score	0.082 (0.079 – 0.085)	0.275 (0.267 – 0.282)
Scaled Brier score	0.026 (0.019 – 0.035)	-0.273 (-0.291 – 0.256)

5-year (32a) 10 year (32b)

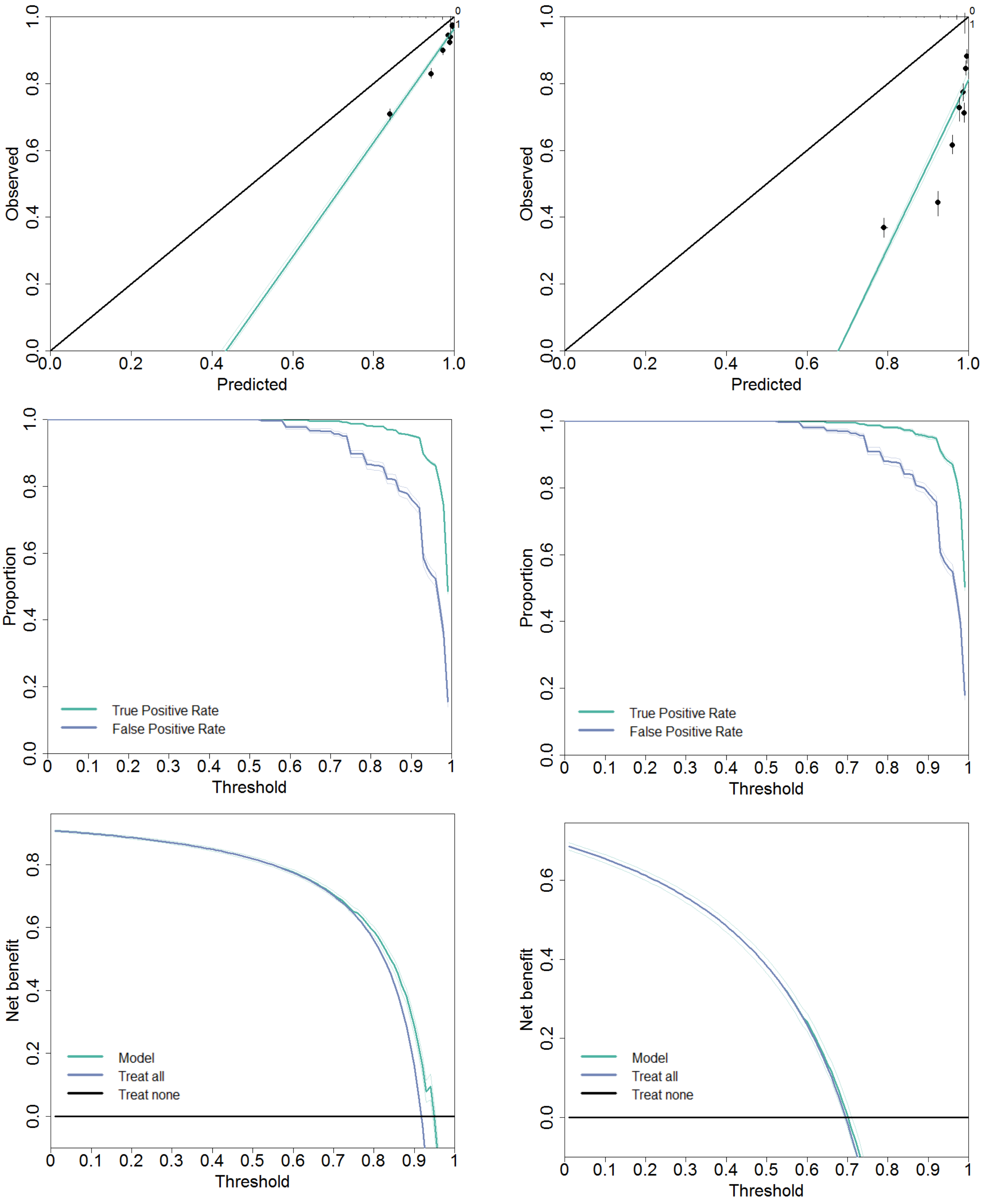

33. Boutros et al. (Model 33)

Reference: Boutros, C., Mazouni, C., Lerebours, F., Stevens, D., Lei, X., Gonzalez-Angulo, A. M., & Delaloge, S. (2015). A preoperative nomogram to predict the risk of synchronous distant metastases at diagnosis of primary breast cancer. *British journal of cancer*, *112*(6), 992-997.
Number of models presented in the paper: 1

Outcome:	Synchronous metastasis
Input variables:	Tumor size (continuous)<br>cN stage (cN0, cN+)<br>ER stage (Negative, positive)<br>PR stage (Negative, positive)
Inclusion criteria:	Women diagnosed with Invasive breast cancer
Exclusion criteria:	NA
Original validation:	0.861 and 0.638 in two external validation cohorts.

Variable name	Input	N (%) total = 243930
Tumor size	mm (median (IQR))	15 (10 – 23)
cN stage	cN0	194783 (79.9%)
	cN+	49147 (20.1%)
ER status	Negative	39043 (16%)
	Positive	196355 (80.5%)
	Missing	8532 (3.5%)
PR status	Negative	75487 (30.9%)
	Positive	156234 (64%)
	Missing	12209 (5%)
Synchronous metastasis	No	232501 (95.3%)
	Yes	11429 (4.7%)

AUC	0.783 (0.780 – 0.788)
Brier score	0.043 (0.043 – 0.044)
Scaled Brier score	0.028 (0.025 – 0.032)

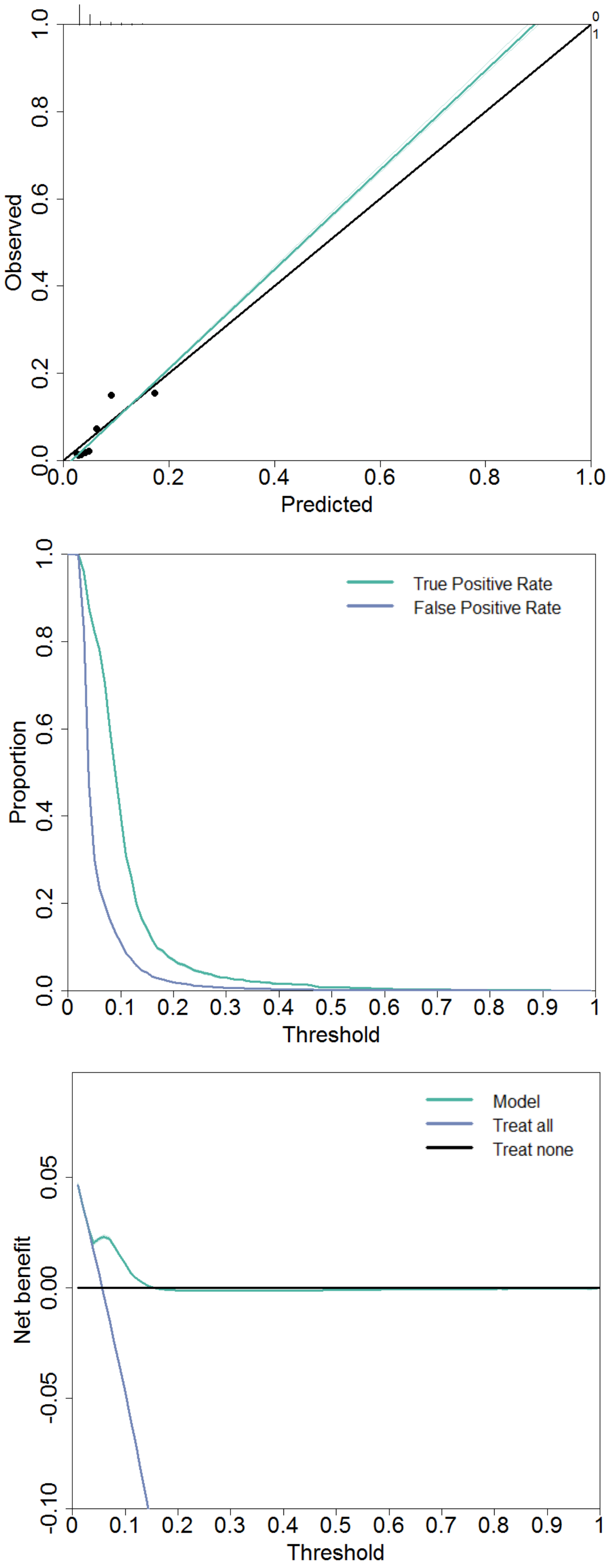

34. Zhang et al. (Model 34)

Reference: *Zhang, J., Li, X., Huang, R., Feng, W. L., Kong, Y. N., Xu, F., ... & Wang, K. (2017). A nomogram to predict the probability of axillary lymph node metastasis in female patients with breast cancer in China: a nationwide, multicenter, 10-year epidemiological study. Oncotarget, 8(21), 35311.*

Number of models presented in the paper: 1

**Outcome:**	Axillary lymph node involvement
**Input variables:**	Age (continuous)<br>Topography (Upper inner quadrant, upper outer quadrant, lower inner quadrant, lower outer quadrant, other)<br>Invasive disease (no, yes)<br>cN stage (cN0, cN+)<br>cT stage (cT1, cT2, cT3)<br>Histology (Ductal, Lobular, others)<br>Molecular subtype (Luminal, HER2+)
**Inclusion criteria:**	Underwent SLNB & ALND, female patients, diagnosed by histopathology
**Exclusion criteria:**	cT4, cNx
**Original validation:**	0.7157 & 0.7007 in the training and validation cohorts, respectively

**Variable name**	**Input**	**N (%)**<br>**total = 12873**
Age	Years (mean (sd))	57.7 (13.0)
Topography	UIQ	1361 (10.6%)
	UOQ	4921 (38.2%)
	LIQ	840 (6.5%)
	LOQ	1056 (8.2%)
	Central	982 (7.6%)
	Other	3713 (28.8%)
Invasive disease	No	93 (0.7%)
	Yes	12780 (99.3%)
cN stage	cN0	10144 (78.8%)
	cN+	2729 (21.2%)
cT stage	cT1	7210 (56.0%)
	cT2	4907 (38.1%)
	cT3	756 (5.9%)
Molecular subtype	Luminal	8102 (62.9%)
	HER2+	1502 (11.7%)
	Triple negative	868 (6.7%)
	Missing	2401 (18.7%)
Axillary lymph node involvement	No	2845 (22.1%)
	Yes	10028 (77.9%)

AUC	0.696 (0.687 – 0.704)
Brier score	0.201 (0.199 – 0.203)
Scaled Brier score	-0.168 (-0.196 – -0.147)

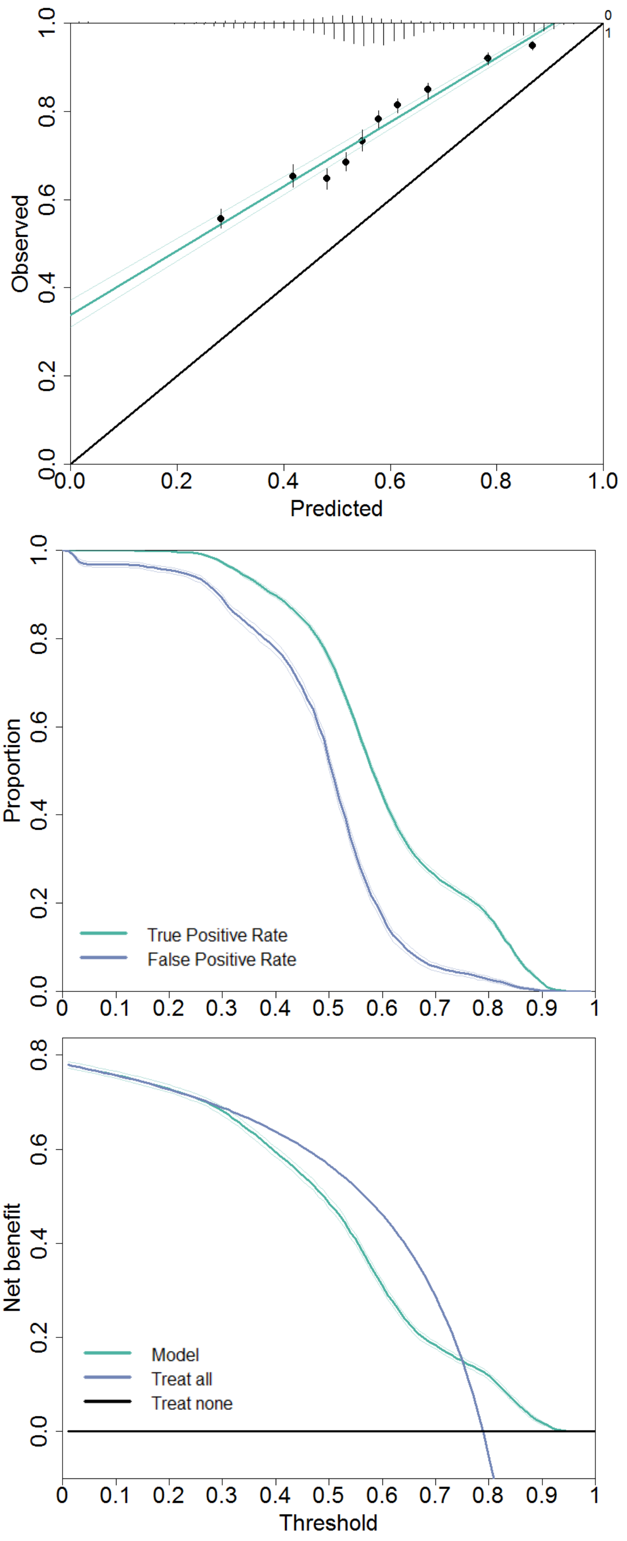

35. Meretoja et al. (Model 35)

Reference: Meretoja, T. J., Strien, L., Heikkilä, P. S., & Leidenius, M. H. K. (2012). A simple nomogram to evaluate the risk of nonsentinel node metastases in breast cancer patients with minimal sentinel node involvement. *Annals of surgical oncology*, *19*(2), 567-576.

Number of models presented in the paper:

Outcome:	Axillary lymph node metastasis
Input variables:	Multifocal tumor (No, Yes)<br>Tumor diameter (continuous)
Inclusion criteria:	Invasive breast cancer, patients with micrometastasis or ITC in their sentinel node. Underwent ALND
Exclusion criteria:	NA
Original validation:	0.682 (95% CI: 0.592 – 0.771) and 0.791 (0.637 – 0.945) in internal and external cohorts, respectively.

Variable name	Input	N (%) total = 5601
Multifocal	No	4407 (78.7%)
	Yes	1040 (18.6%)
	Missing	154 (2.7%)
Tumor size	mm (median (IQR))	18 (13 – 24)
ALND	No	4705 (84%)
	Yes	896 (16%)

AUC	0.596 (0.581 – 0.614)
Brier score	0.141 (0.132 – 0.150)
Scaled Brier score	-0.052 (-0.062 – -0.039)

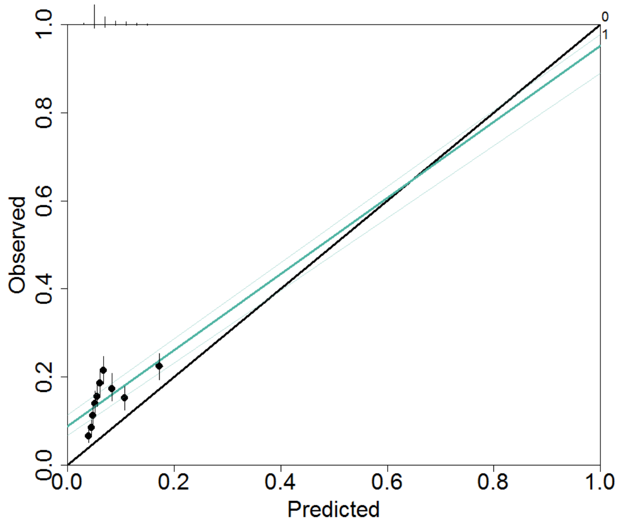

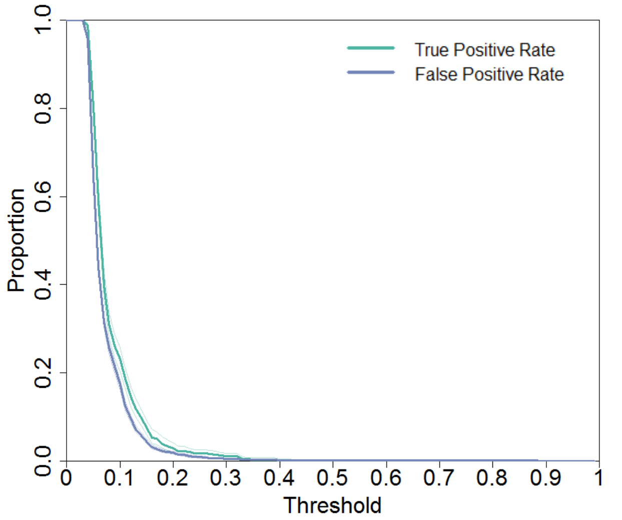

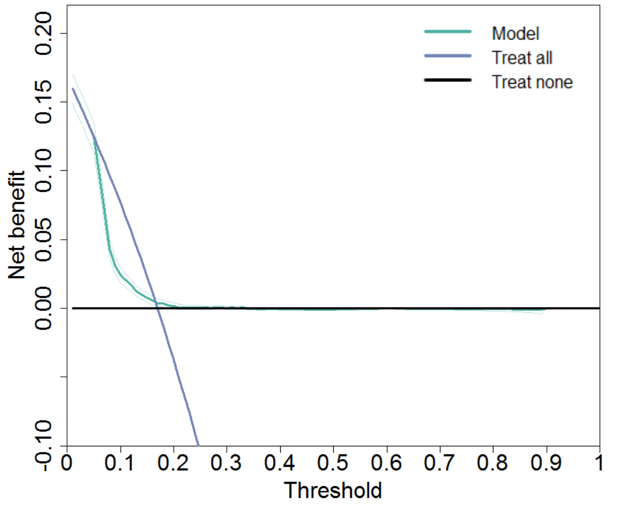

36. Houvanaeghel et al. (Model 36)

Reference: Houvenaeghel, G., Lambaudie, E., Classe, J. M., Mazouni, C., Giard, S., Cohen, M., ... & Boher, J. M. (2019). Lymph node positivity in different early breast carcinoma phenotypes: a predictive model. *BMC cancer*, *19*(1), 1-10.
Number of models presented in the paper: 4 models presented, 1 could be validated

Outcome:	Lymph node involvement
Input variables:	Age (≤40, 41-75, >75)<br>Tumor stage (T0-T1, T2, T3-T4)<br>Histological type (Lobular, Ductal, Mixed, Other)<br>Grade (I, II, III)<br>Subtype (Luminal A, Luminal B, HER2+, Triple negative)
Inclusion criteria:	Clinically node-negative invasive breast cancer.
Exclusion criteria:	Neoadjuvant therapy
Original validation:	0.682 (95% CI: 0.670 – 0.694) & 0.686 (95% CI: 0.682 – 0.690) on derivation and validation set, respectively

Variable name	Input	N (%) total = 164213
Age	≤40	7310 (4.5%)
	41 – 75	136407 (83.1%)
	>75	20496 (12.5%)
Tumor stage	T0	4067 (2.5%)
	T1	115319 (70.2%)
	T2	40943 (24.9%)
	T3	2930 (1.8%)
	T4	954 (0.6%)
Histological type	Lobular	19809 (12.1%)
	Ductal	125777 (76.6%)
	Mixed	5425 (3.3%)
	Other	13202 (8%)
Grade	I	42893 (26.1%)
	II	75220 (45.8%)
	III	39341 (24%)
	Missing	6759 (4.1%)
Molecular subtype	Luminal A	95528 (58.2%)
	Luminal B (HER2-)	17007 (10.4%)
	Luminal B (HER2+)	10143 (6.2%)
	HER2+	4445 (2.7%)
	TNBC	13154 (8%)
	Missing	12980 (7.9%)
LNI	No	124242 (75.7%)
	Yes	39971 (24.3%)

AUC	0.622 (0.619 – 0.625)
Brier score	0.203 (0.202 – 0.204)
Scaled Brier score	-0.101 (-0.106 – -0.095)

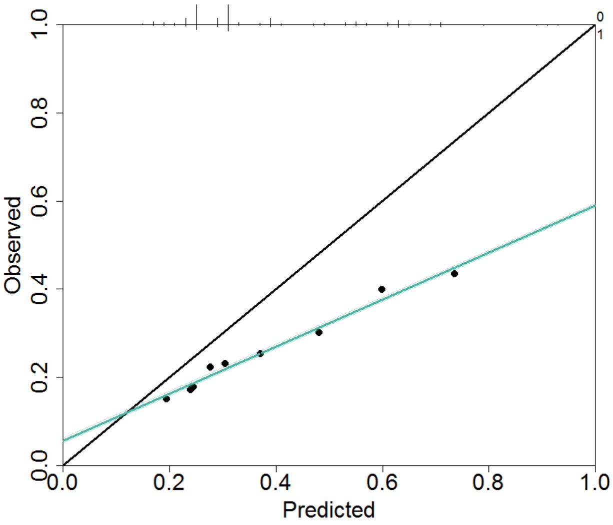

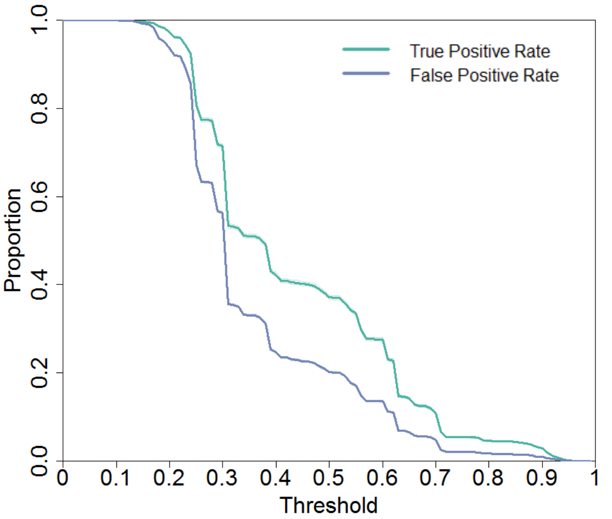

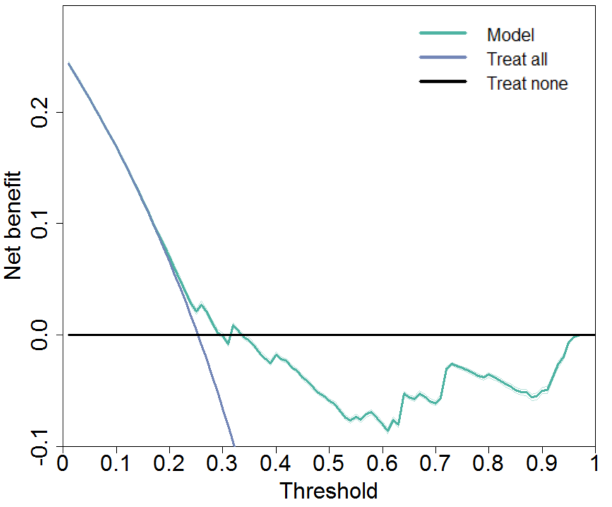

37. Schipper et al. (Model 37)

Reference: Schipper, R. J., Moossdorff, M., Nelemans, P. J., Nieuwenhuijzen, G. A., de Vries, B., Strobbe, L. J., ... & Smidt, M. L. (2014). A model to predict pathologic complete response of axillary lymph nodes to neoadjuvant chemo (immuno) therapy in patients with clinically node-positive breast cancer. *Clinical breast cancer*, *14*(5), 315-322.

Number of models presented in the paper:

Outcome:	Pathologic complete response of axillary lymph nodes
Input variables:	cT stage (cT 1-2, cT3, cT4)<br>Tumor type (Ductal, Lobular, Other type)<br>ER status (Negative, Positive)<br>PR status (Negative, Positive)<br>HER2 status (Negative, Positive)<br>Trastuzumab (No, Yes)<br>Taxane (No, Yes)
Inclusion criteria:	cN+, received neoadjuvant chemo(immune)therapy, Radical excision of the primary tumor and underwent ALND.
Exclusion criteria:	Nodal stage based on SLNB
Original validation:	C-index 0.77 (95% CI: 0.71 – 0.82)

Variable name	Input	N (%) total = 13422
cT	cT1	1748 (13%)
	cT2	6160 (45.9%)
	cT3	3284 (24.5%)
	cT4	2230 (16.6%)
Tumor type	Ductal	10864 (80.9%)
	Lobular	1304 (9.7%)
	Other	1254 (9.3%)
ER status	Negative	4181 (31.2%)
	Positive	9062 (67.5%)
	Missing	179 (1.3%)
PR status	Negative	6215 (46.3%)
	Positive	6928 (51.6%)
	Missing	279 (2.1%)
HER2 status	Negative	9386 (69.9%)
	Positive	3542 (26.4%)
	Missing	494 (3.7%)
Trastuzumab	No	10860 (80.9%)
	Yes	2562 (19.1%)
Taxane	No	6041 (45%)
	Yes	7381 (55%)
Complete response	No	9530 (71%)
	Yes	3892 (29%)

AUC	0.674 (0.662 – 0.684)
Brier score	0.198 (0.195 – 0.201)
Scaled Brier score	0.039 (0.023 – 0.056)

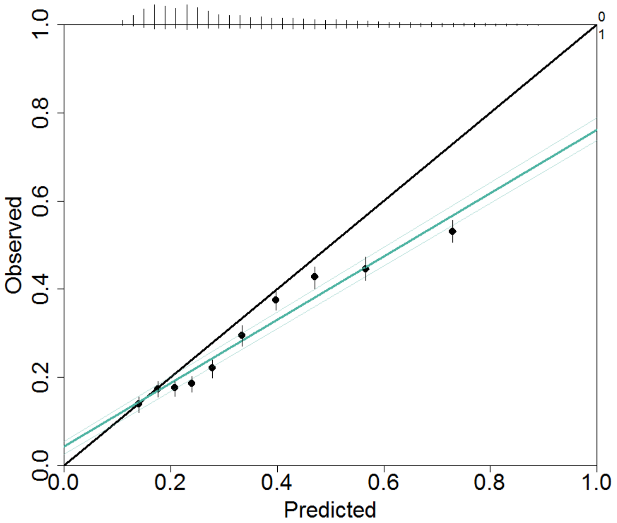

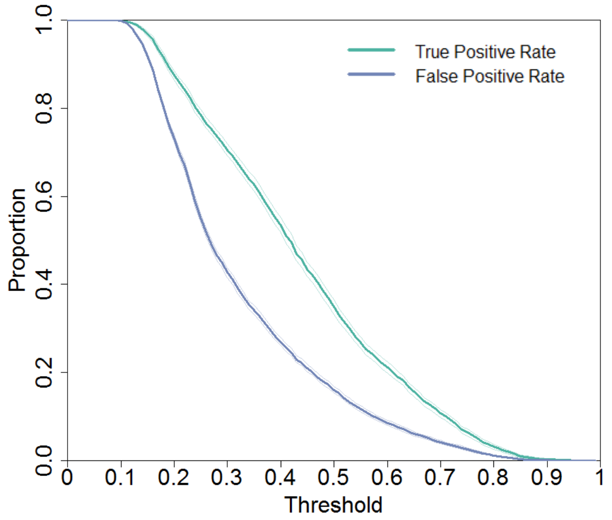

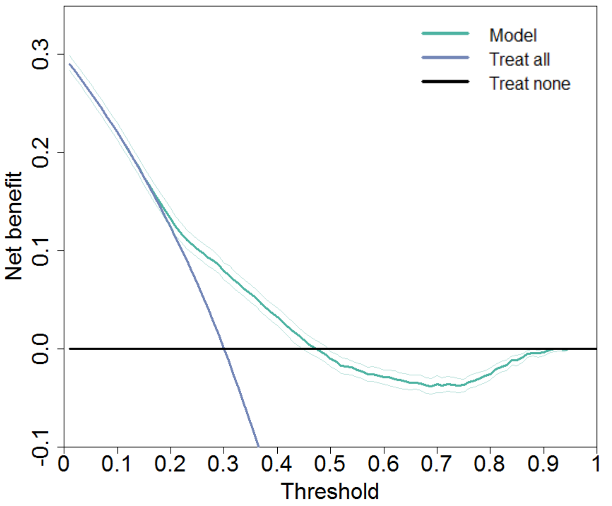

38. Pan et al. (Model 38)

Reference: *Pan, Z., Zhu, L., Li, Q., Lai, J., Peng, J., Su, F., ... & Chen, K. (2018). Predicting initial margin status in breast cancer patients during breast-conserving surgery. OncoTargets and therapy, 11, 2627.*
Number of models presented in the paper: 1

Outcome:	Positive surgical margin
Input variables:	HR status (negative, positive)<br>HER2 status (negative, positive)<br>Preoperative size (≤2cm, 2 – 5 cm, unknown)<br>cN stage (cN0, cN+)<br>Multifocality (absent, present)
Inclusion criteria:	Underwent breast conserving surgery
Exclusion criteria:	Metastatic disease/stage IV, Phyllodes tumor
Original validation:	0.72 (95% CI: 0.68 – 0.76) & 0.69 (95% CI: 0.64 – 0.75) for training and validation sets, respectively

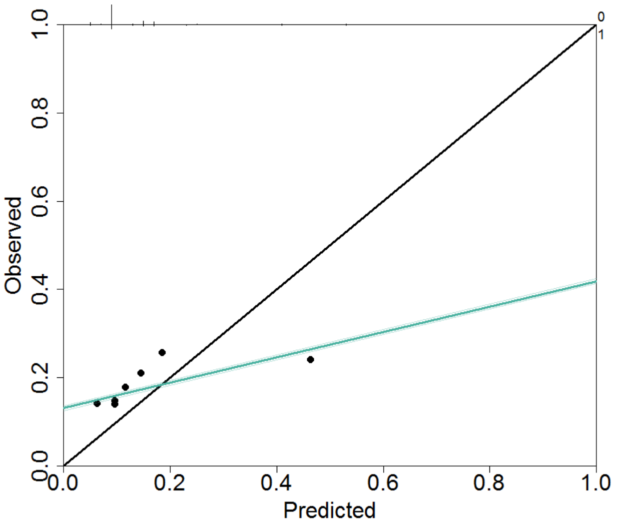

Variable name	Input	N (%) total = 113499
Hormone receptor status	Negative	13327 (11.7%)
	Positive	83741 (73.8%)
	Missing	16431 (14.5%)
HER2-status	Negative	80272 (70.7%)
	Positive	10999 (9.7%)
	Missing	22228 (19.6%)
Tumor size	≤2cm	86210 (76%)
	2 – 5 cm	23407 (20.6%)
	Missing	3882 (3.4%)
cN	cN0	103972 (91.6%)
	cN+	9527 (8.4%)
Multifocality	No	103178 (90.9%)
	Yes	9245 (8.1%)
	Missing	1076 (0.9%)
Positive surgical margin	No	93587 (82.5%)
	Yes	19912 (17.5%)

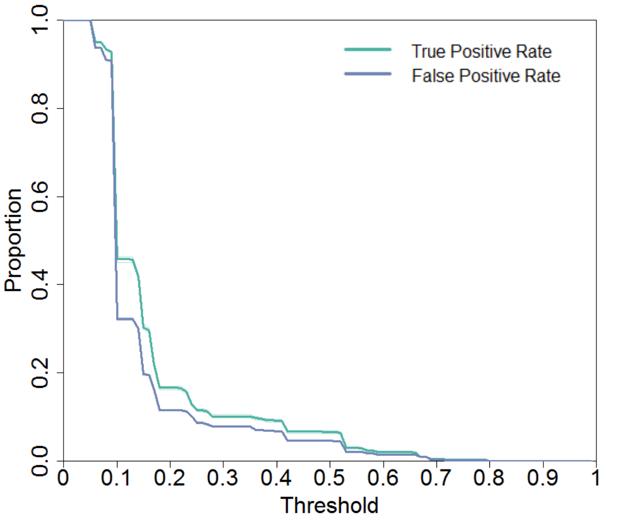

AUC	0.566 (0.562 – 0.570)
Brier score	0.154 (0.152 – 0.155)
Scaled Brier score	-0.064 (-0.068 – -0.060)

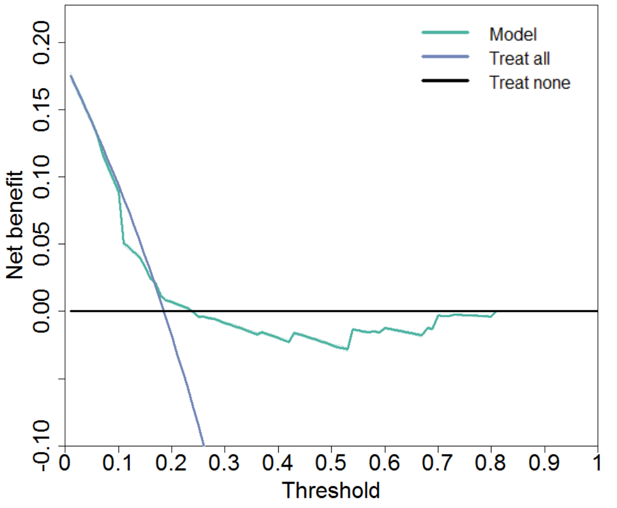

References

1. Xiong, Z., Deng, G., Huang, X., Li, X., Xie, X., Wang, J., ... & Wang, X. (2018). Score for the survival probability in metastasis breast cancer: a nomogram-based risk assessment model. Cancer research and treatment: official journal of Korean Cancer Association, 50(4), 1260.
2. Regierer, A. C., Wolters, R., Ufen, M. P., Weigel, A., Novopashenny, I., Köhne, C. H., ... & Wischnewsky, M. B. (2014). An internally and externally validated prognostic score for metastatic breast cancer: analysis of 2269 patients. Annals of oncology, 25(3), 633-638.
3. Fan, Y. P., Liu, C. L., Chiang, I. J., & Lin, C. Y. (2011). Development of a prognostic nomogram for identifying those factors which influence the 2-and 5-year survival chances of Taiwanese women diagnosed with breast cancer. European journal of cancer care, 20(5), 620-626.
4. Luo, C., Zhong, X., Wang, Z., Wang, Y., Wang, Y., He, P., ... & Zheng, H. (2019). Prognostic nomogram for patients with non-metastatic HER2 positive breast cancer in a prospective cohort. The International journal of biological markers, 34(1), 41-46.
5. Zhang, N., Zhang, J., Zhang, H., Liu, Y., Zhao, W., Wang, L., ... & Yang, Q. (2019). Individualized prediction of survival benefit from postmastectomy radiotherapy for patients with breast cancer with one to three positive axillary lymph nodes. The oncologist, 24(12), e1286.
6. Chen, Y. C., Lai, H. W., Wang, W. C., & Kuo, Y. L. (2016). Validation of breast Cancer survival prediction model with SEER database. J Integr Oncol, 5(3), 174.
7. Zhao, J., Yang, Y., Pang, D., Yu, Y., Lin, X., Chen, K., ... & Wang, Y. (2020). Development and validation of a nomogram in survival prediction among advanced breast cancer patients. Annals of Translational Medicine, 8(21).
8. Tang, Y., Zhang, Y. J., Zhang, N., Shi, M., Wen, G., Cheng, J., ... & Li, Y. X. (2020). Nomogram predicting survival as a selection criterion for postmastectomy radiotherapy in patients with T1 to T2 breast cancer with 1 to 3 positive lymph nodes. Cancer, 126, 3857-3866.
9. Xu, Y. B., Liu, H., Cao, Q. H., Ji, J. L., Dong, R. R., & Xu, D. (2020). Evaluating overall survival and competing risks of survival in patients with early-stage breast cancer using a comprehensive nomogram. Cancer medicine, 9(12), 4095-4106.
10. Wang, Z., Cheng, Y., Chen, S., Shao, H., Chen, X., Wang, Z., ... & Ye, Z. (2020). Novel prognostic nomograms for female patients with breast cancer and bone metastasis at presentation. Annals of translational medicine, 8(5).
11. Zheng, Y., Zhong, G., Yu, K., Lei, K., & Yang, Q. (2020). Individualized prediction of survival benefit from Locoregional surgical treatment for patients with metastatic breast Cancer. Frontiers in oncology, 10, 148.
12. Janssen, S., Haus, R., Schild, S. E., & Rades, D. (2020). A simple clinical instrument to predict the survival probability of breast cancer patients receiving radiotherapy for bone metastases. Anticancer research, 40(1), 367-371.
13. Wang, X., Feng, Z., Huang, Y., Li, H., Cui, P., Wang, D., ... & Chen, K. (2019). A nomogram to predict the overall survival of breast cancer patients and guide the postoperative adjuvant chemotherapy in China. Cancer management and research, 11, 10029.
14. Abdel-Rahman, O. (2018). M-bioscore: proposing a new statistical model for prognostic factors in metastatic breast cancer patients. Journal of comparative effectiveness research, 7(09), 845-854.
15. Elwood, J. M., Tawfiq, E., TinTin, S., Marshall, R. J., Phung, T. M., Campbell, I., ... & Lawrenson, R. (2018). Development and validation of a new predictive model for breast cancer survival in New Zealand and comparison to the Nottingham prognostic index. BMC cancer, 18(1), 1-12.
16. Paredes-Aracil, E., Palazón-Bru, A., Folgado-de la Rosa, D. M., Ots-Gutiérrez, J. R., Compañ-Rosique, A. F., & Gil-Guillén, V. F. (2017). A scoring system to predict breast cancer mortality at 5 and 10 years. Scientific reports, 7(1), 1-8.
17. Wen, J., Yang, Y., Liu, P., Ye, F., Tang, H., Huang, X., ... & Xie, X. (2017). Development and validation of a nomogram for predicting survival on the base of modified lymph node ratio in breast cancer patients. The Breast, 33, 14-22.

18. Wen, J., Ye, F., He, X., Li, S., Huang, X., Xiao, X., & Xie, X. (2016). Development and validation of a prognostic nomogram based on the log odds of positive lymph nodes (LODDS) for breast cancer. Oncotarget, 7(15), 21046.
19. Chen, S., Liu, Y., Yang, J., Liu, Q., You, H., Dong, Y., & Lyu, J. (2019). Development and validation of a nomogram for predicting survival in male patients with breast cancer. Frontiers in oncology, 9, 361.
20. Fu, R., Yang, J., Wang, H., Li, L., Kang, Y., Kaaya, R. E., ... & Lyu, J. (2020). A nomogram for determining the disease-specific survival in invasive lobular carcinoma of the breast: A population study. Medicine, 99(43).
21. Herrero-Vicent, C., Guerrero-Zotano, A., Gavilá-Gregori, J., Hernández-Blanquisett, A., Sandiego-Contreras, S., Samper-Hiraldo, J. M., ... & Ruiz-Simón, A. (2016). A prognostic index for locoregional recurrence after neoadjuvant chemotherapy. ecancermedicalscience, 10.
22. Wobb, J. L., Chen, P. Y., Shah, C., Moran, M. S., Shaitelman, S. F., Vicini, F. A., ... & Beitsch, P. (2015). Nomogram for predicting the risk of locoregional recurrence in patients treated with accelerated partial-breast irradiation. International Journal of Radiation Oncology* Biology* Physics, 91(2), 312-318.
23. Sanghani, M., Truong, P. T., Abi Raad, R., Niemierko, A., Lesperance, M., Olivotto, I. A., ... & Taghian, A. G. (2010). Validation of a web-based predictive nomogram for ipsilateral breast tumor recurrence after breast conserving therapy. Journal of clinical oncology, 28(5), 718.
24. Li, M., Yue, J., Wan, X., Hua, B., Yang, Q., Yang, P., ... & Xia, X. (2020). Risk-Adapted Postmastectomy Radiotherapy Decision Based on Prognostic Nomogram for pT1-2N1M0 Breast Cancer: A Multicenter Study. Frontiers in Oncology, 10.
25. Corso, G., Maisonneuve, P., Massari, G., Invento, A., Pravettoni, G., De Scalzi, A., ... & Veronesi, P. (2020). Validation of a novel Nomogram for prediction of local relapse after surgery for invasive breast carcinoma. Annals of surgical oncology, 1-11.
26. Li, S., Yu, K. D., Fan, L., Hou, Y. F., & Shao, Z. M. (2011). Predicting breast cancer recurrence following breast-conserving therapy: a single-institution analysis consisting of 764 Chinese breast cancer cases. Annals of surgical oncology, 18(9), 2492-2499.
27. Tokatli, Z. F., Türe, M., Ömürlü, İ. K., Alas, R. Ç., & Uzal, M. C. (2011). Developing and comparing two different prognostic indexes for predicting disease-free survival of nonmetastatic breast cancer patients. Turkish Journal of Medical Sciences, 41(5), 769-780.
28. Lin, H., Zhang, F., Wang, L., & Zeng, D. (2019). Use of clinical nomograms for predicting survival outcomes in young women with breast cancer. Oncology letters, 17(2), 1505-1516.
29. Paredes-Aracil, E., Palazón-Bru, A., Folgado-de la Rosa, D. M., Ots-Gutiérrez, J. R., Llorca-Ferrándiz, C., Alonso-Hernández, S., ... & Gil-Guillén, V. F. (2018). A scoring system to predict recurrence in breast cancer patients. Surgical oncology, 27(4), 681-687.
30. Dowsett, M., Sestak, I., Regan, M. M., Dodson, A., Viale, G., Thürlimann, B., ... & Cuzick, J. (2018). Integration of clinical variables for the prediction of late distant recurrence in patients with estrogen receptor–positive breast cancer treated with 5 years of endocrine therapy: CTS5. Journal of Clinical Oncology, 36(19), 1941.
31. Lin, Z., Yan, S., Zhang, J., & Pan, Q. (2018). A nomogram for distinction and potential prediction of liver metastasis in breast cancer patients. Journal of Cancer, 9(12), 2098.
32. Lim, Y. J., Lee, S. W., Choi, N., Kwon, J., Eom, K. Y., Kang, E., ... & Kim, I. A. (2018). A novel prognostic nomogram for predicting risks of distant failure in patients with invasive breast cancer following postoperative adjuvant radiotherapy. Cancer research and treatment: official journal of Korean Cancer Association, 50(4), 1140.
33. Boutros, C., Mazouni, C., Lerebours, F., Stevens, D., Lei, X., Gonzalez-Angulo, A. M., & Delaloge, S. (2015). A preoperative nomogram to predict the risk of synchronous distant metastases at diagnosis of primary breast cancer. British journal of cancer, 112(6), 992-997.
34. Zhang, J., Li, X., Huang, R., Feng, W. L., Kong, Y. N., Xu, F., ... & Wang, K. (2017). A nomogram to predict the probability of axillary lymph node metastasis in female patients with breast cancer in China: a nationwide, multicenter, 10-year epidemiological study. Oncotarget, 8(21), 35311.

35. Meretoja, T. J., Strien, L., Heikkilä, P. S., & Leidenius, M. H. K. (2012). A simple nomogram to evaluate the risk of nonsentinel node metastases in breast cancer patients with minimal sentinel node involvement. Annals of surgical oncology, 19(2), 567-576.
36. Houvenaeghel, G., Lambaudie, E., Classe, J. M., Mazouni, C., Giard, S., Cohen, M., ... & Boher, J. M. (2019). Lymph node positivity in different early breast carcinoma phenotypes: a predictive model. BMC cancer, 19(1), 1-10.
37. Schipper, R. J., Moossdorff, M., Nelemans, P. J., Nieuwenhuijzen, G. A., de Vries, B., Strobbe, L. J., ... & Smidt, M. L. (2014). A model to predict pathologic complete response of axillary lymph nodes to neoadjuvant chemo (immuno) therapy in patients with clinically node-positive breast cancer. Clinical breast cancer, 14(5), 315-322.
38. Pan, Z., Zhu, L., Li, Q., Lai, J., Peng, J., Su, F., ... & Chen, K. (2018). Predicting initial margin status in breast cancer patients during breast-conserving surgery. OncoTargets and therapy, 11, 2627.
